# Effect of sacubitril/valsartan and ACEI/ARB on glycaemia and the development of diabetes: a systematic review and meta-analysis of randomised controlled trials

**DOI:** 10.1186/s12916-022-02682-w

**Published:** 2022-12-17

**Authors:** Ruxin Wang, Haowen Ye, Yongting Zhao, Jinjing Wei, Ying Wang, Xiaofang Zhang, Lihong Wang

**Affiliations:** 1 Department of Endocrinology and Metabolism, The First Affiliated Hospital of Jinan University, No. 613, Huang pu Avenue West, Guangzhou, Guangdong China; 2 Department of Endocrinology, The Second Affiliated Hospital of Harbin Medical University, Harbin, Harbin, China; 3 Clinical Experimental Center, The First Affiliated Hospital of Jinan University, No. 613, Huang pu Avenue West, Guangzhou, Guangdong China; 4 The Guangzhou Key Laboratory of Basic and Translational Research on Chronic Diseases, The First Affiliated Hospital, Jinan University, Guangzhou, China

**Keywords:** Sacubitril/valsartan, Angiotensin-converting enzyme inhibitors (ACEI), Angiotensin-receptor blocker (ARB), Diabetes mellitus, Heart failure

## Abstract

**Background:**

Sacubitril/valsartan and angiotensin-converting enzyme inhibitor (ACEI)/angiotensin-receptor blocker (ARB) therapies were reported to affect glycaemic control and the development of diabetes mellitus (DM), but the findings are inconsistent. We examined the evidence for the effects of sacubitril/valsartan and ACEI/ARB in DM by conducting a meta-analysis.

**Methods:**

The Cochrane Central Register of Controlled Trials (The Cochrane Library), Embase, PubMed, and ClinicalTrials.gov were searched for data from randomised clinical trials (RCTs) that evaluated the efficacy of sacubitril/valsartan and ACEI/ARB in patients, as of May 25, 2022. Patients were grouped by their disease background at baseline. The main outcomes were the number of new-onset DM and hypoglycaemia, elevated glycaemia, inadequate DM control, diabetes treatment, and diabetic complications, from baseline to the end of the trials. The risk of bias was assessed using the revised Cochrane risk-of-bias tool for randomized trials (ROB 2). The quality of the evidence was evaluated according to the Recommendations for Assessment, Development, and Evaluation guidelines. The meta-analysis of the incidence of various outcomes was conducted using fixed or random effects models. The results are expressed as binary risk, 95% confidence interval (CI), and relative risk (RR). The Mantel-Haenszel method and *Z* test were used to determine the overall results and determine the significance of the RR.

**Results:**

This study included 31 RCTs and 86,809 subjects. Compared with placebo, sacubitril/valsartan treatment significantly reduced the risk of new-onset DM among all patients (RR = 0.78, 95% CI: 0.64–0.95), patients with heart failure (HF) (RR = 0.24, 95% CI: 0.12–0.48), HF with reduced ejection fraction (HFrEF) (RR = 0.24, 95% CI: 0.12–0.50), and HF with preserved ejection fraction (HFpEF) (RR = 0.54, 95% CI 0.34–0.85). In contrast, sacubitril/valsartan treatment significantly increased the risk of hypoglycaemia among all patients (RR = 1.91, 95% CI: 1.05–3.47), patients with not all-DM (defined as part of the study population having DM at baseline) (RR = 5.71, 95% CI: 2.02–16.21), and patients with HFpEF (RR = 7.06, 95% CI: 2.10–23.76). Compared with ACEI/ARB, sacubitril/valsartan treatment significantly increased the risk of hypoglycaemia among patients with HF (RR 1.85, 95% CI 1.12–3.06, *p* = 0.02) and HFpEF (RR 3.59, 95% CI 1.51–8.55, *p* = 0.004). Compared with placebo, ACEI/ARB treatment did significantly reduce the risk of new-onset DM among all patients (RR 0.85, 95% CI 0.77–0.93, *p* = 0.0007) and patients with not all-HF (defined as part of the study population having HF at baseline) (RR 0.87, 95% CI 0.82–0.93, *p*<0.0001) and HFpEF (RR 0.60, 95% CI 0.44–0.83, *p* = 0.002), diabetes complications among patients with non-HF (/not all-DM) (RR 0.87, 95% CI 0.76–0.99, *p* = 0.04), and subsequent diabetes treatment among patients with new-onset DM (RR 0.70, 95% CI 0.58–0.84, *p* = 0.0002) and significantly increased the risk of hypoglycaemia among patients with not all-DM (RR 2.06, 95% CI 1.172–3.61, *p* = 0.01).

**Conclusions:**

The results of our study, especially in reducing glycaemia and new-onset DM, revealed that sacubitril/valsartan had a positive effect on the control of glycaemia and the development of DM. ACEI/ARB also had a beneficial effect but the effect was weaker than that of sacubitril/valsartan. The above effects varied across diseases but the evidence was strongest in patients with HF.

**Trial registration:**

CRD42022336311.

**Supplementary Information:**

The online version contains supplementary material available at 10.1186/s12916-022-02682-w.

## Key point

### Question

Does sacubitril/valsartan or ACEI/ARB have an effect on glycaemia and the development of diabetes?

### Findings

In this meta-analysis of 31 randomised controlled trials that included 86,809 patients, compared with placebo, sacubitril/valsartan treatment significantly reduced the risk of new-onset diabetes (relative risk [RR] = 0.78, 95% confidence interval [CI] 0.64–0.95) and increased the risk of hypoglycaemia (RR = 1.91, 95% CI: 1.05–3.47); ACEI/ARB treatment significantly reduced the risk of new-onset diabetes (RR = 0.85, 95% CI: 0.77–0.93) and diabetes complications among patients with non-HF (RR = 0.87, 95% CI: 0.76–0.99) and diabetes treatment among patients with new-onset diabetes (RR = 0.70, 95% CI: 0.58–0.84) and increased the risk of hypoglycaemia among patients with not all-diabetes (RR = 2.06, 95% CI: 1.17–3.61).

### Meaning

Sacubitril/valsartan and ACEI/ARB had a positive effect on the control of glycaemia and the development of diabetes.

## Background

Diabetes mellitus (DM) is one of the major public health problems in the world today [1]. The latest global estimates from the International Diabetes Federation indicate that 537 million adults had DM in 2021, and that number is expected to increase to 643 million by 2030 [2]. DM often coexists with multiple diseases, especially in patients with heart failure (HF), in which the prevalence of diabetes is as high as 35–40% [3], and vice versa, leading to an adverse interactive effect on prognosis. The dual prevalence of DM and HF urgently requires effective treatments to address the increased burden in patients [4].

The role of angiotensin-converting enzyme inhibitors (ACEI)/angiotensin-receptor blockers (ARB) in glycaemic control and the development of diabetes has long been noted, but the mechanisms for improving glucose tolerance and insulin sensitivity by inhibiting the renin-angiotensin system are complex and unclear. Relevant trials [5–8] showed that ACEI/ARB could reduce new-onset diabetes and lower blood glucose levels compared with placebo. However, most outcomes other than new-onset diabetes did not reach statistical significance, the results were not uniform, and the research indicators of DM were relatively single.

The results of the PARADIGM-HF trial led to a landmark drug, sacubitril/valsartan, for the treatment of HF. The post hoc analysis of this trial suggested that sacubitril/valsartan treatment improved glycaemic control and conferred additional metabolic benefit [9]. Related preclinical studies also showed that dual ACE-neprilysin inhibitors could improve insulin sensitivity and improve glucose metabolism in obese rats and patients with insulin resistance [10–12]. The possible mechanisms for the role of sacubitril/valsartan in DM, in addition to the related effects of inhibiting the renin-angiotensin system, include decreasing the degradation of active peptides that can lower glycaemia by inhibiting neprilysin, and improving glucolipid metabolism and insulin resistance by consuming excessive energy by increasing natriuretic peptides [13–15]. Nevertheless, no specialised study on the mechanism and effects of sacubitril/valsartan in DM has been conducted.

Evaluating the role of sacubitril/valsartan and ACEI/ARB in DM, especially among patients with DM combined with HF, is a clinically meaningful issue. The purpose of this meta-analysis was to explore the effect of sacubitril/valsartan on glycaemia and the development of DM by analysing DM-related outcomes (new-onset DM, hypoglycaemia, elevated glycaemia, inadequate control DM, diabetes complications, and diabetes treatment) in randomised controlled trials (RCTs) and provide an updated analysis of the role of ACEI/ARB in treating diabetes. Furthermore, the results of this meta-analysis will help to provide physicians with information related to glycaemia and diabetes for use when treating patients with sacubitril/valsartan or ACEI/ARB.

### Ethics statement

All included studies were published without moral and informed consent disputes.

### Methods

All procedures strictly followed the Preferred Reporting Items for Systematic Reviews and Meta-Analyses (PRISMA). The application of this systematic review protocol for registration has been registered in the PROSPERO database (International Prospective Register of Systematic Reviews, https://www.crd.york.ac.uk/prospero), register number: CRD42022336311. The review method registered and updated in PROSPERO is described in Additional file 1 [16]. The evaluation of the quality of evidence was accorded to the Grading of Recommendations Assessment, Development, and Evaluation (GRADE) guideline.

#### Search strategy and identification

We searched the Cochrane Central Register of Controlled Trials (The Cochrane Library), Embase, PubMed, and ClinicalTrials.gov, the four major medical databases containing the majority of medical research literature, as of May 25, 2022. Two reviewers independently performed literature searches using search strategies designed by author RXW (the retrieval strategies are described in Additional file 2: Tables S1-S4). Publication date and language restrictions were not applied, and the reference lists of the related articles were also used to supplement the search terms. In addition, we use the appropriate filters (Additional file 2: Tables S1-S4). Duplicate articles were removed using reference manager software. Three reviewers independently identified the eligible studies according to pre-formulated inclusion and exclusion criteria (Additional file 2: Table S5). We included RCTs of adults in this meta-analysis if the control group was treated with ACEI/ARB/placebo and the experimental group was treated with sacubitril/valsartan or the control group was given a placebo and the experimental group was treated with ACEI/ARB.

#### Data extraction

Two reviewers independently extracted data from the RCTs that met the criteria and the guidelines in *Cochrane Reviewer’s Handbook*, and all authors discussed the results in the event of discrepancies. The research data were retrieved from the original published manuscript or the results in ClinicalTrials.gov. The following data were extracted from each trial: (1) name of the trial, author, and registration number; (2) year of publication; (3) number of participants enrolled; (4) characteristics of the participants at baseline, including DM status, age, and gender; (5) the drug used in the control group; (6) study duration; (7) main outcomes; and (8) the number of participants with new-onset DM, hypoglycaemia, hyperglycaemia, inadequate DM control, diabetes treatment, and diabetes complications from baseline to the end of the study.

#### Risk of bias assessment

Two researchers separately assessed the risk of bias for each qualified trial using the revised Cochrane risk-of-bias tool for randomized trials (ROB 2) and compiled a risk-of-bias table as described in the *Cochrane Handbook* [17].

#### Quality assessment

We used the GRADE principles to assess the quality of the evidence in this meta-analysis. The quality of the evidence was graded as very low, low, moderate, or high by measuring the risk of bias, inconsistency, indirectness, imprecision, and publication bias.

#### Outcome measures

A number of adverse reactions related to DM listed in the results of trials include new-onset DM, hypoglycaemia, elevated glycaemia, inadequate control DM, diabetes complications, and diabetes treatment, from baseline to the end of the trials. Among these, the number of new-onset DM cases and the remaining indicators reflected the effect of the drug on the development of diabetes and glycaemic control, respectively.

#### Statistical analyses

The data were analysed using Review Manager 5.4 and Stata 17.0. Direct comparisons of sacubitril/valsartan and ACEI/ARB groups and between ACEI/ARB and placebo groups were performed using Review Manager. A network meta-analysis of sacubitril/valsartan and placebo groups was performed using the ACEI or ARB group as an intermediate group and the “Network” program of Stata. Subgroup analysis was performed according to whether the patients had DM or HF (the included studies may have used different criteria for HF, and we did not use a standardised definition for HF) at baseline. Sensitivity analysis and publication bias detection were performed using Stata, and *I*^2^ was used to assess heterogeneity. An *I*^2^ value of > 50% or a corresponding *p*-value of < 0.05 was considered to indicate heterogeneity among the studies. In that case, we used a random model and performed meta-regression and subgroup analysis. An *I*^2^ of ≤ 50% and a corresponding *p*-value of ≥ 0.05 were considered to indicate no obvious heterogeneity in the results, and a fixed model was used [18]. Due to the lack of direct comparison, there was no need to test for inconsistency in the network meta-analysis. The data were extracted from each trial and expressed as binary risk. The 95% confidence interval (CI) and relative risk (RR) were used in the synthesis or presentation of the results. The Mantel-Haenszel method and the *Z* test were used to determine the overall results and the significance of the RR. A *p*-value of < 0.05 was considered statistically significant. All results were consistent with the PRISMA (Additional file 3) and meta-analysis guidelines [19].

#### Publication bias and sensitivity analysis

Funnel plots and Egger’s test [20] were used to detect publication bias using Stata, as was sensitivity analysis.

## Results

### Description of the selected studies

Initially, 21,836 possible articles or studies were identified, and 2973 possible articles were left after filtering and removing duplicates. The remaining articles were judged by the three researchers according to the inclusion and exclusion criteria. Finally, 31 RCTs including 86,809 subjects were included in the analysis. The flow chart of study selection is shown in Additional file 2: Fig. S1.

### Study characteristics

The detailed characteristics of the 31 trials [6–8, 21–45] are shown in Table 1, of which, most were large, multi-centre clinical studies, with 13 and 5 trials aimed at patients with HF and DM, respectively. In the patients with HF, the EF range for HFrEF was defined as an EF of ≤ 40% or 35%. HFpEF was defined as an EF of ≥ 40% or 45%. The total number of subjects was 86,809 and the follow-up period ranged from 8 weeks to 6.5 years. Randomised assignments were made using computer-generated random numbers in most of the trials and pre-specified outcomes were reported in all trials. Only one study (TRAFIC) did not use blinding.

**Table 1:** Characteristics of included RCTs

Trial	Number	Follow-up	Patient	Inclusion criteria	Age (years)	Male (%)	ACEI/ARB	Dosage	Baseline DM (%)	Main outcome
EVALUATE-HF	464	12 weeks	HFrEF	Age ≥ 50 years, hypertension, CHF, and EF ≤ 40%, NYHA I–III	67.8 ± 9.8 vs 66.7 ± 8.5	355 (77)	Enalapril	200mg bid vs 10mg bid		Treatment of HFrEF with sacubitril/valsartan, compared with enalapril, did not significantly reduce central aortic stiffness
NCT01785472	1434	8 weeks	Not all-HF	Mean sitting SBP ≥ 140 to < 180 mm Hg	(57.5 ± 10.17, 58.1 ± 9.71) vs 57.4 ± 10.14	756 (53)	Olmesartan	200/400 mg qd vs 20mg qd	156 (16) vs 77 (16)	Treatment with sacubitril/valsartan once daily is effective and provided superior BP reduction than olmesartan in Asian patients with mild-to-moderate hypertension
PARAMOUNT	301	3 months	HFpEF	NYHA II–III HFpEF, EF > 45%	70.9 ± 9.4 vs 71.2 ± 8.9	152 (57)	Valsartan	200mg bid vs 160mg bid	61 (41) vs 53 (35)	Sacubitril/valsartan has a better effect on reducing BNP and improving LA reverse remodeling and NYHA compared with valsartan in patients with HFpEF
PIONEER-HF	875	8 weeks	HFrEF	Hemodynamic stabilisation after ADHF and EF ≤ 40%	61 (51, 71) vs 63 (54, 72)	635 (72.1)	Enalapril	200mg bid vs 10mg bid	79 (18) vs 89 (20)	Among patients with HFrEF who were hospitalised for ADHF, the initiation of sacubitril/valsartan therapy led to a greater reduction in the NT-proBNP concentration than enalapril therapy
OUTSTEP-HF	619	12 weeks	HFrEF	NYHA II and LVEF ≤ 40%	66.89 ± 10.74	487 (79)	Enalapril	200mg bid vs 10mg bid	96 (31) vs 117 (38)	There was no significant benefit of sacubitril/valsartan either 6MWT or in daytime physical activity measured by actigraphy compared with enalapril
PARALLEL-HF	223	33.9 months	HFrEF	NYHA II–IV and EF ≤ 35%	69.0 ± 9.7 vs 66.7 ± 10.9	192 (86)	Enalapril	200mg bid vs 10mg bid	52 (47) vs 52 (46)	In Japanese patients with HFrEF, there was no difference in reduction in the risk of cardiovascular death or HF hospitalisation between sacubitril/valsartan and enalapril
PARALLAX	2564	24 weeks	HFpEF	NYHA II–IV, EF > 40%, LV hypertrophy or left atrial enlargement with NT-proBNP↑	73 ± 8.4 vs 72 ± 8.6	1265 (49)	Enalapril/valsartan	200mg bid vs 10mg bid vs 160mg	566 (44) vs 589 (46)	Among patients with HFpEF, sacubitril/valsartan treatment compared with standard renin-angiotensin system inhibitor treatment or placebo resulted in a significantly greater decrease in NT-proBNP levels at 12 weeks but did not significantly improve 6MWT at 24 weeks
PARADIGM-HF	8442	27 months	HFrEF	NYHA II–IV, EF ≤ 40%	63.8 ± 11.5 vs 63.8 ± 11.3	6567 (78)	Enalapril	200mg bid vs 10mg bid	1451 (35) vs 1456 (35)	Sacubitril/valsartan was superior to enalapril in reducing the risk of death and of hospitalisation for HFrEF
PARAGON-HF	4821	26 months	HFpEF	NYHA II–IV, EF ≥ 45%	72.7 ± 8.3 vs 72.8 ± 8.5	2317 (48)	Valsartan	200mg bid vs 160mg bid	1046 (44) vs 1016 (43)	Sacubitril/valsartan did not result in a significantly lower rate of total hospitalisations for HF and death from cardiovascular causes among patients with HFpEF
UK HARP-III	414	12 months	Not all-HF	eGFR ≥ 45 and < 60 mL/min/1.73m^2^ and uACR > 20; or eGFR ≥ 20 and < 45 mL/min/1.73m^2^	62.0 ± 14.1 vs 63.6 ± 13.4	298 (72)	Irbesartan	200mg bid vs 300mg qd	81 (39) vs 83 (40)	In people with chronic kidney disease, sacubitril/valsartan is well-tolerated and has similar effects on kidney function and albuminuria to irbesartan
HFN-LIFE	335	24 weeks	HFrEF	Advanced HFrEF, SBP ≥ 90 mmHg, NT-proBNP ≥ 800 pg/mL or BNP ≥ 250 pg/mL	60.2 ± 13.4 vs 58.3 ± 13.1	245 (73)	Valsartan	200mg bid vs 160mg bid	74 (44) vs 83 (49)	In patients with chronic advanced HFrEF, there was no statistically significant difference between sacubitril/valsartan and valsartan with respect to reducing NT-proBNP levels
AARDVARK	224	2 years	Not all-HF	Abdominal aortic aneurysms, SBP < 150 mmHg	71.6 ± 6.9 vs 70.7 ± 7.5	211 (94.2)	Perindopril	10mg qd vs 10mg qd	2 (3) vs 8 (10)	No evidence that in patients with systolic BP of <150 mmHg, the rate of growth of small AAAs is slowed by the administration of the ACE-I perindopril compared with placebo and that modest BP lowering did not beneficially impact on the growth of small AAAs
NCT00591253	412	6 weeks	Non-HF	Essential hypertension, SBP 150–180 mm Hg and 24-h mean SBP 130–170 mm Hg	<45: 66 vs 36 46–64: 180 vs 85 >65: 29 vs 17	177 (42.9)	Azilsartan	40/80 mg qd vs 40/80 mg qd		
TRAFIC	58	48 weeks	Non-HF	Infected with HIV	47 vs 50	41 (93.2)	Elmisartan	40-80 mg qd vs 40–80 mg qd		
LIFE-HIV	108	12 months	Non-HF	HIV infection, age > 50 years, SBP > 120 mmHg, GFR > 30 mL/min/1.73 m^2^	56 (53, 62) vs 56 (53, 61)	104 (96)	Losartan	100 mg qd vs 100 mg qd	0 (0) vs 0 (0)	Among older PHIV with viral suppression, losartan did not improve blood measures of inflammation nor T-cell immune recovery
ACTIVE I	9016	4.1 years	Not all-HF	SBP of at least 110 mmHg	69.5 ± 9.7 vs 69.6 ± 9.7	5475 (60.7)	Irbesartan	300 mg qd vs 300 mg qd	906 (20) vs 881 (20)	Irbesartan did not reduce cardiovascular events in patients with atrial fibrillation
I-Preserve	4126	49.5 months	HFpEF	Age ≥ 50 years and NYHA II, III, or IV HF and an EF ≥ 45%	72 ± 7 vs 72 ± 7	1637 (39.7)	Irbesartan	300 mg qd vs 300 mg qd	564 (27) vs 570 (28)	Irbesartan did not improve the outcomes of patients with heart failure and a preserved left ventricular ejection fraction
DIRECT-Prevent	1420	4.7 years	Not all-HF	T1D diagnosed before age of 36 years and in need for continuous insulin treatment within 1 year of diagnosis of diabetes	29.6 ± 8.0 vs 29.9 ± 8.1	805 (56.7)	Candesartan	32 mg qd vs 32 mg qd	710 (100) vs 710 (100)	Candesartan reduces the incidence of retinopathy
DIRECT-Protect 1	1902	4.8 years	Not all-HF	Aged 18–55 years with T1D diagnosed before age of 36 years and in need for continuous insulin treatment within 1 year of diagnosis of diabetes	31.5 ± 8.5 vs 31.9 ± 8.5	1091 (57.3)	Candesartan	32 mg qd vs 32 mg qd	951 (100) vs 951 (100)	We did not see a beneficial effect on retinopathy progression from candesartan
DIRECT-Protect 2	1902	4 years	Not all-HF	Aged 37–75 years with T2D diagnosed at age of 36 years or thereafter	56.9 ± 7.6 vs 56.8 ± 7.9	948 (49.8)	Candesartan	32mg qd vs 32 mg qd	949 (100) vs 953 (100)	Treatment with candesartan in T2D patients with mild to moderate retinopathy might induce improvement of retinopathy
ORGENT	565	3.4 years	Not all-HF	Clinical diagnosis of diabetic nephropathy in patients with T2D	59.1 ± 8.1 vs 59.2 ± 8.1	391 (69.1)	Olmesartan	10/20 mg qd vs 10/20 mg qd	287 (100) vs 288 (100)	In T2D patients with overt nephropathy and renal insufficiency receiving concomitant antihypertensive agents including ACEI, treatment with olmesartan reduced proteinuria and BP but did not further improve renal outcomes
Navigator	4599	5.0/6.5 years	Not all-HF	Impaired glucose tolerance and either cardiovascular disease or cardiovascular risk factors to receive nateglinide	63.7 ± 6.91 vs 63.9 ± 6.88	2318 (49.7)	Valsartan	160 mg qd vs 160 mg qd	0 (0) vs 0 (0)	Among persons with impaired glucose tolerance and established cardiovascular disease or cardiovascular risk factors, assignment to nateglinide for 5 years did not reduce the incidence of diabetes or the coprimary composite cardiovascular outcomes
HOPE (no-DM)	5720	4.5 years	Non-HF	Older than 55 years without known diabetes but with vascular disease	66.3 ± 6.7 vs 65.9 ± 6.9	4562 (79.7)	Ramipril	2.5 mg qd vs 2.5 mg qd	0 (0) vs 0 (0)	Ramipril is associated with lower rates of new diagnosis of diabetes in high-risk individuals
MICRO-HOPE	3577	4.5 years	Non-HF	Had evidence of vascular disease plus one other cardiovascular risk factor and not a low EF or HF, and diabetes	65.3 ± 6.4 vs 65.6 ± 6.6	2255 (63.0)	Ramipril	2.5 mg qd vs 2.5 mg qd	1808 (100) vs 1769 (100)	Ramipril was beneficial for cardiovascular events and overt nephropathy in people with diabetes. The cardiovascular benefit was greater than that attributable to the decrease in blood pressure
SCOPE	4923	3.7 years	Not all-HF	Aged 70–89 years, with SBP 160–179 mmHg, and/or DBP 90–99 mmHg, and a MMSE test score > 24	76.4 vs 76.4	1748 (35.5)	Candesartan	8 mg qd vs 8 mg qd	308 (12) 285 (12)	In elderly hypertensive patients, a slightly more effective blood pressure reduction during candesartan-based therapy, compared with control therapy, was associated with a modest, statistically nonsignificant, reduction in major cardiovascular events and with a marked reduction in non-fatal stroke
DREAM	5269	3 years	Non-HF	Without cardiovascular disease but with impaired fasting glucose levels (after an 8-h fast) or impaired glucose tolerance	54.7 ± 10.9 vs 54.7 ± 10.9	2149 (40.8)	Ramipril	15 mg qd vs 15 mg qd	0 (0) vs 0 (0)	Among persons with impaired fasting glucose levels or impaired glucose tolerance, the use of ramipril for 3 years does not significantly reduce the incidence of diabetes or death but does significantly increase regression to normoglycaemia
TRANSCEND	5926	56 months	Non-HF	Intolerant to ACEI with cardiovascular disease or diabetes with end-organ damage	66.9 ± 7.3 vs 66.9 ± 7.4	3379 (57.0)	Telmisartan	80 mg qd vs 80 mg qd	1059 (36) vs 1059 (36)	Telmisartan could be regarded as a potential treatment for patients with vascular disease or high-risk diabetes, if they are unable to tolerate an ACE inhibitor
CHARM (non-DM)	5436	2–4 years	HF	Complementary populations of patients with symptomatic HF not known to have DM at baseline	66 ± 11 vs 66 ± 12	1685 (31.0)	Candesartan	32 mg qd vs 32 mg qd	0 (0) vs 0 (0)	The angiotensin-receptor blocker candesartan appears to prevent diabetes in HF patients
SOLVD (non-DM)	291	3.4 years	HFrEF	Asymptomatic left ventricular dysfunction with congestive HF (EF ≤ 35%)	56.1 ± 10.1 vs 56.8 ± 10.0	268 (92.1)	Enalapril	5/20 mg qd vs 5/20 mg qd	0 (0) vs 0 (0)	Enalapril significantly reduces the incidence of diabetes in patients with left ventricular dysfunction, especially those with impaired fasting plasma glucose
PEACE	8290	7 years (median, 4.8)	Not all-HF	Stable coronary artery disease and normal or near-normal left ventricular function	64 ± 8 vs 64 ± 8	6798 (82.0)	Trandopril	4 mg qd vs 4 mg qd	748 (18) vs 661 (16)	In patients with stable coronary heart disease and preserved left ventricular function who are receiving “current standard” therapy and in whom the rate of cardiovascular events is lower than in previous trials of ACE inhibitors in patients with vascular disease, there is no evidence that the addition of an ACE inhibitor provides further benefit in terms of death from cardiovascular causes, myocardial infarction, or coronary revascularisation
Jean	2553	2.95 years (median)	Not all-HF	Post-CABG 7 d, stable after operation, 18 y old, LVEF ≥ 40%	61 ± 10 vs 61 ± 10	2229 (87)	Quinapril	40 mg qd vs 40 mg qd	121 (9) vs 132 (10)	In patients at low risk of cardiovascular events after CABG, routine early initiation of ACEI therapy does not appear to improve clinical outcome up to 3 years after CABG

*eGFR* estimated glomerular filtration rate, *SCr* serum creatinine, *uACR* urine albumin to creatinine ratio, *BP* blood pressure, *SBP* systolic blood pressure, *NT-proBNP* N-terminal pro-B type natriuretic peptide, *NYHA* New York Heart Association Functional Classification, *LV* left ventricle, *EF* ejection fraction, *HF* heart failure, *HFpEF* heart failure with preserved ejection fraction, *HFrEF* heart failure with reduced ejection fraction, *ADHF* acute heart failure, *MR* mitral regurgitation; ↑ increase, ↓ reduce, *6MWT* 6-min walk distant, *T1D* type 1 diabetes, *T2D* type 2 diabetes, *MMSE* Mini-Mental State Examination, *CABG* coronary artery bypass surgery, *AAA* abdominal aortic aneurysm, *not all-DM* defined as part of the study population having DM at baseline, *not all-HF* defined as part of the study population having HF at baseline

### Risk of bias assessment

The quality assessment of the included studies is presented in Additional file 4: Figs. S1-S2. One (0.5% weighting of all studies) RCT included in this meta-analysis revealed a high risk of bias when assessed by ROB 2, whereas twenty-six (81.4% weighting) RCTs raised some concerns and four (18.1% weighting) RCTs revealed a low risk of bias. This was mainly due to the fact that the definition and standard measures of the outcomes we studied were not elaborated in part of the studies.

### Clinical outcome evaluation

**Fig. 1:**
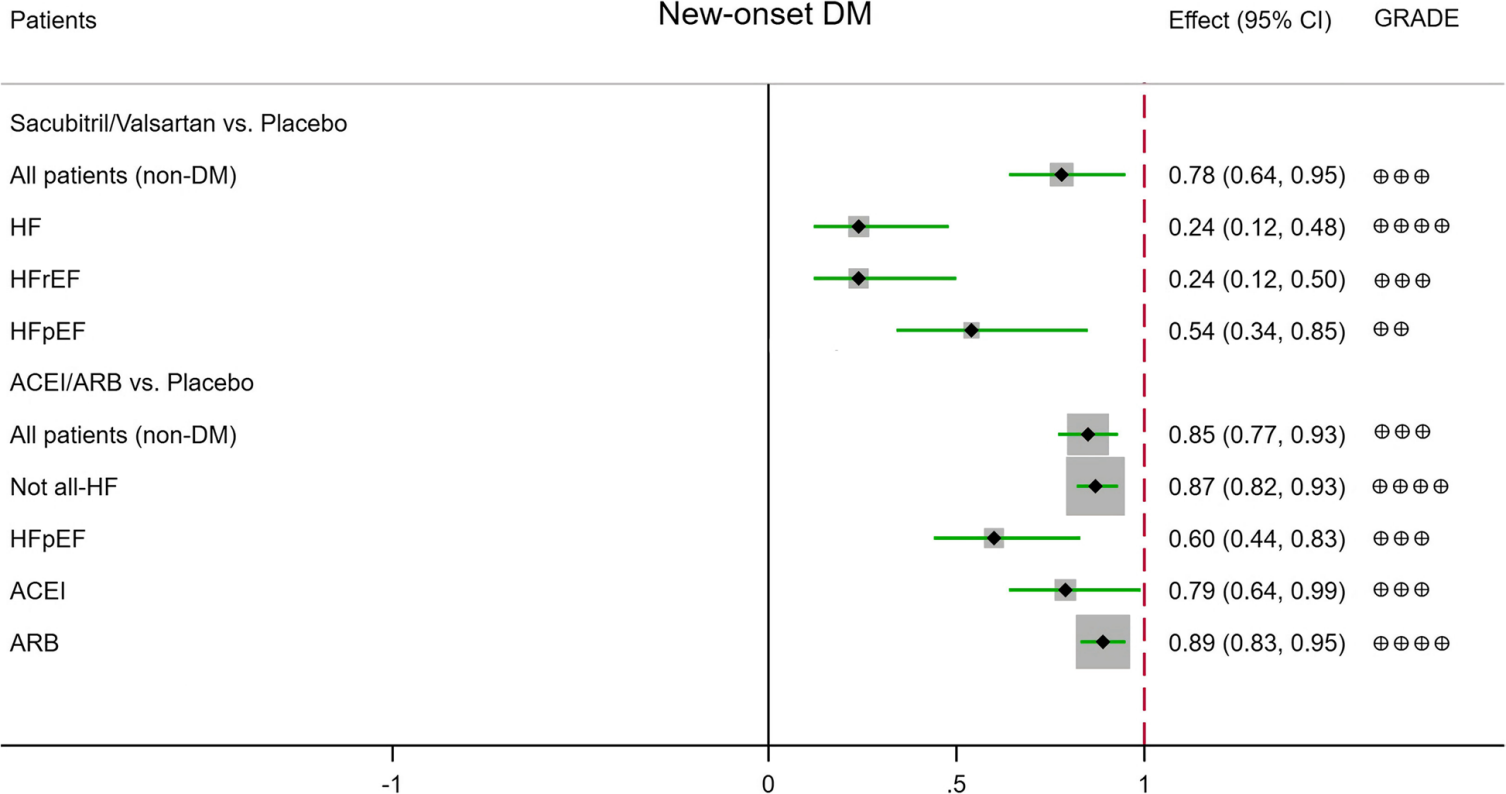
The effect of sacubitril/valsartan or ACEI/ARB on new-onset DM

**Fig. 2:**
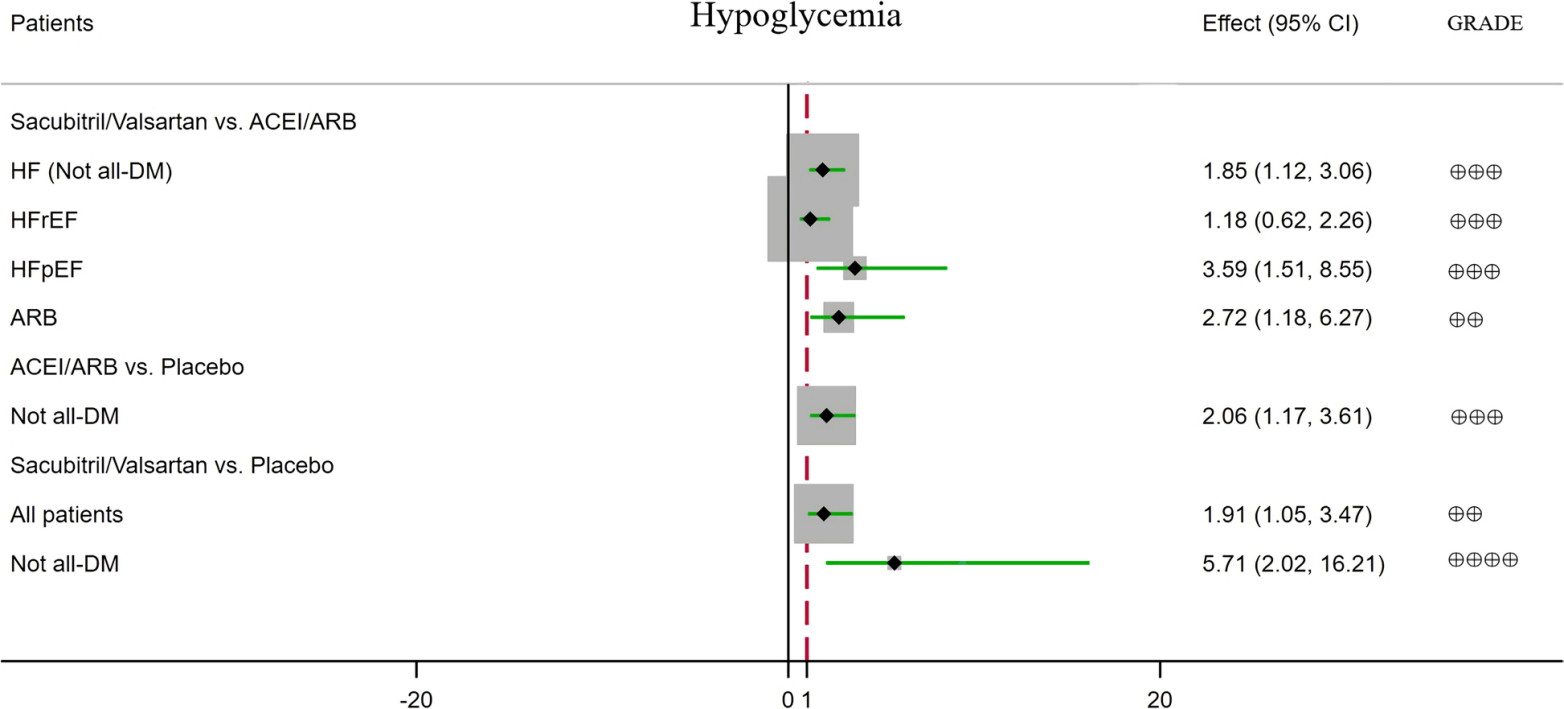
The effect of sacubitril/valsartan or ACEI/ARB on hypoglycaemia

**Fig. 3:**
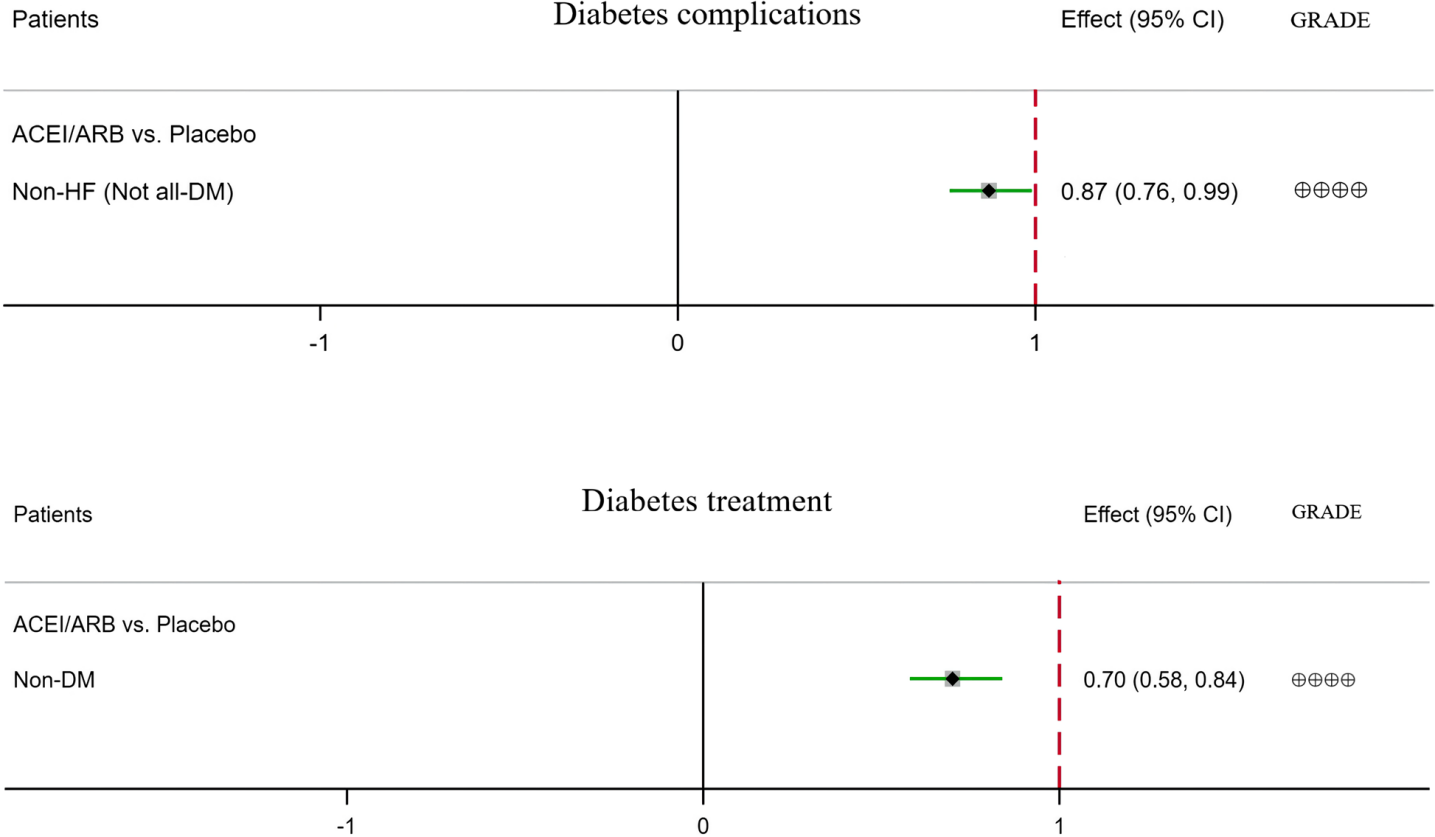
The effect of sacubitril/valsartan or ACEI/ARB on diabetes complications or diabetes treatment

The meta-analysis results and grades of the quality of the evidence are summarised in Table 2, in which the results with a statistical difference are shown in Figs. 1, 2, and 3.

**Table 2:** Results of the meta-analysis

	Sacubitril/valsartan vs ACEI/ARB					ACEI/ARB vs placebo					Sacubitril/valsartan vs (ACEI/ARB) vs placebo (indirect)			
Outcomes	№ of patients (RCTs)	Certainty (GRADE)	RR	95% CI	*p*	№ of patients (RCTs)	Certainty (GRADE)	RR	95% CI	*p*	№ of patients (RCTs)	Certainty (GRADE)	RR	95% CI
**New-onset DM**														
**All patients (non-DM)**	5830/5824 (9)	⊕⊕⊕moderate^b^	0.91	0.76–1.09	0.32	2312/2688 (13)	⊕⊕⊕moderate^a^	**0.85**	**0.77–0.93**	0.0007*	5116/5129 (8) vs 2312/2688 (13)	⊕⊕⊕moderate^g^	**0.78**	**0.64–0.95**
Non-HF						7238/7347 (4)	⊕⊕low^a, b^	0.88	0.75–1.02	0.10				
Not all-HF	126/124 (1)	⊕very low^b–e^	2.95	0.31–28.00	0.35	12,695/12,786 (6)	⊕⊕⊕⊕high	**0.87**	**0.82–0.93**	0.0001*	126/124 (1) vs 12,695/12,786 (6)	⊕⊕⊕moderate^b, d, f, g^	2.57	0.27–24.42
HF	5704/5700 (8)	⊕⊕⊕moderate^b^	0.90	0.75–1.08	0.27	4368/4351 (3)	⊕⊕low^a, b^	0.53	0.26–1.04	0.07	4990/5005 (7) vs 4368/4351 (3)	⊕⊕⊕⊕high^f, g^	**0.24**	**0.12–0.48**
HFrEF	3617/3619 (6)	⊕⊕⊕moderate^b^	0.90	0.72–1.13	0.37	1429/1410 (2)	⊕⊕low^a, b^	0.53	0.14–1.97	0.34	3617/3619 (6) vs 1429/1410 (2)	⊕⊕⊕moderate^b, f, g^	**0.24**	**0.12–0.50**
HFpEF	2087/2081 (2)	⊕⊕⊕moderate^b^	0.89	0.64–1.25	0.51	3578/3571 (2)	⊕⊕⊕moderate^d^	**0.60**	**0.44–0.83**	0.002*	2087/2081 (2) vs 3578/3571 (2)	⊕⊕low^d, g^	**0.54**	**0.34–0.85**
ACEI	3524/3534 (5)	⊕⊕⊕moderate^b^	0.91	0.73–1.14	0.41	10,253/10,350 (6)	⊕⊕⊕moderate^a^	**0.79**	**0.64–0.99**	0.04*				
ARB	1592/1595 (3)	⊕⊕⊕moderate^b^	0.90	0.65–1.24	0.51	14,048/14,134 (7)	⊕⊕⊕⊕high	**0.89**	**0.83–0.95**	0.0003*				
**Hypoglycaemia**														
**All patients**	8739/8752 (6)	⊕⊕⊕moderate^d^	**1.85**	**1.12–3.06**	0.02*	16,524/16,519 (9)	⊕⊕⊕moderate^b^	1.06	0.94–1.20	0.33	7459/7468(5) vs 16,524/16,519 (9)	⊕⊕low^d, g^	**1.91**	**1.05–3.47**
Non-DM						2283/2316 (1)	⊕very low^b, e^	1.10	0.94–1.30	0.23				
Not all-DM	8739/8752 (6)	⊕⊕⊕moderate^d^	**1.85**	**1.12–3.06**	0.02*	9536/9532 (3)	⊕⊕⊕moderate^d^	**2.06**	**1.17–3.61**	0.01*	7459/7468(5) vs 9536/9532 (3)	⊕⊕⊕⊕high^d, f, g^	**5.71**	**2.02–16.21**
DM						4705/4671 (5)	⊕⊕⊕moderate^b^	0.90	0.73–1.10	0.29				
TIDM						1661/1661 (2)	⊕⊕⊕moderate^b, d^	0.82	0.52–1.30	0.41				
T2DM						1236/1241 (2)	⊕⊕⊕moderate^b^	0.89	0.69–1.14	0.35				
Non-HF						4762/4741 (2)	⊕⊕low^a, b, d^	1.26	0.85–1.87	0.25				
Not all-HF						9698/9716 (6)	⊕⊕⊕moderate^b^	1.02	0.90–1.16	0.76				
HF	8739/8752 (6)	⊕⊕⊕moderate^d^	**1.85**	**1.12–3.06**	0.02*									
HFrEF	5040/5066 (4)	⊕⊕⊕moderate^b, d^	1.18	0.62–2.26	0.61									
HFpEF	3699/3686 (2)	⊕⊕⊕moderate^d^	**3.59**	**1.51–8.55**	0.004*	2064/2062 (1)	⊕very low^b, d, e^	2.00	0.86–4.66	0.11	3699/3686 (2) vs 2064/2062 (1)	⊕⊕⊕⊕high^d, f, g^	**7.06**	**2.10–23.76**
ACEI	4873/4898 (3)	⊕⊕⊕moderate^b, d^	1.26	0.65–2.46	0.49	1808/1769 (1)	⊕very low^b, d, e^	1.01	0.63–1.60	0.98				
ARB	2586/2570 (2)	⊕⊕low^a, d^	**2.72**	**1.18–6.27**	0.02*	14,716/14,750 (8)	⊕⊕⊕moderate^b^	1.07	0.94–1.21	0.31				
**Elevated glycaemia**														
**All patients**	9607/9155 (7)	⊕⊕⊕moderate^b, d^	0.81	0.52–1.26	0.35	14,745/14,765 (9)	⊕⊕⊕moderate^b, d^	0.89	0.66–1.21	0.46	8327/7871 (6) vs 14,745/14,765 (9)	⊕⊕low^b, d, g^	0.61	0.34–1.09
Non-DM						2283/2316 (1)	⊕very low^b, d, e^	1.01	0.06–16.21	0.99				
Not all-DM	9607/9155 (7)	⊕⊕⊕moderate^b, d^	0.81	0.52–1.26	0.35	9565/9547 (4)	⊕⊕⊕moderate^b, d^	0.90	0.62–1.32	0.60	8327/7871 (6) vs 9565/9547 (4)	⊕⊕low^b, d, g^	0.59	0.31–1.11
DM						2897/2902 (4)	⊕⊕⊕moderate^b, d^	0.87	0.52–1.46	0.60				
TIDM						1661/1661 (2)	⊕⊕⊕moderate^b, d^	0.65	0.30–1.38	0.26				
T2DM						1236/1241 (2)	⊕⊕⊕moderate^b, d^	1.16	0.55–2.42	0.70				
Non-HF						2983/2987 ()	⊕⊕⊕moderate^b, d^	0.87	0.58–1.31	0.51				
Not all-HF	950/484 (1)	⊕very low^b, d, e^	0.59	0.28–1.23	0.16	9698/9716 (6)	⊕⊕⊕moderate^b, d^	0.91	0.55–1.50	0.70	950/484 (1) vs 9698/9716 (6)	⊕⊕low^b, d, g^	0.53	0.22–1.30
HF	8657/8671 (6)	⊕⊕⊕moderate^b, d^	0.96	0.56–1.66	0.88									
HFrEF	4809/4833 (3)	⊕⊕⊕moderate^b, d^	1.84	0.68–4.97	0.23									
HFpEF	3848/3838 (3)	⊕⊕⊕moderate^b, d^	0.70	0.35–1.38	0.30	2064/2062 (1)	⊕very low^b, d, e^	1.00	0.29–3.45	1.00	2568/2554 (2) vs 2064/2062 (1)	⊕⊕low^b, d, g^	0.71	0.17–2.97
ACEI	4642/4665 (2)	⊕⊕⊕moderate^b, d^	1.81	0.61–5.39	0.29									
ARB	3685/3206 (4)	⊕⊕⊕moderate^b, d^	0.70	0.43–1.14	0.15	14,745/14,765 (9)	⊕very low^b, d, e^	0.89	0.66–1.21	0.46				
**DM inadequate control**														
**All patients**	4661/4691 (3)	⊕⊕⊕moderate^b, d^	0.73	0.29–1.82	0.50	12,433/12,434 (7)	⊕⊕⊕moderate^b, d^	0.82	0.58–1.16	0.26	4661/4691 (3) vs 12,433/12,434 (7)	⊕⊕⊕moderate^b, d, f, g^	0.29	0.01–7.15
Not all-DM	4661/4691 (3)	⊕⊕⊕moderate^b, d^	0.73	0.29–1.82	0.50	9536/9532 (3)	⊕⊕⊕moderate^b, d^	0.65	0.22–1.89	0.43	4661/4691 (3) vs 9536/9532 (3)	⊕⊕⊕moderate^b, d, f, g^	0.22	0.01–6.37
DM						2897/2902 (4)	⊕⊕⊕moderate^b, d^	0.85	0.59–1.22	0.37				
TIDM						1661/1661 (2)	⊕⊕low^a, b, d^	0.86	0.40–1.85	0.69				
T2DM						1236/1241 (2)	⊕⊕⊕moderate^b, d^	0.84	0.56–1.28	0.42				
Non-HF						2954/2972(1)	⊕very low^b, d, e^	0.34	0.03–3.22	0.34				
Not all-HF						7415/7400 (5)	⊕⊕⊕moderate^b, d^	0.87	0.60–1.24	0.43				
HF	4661/4691 (3)	⊕⊕⊕moderate^b, d^	0.73	0.29–1.82	0.50									
HFrEF	4512/4539 (2)	⊕⊕⊕moderate^b, d^	0.79	0.31–2.07	0.64									
HFpEF	149/152 (1)	⊕very low^b–e^	0.34	0.01–8.28	0.51	2064/2062 (1)	⊕very low^b, d, e^	0.60	0.14–2.50	0.48	149/152 (1) vs 2064/2062 (1)	⊕⊕⊕moderate^b–d, f, g^	0.20	0.01–6.74
ACEI	4512/4539 (2)	⊕very low^b, d, e^	0.79	0.31–2.07	0.64									
ARB	149/152 (1)	⊕very low^b–e^	0.34	0.01–8.28	0.51	12,433/12,434 (7)	⊕⊕⊕moderate^b, d^	0.82	0.58–1.16	0.26				
**Diabetes complications**														
**All patients**	8069/8083 (4)	⊕⊕⊕moderate^b, d^	0.80	0.49–1.32	0.38	19,635/19,540 (10)	⊕⊕⊕moderate^b^	0.90	0.80–1.01	0.08	6789/6799 (3) vs 19,635/19,540 (10)	⊕⊕low^b, d, g^	0.74	0.44–1.25
Non-DM						2283/2316 (1)	⊕very low^b, d, e^	0.34	0.01–8.30	0.51				
Not all-DM	8069/8083 (4)	⊕⊕⊕moderate^b, d^	0.80	0.49–1.32	0.38	14,455/14,322 (5)	⊕⊕⊕⊕high	**0.87**	**0.76–0.99**	0.04*	6789/6799 (3) vs 14,455/14,322 (5)	⊕⊕low^b, d, g^	0.73	0.43–1.24
DM						2897/2902 (4)	⊕⊕⊕moderate^b, d^	1.05	0.78–1.41	0.93				
TIDM						1661/1661 (2)	⊕⊕low^a, b, d^	1.05	0.68–1.61	0.82				
T2DM						1236/1241 (2)	⊕⊕⊕moderate^b, d^	1.05	0.70–1.57	0.81				
Non-HF						7873/7762 (3)	⊕⊕⊕⊕high	**0.87**	**0.76–0.99**	0.04*				
Not all-HF						9698/9716 (6)	⊕⊕⊕moderate^b, d^	1.03	0.77–1.37	0.87				
HF	8069/8083 (4)	⊕⊕⊕moderate^b, d^	0.80	0.49–1.32	0.38									
HFrEF	4370/4397 (2)	⊕⊕⊕moderate^b, d^	0.91	0.49–1.69	0.77									
HFpEF	3699/3686 (2)	⊕⊕⊕moderate^b, d^	0.64	0.28–1.47	0.29	2064/2062 (1)	⊕very low^b, d, e^	1.00	0.35–2.84	1.00	2419/2402 (1) vs 2064/2062 (1)	⊕⊕low^b, d, g^	0.66	0.17–2.62
ACEI	4203/4229 (1)	⊕very low^b, d, e^	0.95	0.50–1.81	0.88	4645/4652 (1)	⊕⊕low^e^	**0.85**	**0.73–0.98**	0.03*				
ARB	2586/2570 (2)	⊕⊕⊕moderate^b, d^	0.64	0.28–1.47	0.29	14,990/14,888 (9)	⊕⊕⊕moderate^b, d^	1.00	0.82–1.23	1.00				
**Diabetes treatment**														
Non-DM	2250/2375 (1)	⊕very low^b, d^	1.17	0.82–1.66	0.39	5552/5604 (2)	⊕⊕⊕⊕high	**0.70**	**0.58–0.84**	0.0002*	2250/2375 (1) vs 5552/5604 (2)	⊕⊕low^b, g^	0.68	0.43–1.10

*p < 0.05; *DM* diabetes mellitus, *HF* heart failure, *HFpEF* heart failure with preserved ejection fraction, *HFrEF* heart failure with reduced ejection fraction, *CI* confidence interval, *RR* relative risk. ^a^High initial heterogeneity. Downgraded once for inconsistency. ^b^The confidence interval crosses the clinical decision threshold of recommended and non-recommended treatment. Downgraded once for imprecision. ^c^Insufficient data to provide comment on precision. The participant number in analyses was < 300, unlikely to meet optimal information size parameters. Downgraded once for imprecision. ^d^Insufficient events to provide comment on precision. Downgraded once for imprecision. ^e^With data from only one study. Inconsistency cannot be assessed. Downgraded twice for inconsistency. ^f^Large effect magnitude. RR < 0.5 or RR > 2, upgrade once, RR < 0.2 or RR > 5, upgrade twice. ^g^Circumstantial evidence. Downgraded once for indirectness. 2, 3, and 4 do not repeat the downgrade

#### Results of sacubitril/valsartan compared with ACEI/ARB

##### The effect of sacubitril/valsartan compared with ACEI/ARB on new-onset DM

The data in this part of the study were all derived from patients with non-DM at baseline. Between the two groups, there was no difference in reduction in the risk of new-onset DM among all patients (RR = 0.91, 95% CI: 0.76–1.09, *p* = 0.32) and patients with not all-HF (defined as part of the study population having HF at baseline) (RR = 2.95, 95% CI: 0.31–28.00, *p* = 0.35), HF (RR = 0.90, 95% CI: 0.75–1.08, *p* = 0.27), HFrEF (RR = 0.90, 95% CI: 0.72–1.13, *p* = 0.37), and HF with preserved ejection fraction (HFpEF) (RR = 0.89, 95% CI: 0.64–1.25, *p* = 0.51). There was also no difference in the risk of new-onset DM between patients treated with sacubitril/valsartan and ACEI (RR = 0.91, 95% CI: 0.73–1.14, *p* = 0.41) or ARB (RR = 0.90, 95% CI: 0.65–1.24, *p* = 0.51) (Additional file 4: Fig. S3).

##### The effect of sacubitril/valsartan compared with ACEI/ARB on hypoglycaemia

Compared with ACEI/ARB, sacubitril/valsartan treatment did significantly increase the risk of hypoglycaemia among patients with HF (/not all-DM [defined as part of the study population having DM at baseline]) (RR = 1.85, 95% CI: 1.12–3.06, *p* = 0.02) and HFpEF (RR = 3.59, 95% CI: 1.51–8.55, *p* = 0.004), as was the comparison of sacubitril/valsartan and ARB (RR = 2.72, 95% CI: 1.18–6.27, *p* = 0.02) treatment, but the increase in the risk of hypoglycaemia among patients with HFrEF (RR = 1.18, 95% CI: 0.62–2.26, *p* = 0.61) was no significant between-group difference, as was the comparison of sacubitril/valsartan and ACEI (RR = 1.26, 95% CI: 0.65–2.46, *p* = 0.49) treatment (Additional file 4: Fig. S4).

##### The effect of sacubitril/valsartan compared with ACEI/ARB on elevated glycaemia

Between the two groups, there was no difference in reduction in the risk of elevated glycaemia among all patients (RR = 0.81, 95% CI: 0.52–1.26, *p* = 0.35) and patients with no all-HF (RR = 0.59, 95% CI: 0.28–1.23, *p* = 0.16), HF (RR = 0.96, 95% CI: 0.56–1.66, *p* = 0.88), HFrEF (RR = 1.84, 95% CI: 0.68–4.97, *p* = 0.23), and HFpEF (RR = 0.70, 95% CI: 0.35–1.38, *p* = 0.30). There was also no difference in the risk of elevated glycaemia between patients treated with sacubitril/valsartan and ACEI (RR = 1.81, 95% CI: 0.61–5.39, *p* = 0.29) or ARB (RR = 0.70, 95% CI: 0.43–1.14, *p* = 0.15) (Additional file 4: Fig. S5).

##### The effect of sacubitril/valsartan compared with ACEI/ARB on DM inadequate control 

All data of DM inadequate control came from patients with HF at baseline. Compared with ACEI/ARB, sacubitril/valsartan treatment did not significantly reduce the risk of DM inadequate control among patients with HF (RR = 0.73, 95% CI: 0.29–1.82, *p* = 0.50), HFrEF (RR = 0.79, 95% CI: 0.31–2.07, *p* = 0.64), and HFpEF (RR = 0.34, 95% CI: 0.01–8.82, *p* = 0.51), as were the comparison of sacubitril/valsartan and ACEI (RR = 0.79, 95% CI: 0.31–2.07, *p* = 0.64) or ARB (RR = 0.34, 95% CI: 0.01–8.28, *p* = 0.51) treatment (Additional file 4: Fig. S6).

##### The effect of sacubitril/valsartan compared with ACEI/ARB on diabetic complications

All data of diabetic complications came from patients with HF at baseline. Compared with ACEI/ARB, sacubitril/valsartan treatment did not significantly reduce the risk of diabetic complications among patients with HF (RR = 0.80, 95% CI: 0.49–1.32, *p* = 0.38), HFrEF (RR = 0.91, 95% CI: 0.49–1.69, *p* = 0.77), and HFpEF (RR = 0.64, 95% CI: 0.28–1.47, *p* = 0.29), as were the comparison of sacubitril/valsartan and ACEI (RR = 0.95, 95% CI: 0.50–1.81, *p* = 0.88) or ARB (RR = 0.64, 95% CI: 0.28–1.47, *p* = 0.29) treatment (Additional file 4: Fig. S7).

##### The effect of sacubitril/valsartan compared with ACEI/ARB on diabetes treatment

Compared with ACEI/ARB, sacubitril/valsartan treatment did not significantly reduce the subsequent use of oral antihyperglycaemic or insulin treatment among patients with new-onset DM (RR = 1.17, 95% CI: 0.82–1.66, *p* = 0.39) (Additional file 4: Fig. S8).

#### Result of ACEI/ARB compared with placebo

##### The effect of ACEI/ARB compared with placebo on new-onset DM

The data in this part of the study were all derived from patients without DM at baseline. Between the two groups, there were significant differences in reduction in the risk of new-onset DM among all patients (RR = 0.85, 95% CI: 0.77–0.93, *p* = 0.0007) and patients with not all-HF (RR = 0.87, 95% CI: 0.82–0.93, *p *< 0.0001) and those with HFpEF (RR = 0.60, 95% CI: 0.44–0.83, *p* = 0.002), as were the comparison of ACEI (RR = 0.79, 95% CI: 0.64–0.99, *p* = 0.04) or ARB (RR = 0.89, 95% CI: 0.83–0.95, *p* = 0.0003) and placebo treatment, but the reductions in the risk of new-onset DM among patients with non-HF (RR = 0.88, 95% CI: 0.75–1.02, *p* = 0.10), HF (RR = 0.53, 95% CI: 0.26–1.04, *p* = 0.07), and HFrEF (RR = 0.53, 95% CI: 0.14–1.97, *p* = 0.34) were no significant between-group difference (Additional file 4: Fig. S9).

##### The effect of ACEI/ARB compared with placebo on hypoglycaemia

Compared with placebo, ACEI/ARB treatment did significantly increase in the risk of hypoglycaemia among patients with not all-DM (RR = 2.06, 95% CI: 1.172–3.61, *p* = 0.01), but the increase in the risk of hypoglycaemia among all patients (RR = 1.06, 95% CI: 0.94–1.20, *p* = 0.33), and patients with non-HF (RR = 1.26, 95% CI: 0.85–1.87, *p* = 0.25), not all-HF (RR = 1.02, 95% CI: 0.90–1.16, *p* = 0.76), HFpEF (RR = 2.00, 95% CI: 0.86–4.66, *p* = 0.11), non-DM (RR = 1.10, 95% CI: 0.94–1.30, *p* = 0.23), DM (RR = 0.90, 95% CI: 0.73–1.10, *p* = 0.29), type 1 DM (T1DM) (RR = 0.82, 95% CI: 0.52–1.30, *p* = 0.41), and type 2 DM (T2DM) (RR = 0.89, 95% CI: 0.69–1.14, *p* = 0.35) were no significant between-group difference, as were the comparison of ACEI (RR = 1.01, 95% CI: 0.63–1.60, *p* = 0.98) or ARB (RR = 1.07, 95% CI: 0.94–1.21, *p* = 0.31) and placebo treatment (Additional file 4: Fig. S10).

##### The effect of ACEI/ARB compared with placebo on elevated glycaemia

Between the two groups, there was no significant difference in reduction in the risk of elevated glycaemia among all patients (RR = 0.89, 95% CI: 0.66–1.21, *p* = 0.46) and patients with non-HF (RR = 0.87, 95% CI: 0.58–1.31, *p* = 0.51), not all-HF (RR = 0.91, 95% CI: 0.55–1.50, *p* = 0.70), HFpEF (RR = 1.00, 95% CI: 0.29–3.45, *p* = 1.00), non-DM (RR = 1.01, 95% CI: 0.06–16.21, *p* = 0.99), no all-DM (RR = 0.90, 95% CI: 0.62–1.32, *p* = 0.60), DM (RR = 0.87, 95% CI: 0.52–1.46, *p* = 0.60), T1DM (RR = 0.65, 95% CI: 0.30–1.38, *p* = 0.26), and T2DM (RR = 1.16, 95% CI: 0.55–2.42, *p* = 0.70). There was also no difference in the risk of elevated glycaemia between patients treated with ARB (RR = 0.89, 95% CI: 0.66–1.21, *p* = 0.46) and placebo (Additional file 4: Fig. S11).

##### The effect of ACEI/ARB compared with placebo on DM inadequate control 

Compared with placebo, ACEI/ARB treatment did not significantly reduce the risk of DM inadequate control among all patients (RR = 0.82, 95% CI: 0.58–1.16, *p* = 0.26), non-HF (RR = 0.34, 95% CI: 0.03–3.22, *p* = 0.34), not all-HF (RR = 0.87, 95% CI: 0.60–1.24, *p* = 0.43), HFpEF (RR = 0.60, 95% CI: 0.14–2.50, *p* = 0.48), not all-DM (RR = 0.65, 95% CI: 0.22–1.89, *p* = 0.43), DM (RR = 0.85, 95% CI: 0.59–1.22, *p* = 0.37), T1DM (RR = 0.86, 95% CI: 0.40–1.85, *p* = 0.69), and T2DM (RR = 0.84, 95% CI: 0.56–1.28, *p* = 0.42), as was the comparison of ARB (RR = 0.82, 95% CI: 0.58–1.16, *p* = 0.26) and placebo treatment (Additional file 4: Fig. S12).

##### The effect of ACEI/ARB compared with placebo on diabetes complications

Compared with placebo, ACEI/ARB treatment did significant reduce the risk of diabetes complications among patients with non-HF (not all-DM) (RR = 0.87, 95% CI: 0.76–0.99, *p* = 0.04), as was the comparison of ACEI (RR = 0.85, 95% CI: 0.73–0.98, *p* = 0.03) and placebo treatment, but there were no significant difference in reduction in the risk of diabetes complications among all patients (RR = 0.90, 95% CI: 0.80–1.01, *p* = 0.08), patients with not all-HF (RR = 1.03, 95% CI: 0.77–1.37, *p* = 0.87), HFpEF (RR = 1.00, 95% CI: 0.35–2.84, *p* = 1.00), non-DM (RR = 0.34, 95% CI: 0.01–8.30, *p* = 0.51), DM (RR = 1.05, 95% CI: 0.78–1.41, *p* = 0.93), T1DM (RR = 1.05, 95% CI: 0.68–1.61, *p* = 0.82), and T2DM (RR = 1.05, 95% CI: 0.70–1.57, *p* = 0.81), as was the comparison of ARB (RR = 1.00, 95% CI: 0.82–1.23, *p* = 1.00) and placebo treatment (Additional file 4: Fig. S13).

##### The effect of ACEI/ARB compared with placebo on diabetes treatment

Compared with placebo, ACEI/ARB treatment did significantly reduce the subsequent use of oral antihyperglycaemic treatment or insulin among patients with new-onset DM (RR = 0.70, 95% CI: 0.58–0.84, *p* = 0.0002) (Additional file 4: Fig. S14).

#### Result of sacubitril/valsartan compared with placebo

No suitable study on the direct comparison of sacubitril/valsartan and placebo groups was included. Therefore, network meta-analysis was performed on the sacubitril/valsartan and placebo groups using the ACEI or ARB group as an intermediate group (Additional file 2: Figs. S2-S7 for network diagram).

##### The effect of sacubitril/valsartan compared with placebo on new-onset DM

The data in this part of the study were all derived from patients without DM at baseline. Between the two groups, there were significant differences in reduction in the risk of new-onset DM among all patients (RR = 0.78, 95% CI: 0.64–0.95), patients with HF (RR = 0.24, 95% CI: 0.12–0.48), HFrEF (RR = 0.24, 95% CI: 0.12–0.50), and HFpEF (RR = 0.54, 95% CI: 0.34–0.85), but among patients with not all-HF (RR = 2.57, 95% CI: 0.27–24.42), there was no significant between-group difference (Additional file 4: Figs. S15-S19).

##### The effect of sacubitril/valsartan on hypoglycaemia compared with placebo 

Compared with placebo, treatment with sacubitril/valsartan significantly increased the risk of hypoglycaemia among all patients (RR = 1.91, 95% CI: 1.05–3.47), patients with not all-DM (RR = 5.71, 95% CI: 2.02–16.21), and those with HFpEF (RR = 7.06, 95% CI: 2.10–23.76) (Additional file 4: Figs. S20-S22).

##### The effect of sacubitril/valsartan compared with placebo on elevated glycaemia

Compared with placebo, sacubitril/valsartan treatment reduced the risk of elevated glycaemia among all patients (RR = 0.61, 95% CI: 0.34–1.09), patients with not all-DM (RR = 0.59, 95% CI: 0.31–1.11), not all-HF (RR = 0.53, 95% CI: 0.22–1.30), and HFpEF (RR = 0.71, 95% CI: 0.17–2.97), but there was no significant between-group difference (Additional file 4: Figs. S23-S26).

##### The effect of sacubitril/valsartan compared with placebo on DM inadequate control 

Compared with placebo, sacubitril/valsartan treatment reduced the risk of DM inadequate control among all patients (RR = 0.29, 95% CI: 0.01–7.15) and patients with not all-DM (RR = 0.22, 95% CI: 0.001–6.37) and HFpEF (RR = 0.20, 95% CI: 0.01–6.74), but there was no significant between-group difference (Additional file 4: Figs. S27-S29).

##### The effect of sacubitril/valsartan compared with placebo on diabetes complications

Compared with placebo, sacubitril/valsartan treatment reduced the risk of diabetes complications among all patients (RR = 0.74, 95% CI: 0.44–1.25) and patients with not all-DM (RR = 0.73, 95% CI: 0.43–1.24) and HFpEF (RR = 0.66, 95% CI: 0.17–2.62), but there was no significant between-group difference (Additional file 4: Figs. S30-S32).

##### The effect of sacubitril/valsartan compared with placebo on diabetes treatment

Compared with placebo, sacubitril/valsartan treatment reduced the subsequent use of oral antihyperglycaemic treatment or insulin among patients with new-onset DM (RR = 0.68, 95% CI: 0.43–1.10), but there was no significant between-group difference (Additional file 4: Fig. S33).

### Analysis of heterogeneity, publication bias, and sensitivity

#### Direct comparison

Only the heterogeneity of the studies on the effect of ACEI/ARB compared with placebo on new-onset diabetes was high (*I*^2^ = 55%) (Additional file 4: Fig. S9). Thus, regression analysis was performed on four variables, including the year of study publication, HF at baseline, whether the study was a multi-centre study, and the drug used in the intervention group. The results showed that a single-centre study was the reason for the heterogeneity of our study. When the variable was used in subgroup analysis, the study results did not change, and the heterogeneity of the two subgroups was low (Additional file 4: Fig. S9). Therefore, the results of our study were reliable.

Funnel plots were drawn for the studies that included more than nine trials. Only studies on the effect of ACEI/ARB versus placebo on new-onset diabetes or diabetes complications were used to make funnel plots (Additional file 2: Figs. S8-S9), which showed that there was no publication bias. Egger’s test was used to test for publication bias in studies that included more than four trials. All *p*-values were > 0.05, so no publication bias was found in the included studies.

Sensitivity analysis was performed using Stata, and the results showed that two studies could be highly sensitive (Additional file 2: Figs. S10-S20). After the one-by-one exclusion method, the two results were found to be stable and credible. Therefore, the sensitivity of all studies was low, and the results of the direct comparison were stable and credible.

#### Network meta-analysis

Publication bias detection was performed using the method described above. The funnel plots (Additional file 4: Figs. S21-S25) showed possible publication bias in the results of the effect of sacubitril/valsartan compared with placebo on elevated glycaemia among all patients or patients with not all-DM. Egger’s test was used to test for publication bias in the above two studies as well as in other studies that did not lend themselves to funnel plotting. All *p*-values were > 0.05, so no publication bias was found in the included studies. Since no direct comparison studies of sacubitril/valsartan and placebo were included in the network meta-analysis, inconsistency and loop-loop inconsistency tests were not required.

### Quality of the studies

The GRADE assessment indicated that the overall quality of the evidence was mostly moderate and high. Of the 22 outcomes that were statistically significant, eight had high-quality evidence, ten had moderate-quality evidence, and four had low-quality evidence. Especially, in the sacubitril/valsartan versus placebo comparison, the number of studies with high, moderate, and low-quality evidence for the seven outcomes with statistical differences was 3, 3, and 1, respectively. Indicating that the estimated meta-analysis effects were likely to be close to or similar to the actual effects.

## Discussion

The results of the HOPE study showed that ramipril was associated with a lower rate of newly diagnosed diabetes in high-risk populations [5, 37], as was valsartan in the Navigator study [36]. The CHARM study reported that candesartan appeared to prevent diabetes in patients with HF [7], while the results of three network meta-analysis [46–48] showed that ARB/ACEI were associated with the lowest rate of new-onset diabetes among patients treated with ACEI/ARB and placebo. However, in the DREAM study, a large prospective study specifically addressing the role of ACEI in diabetes, treatment with ramipril for 3 years did not significantly reduce the incidence of diabetes among people with impaired fasting glucose levels or impaired glucose tolerance [6]. In the DREAM study, ramipril significantly increased the rate of returning to normal blood glucose levels [6], and in the HOPE study [5, 37], ramipril treatment significantly reduced hemoglobinA1c (HbA1c) levels in the first and second years compared with placebo. However, at the study endpoint, the changes in HbA1c levels relative to baseline were the same in both groups. Ramipril also significantly reduced the rate of oral hypoglycaemic agent or insulin use in patients with diabetes [5, 37]. These study findings suggest that ACEI/ARB played a role in reducing new-onset diabetes as well as controlling blood glucose levels. However, the effects remain to be clarified.

A post hoc analysis of the PARADIGM-HF study [9] showed that sacubitril/valsartan significantly reduced HbA1c levels compared with enalapril in patients with combined HF with DM at baseline, with similar but not statistically significant effects in patients with combined HF with non-DM at baseline. Sacubitril/valsartan treatment significantly reduced initial insulin use and the proportion of patients using glucose-lowering drug treatment in patients with combined HF with DM at baseline. However, no similar differences were found in patients with new-onset diabetes. Sacubitril/valsartan treatment reduced the incidence of new-onset diabetes compared with enalapril, and hypoglycaemia (a lateral response to the glucose-lowering effect) occurred more often in diabetes patients treated with sacubitril/valsartan compared with enalapril (a lateral response to the glucose-lowering effect of sacubitril/valsartan). However, neither reached statistical significance. These results suggest that sacubitril/valsartan exerted better glycaemic control compared with ACEI, but the effect on new-onset diabetes compared with placebo remains unclear.

This study was conducted to explore the effect of sacubitril/valsartan on the development of DM according to differences in the incidence of new-onset DM, and the potential role of sacubitril/valsartan in glycaemic control reflected by the differences in the incidence of remaining outcomes (hypoglycaemia, elevated glycaemia, DM inadequate control, diabetic complications, and DM need treatment).

### Major results

#### Sacubitril/valsartan compared with ACEI/ARB or placebo treatment

Compared with placebo, sacubitril/valsartan treatment significantly reduced the risks of new-onset DM in patients without DM and patients with HF, HFrEF, and HFpEF by 22%, 76%, 76%, and 46%, respectively, and significantly increased the risks of hypoglycaemia among all patients, patients without DM, and patients with HFpEF by 91%, 471%, and 606%, respectively, but reduced the risks of hyperglycaemia, inadequate DM control, diabetes complications, and diabetes treatment by 39%, 71%, 26%, and 32%, respectively, with no statistical difference. The results were similar in the subgroups.

Besides, compared with ACEI/ARB, sacubitril/valsartan treatment increased the risks of hypoglycaemia among patients with not all-DM, HF, HFpEF, and the comparison with ARB treatment by 85%, 85%, and 172%, respectively, with statistically significant differences, but reduced the risks of new-onset DM, elevated glycaemia, DM inadequate control, and diabetes complications by 9%, 19%, 27%, and 20%, respectively, with no significant difference. The results were similar in the subgroups.

#### ACEI/ARB compared with placebo treatment

Compared with placebo, ACEI/ARB treatment did significantly reduce the risks of new-onset DM among patients without DM and patients with not all-HF, HFpEF, and the comparison with ACEI or ARB by 15%, 13%, 40%, 21%, and 11%, respectively, diabetes complications among patients with not all-DM (/non-HF), and the comparison with ACEI by 13% and 15%, respectively, diabetes treatment among patients without DM by 30%, and significantly increased the risk of hypoglycaemia among patients with not all-DM by 106%, but reduced the risks of elevated glycaemia, DM inadequate control, and diabetes complications by 11%, 18%, and 11%, respectively, with no statistical difference.

### Mechanisms

Previous trials showed that the use of renin-angiotensin-aldosterone system (RAAS) inhibitor treatment could induce hypoglycaemia, improve blood glucose levels, and reduce the incidence of DM [49–51]. The specific mechanism of these effects is not clear, but the possible mechanisms include [52] increasing insulin secretion by decreasing the hepatic clearance of insulin, attenuating the pernicious effect of angiotensin II on the pancreas (such as vasoconstriction, apoptosis, and β-cell death), improving pancreatic blood flow [53], improving insulin resistance by enhancing adipocyte differentiation, and reducing inflammation to improve DM-related metabolism [46, 54] by inhibiting angiotensin II.

No direct studies have investigated the mechanism by which sacubitril/valsartan affects glycaemia. As a combined inhibitor of RAAS and neprilysin, the main mechanism of sacubitril/valsartan’s effect on glycaemia may be by inhibiting neprilysin. Neprilysin can decompose a variety of vasoactive peptides, including bradykinin, glucagon, glucagon-like peptide-1, insulin-B chain, vasoactive intestinal peptide, and other substances that play certain roles in glycaemia regulation [13]. Sacubitril/valsartan can decrease blood glucose levels by increasing glucose-lowering active peptides, especially glucagon-like peptide-1 and active peptides, which improve insulin sensitivity or islet function (such as bradykinin and plasma dipeptidyl peptidase 4) by inhibiting neprilysin. In addition, relevant studies showed that lower plasma natriuretic peptide (NP) concentrations were associated with insulin resistance and DM [55–60] possibly because reductions in NP lead to metabolic disturbances [14], especially in adipose tissue and skeletal muscle [61], whereas higher NP concentrations appear to have protective effects by reducing the risk of DM. As the main mechanism of the effect of sacubitril/valsartan in HF is by inhibiting NP degradation, this may be one of the important reasons why it prevents and improves DM. NP can improve glucose metabolism and insulin resistance by consuming excessive energy by increasing the oxidation of circulating free fatty acids [15], increasing the synthesis of adiponectin in adipocytes [62], inhibiting the secretion of pro-inflammatory cytokines by macrophages in adipose tissue [63], and promoting the transformation of white adipocytes into brown adipocytes [64, 65]. In addition, NP promotes beneficial metabolism by reducing hunger and ghrelin concentrations in circulating and increasing satiety in healthy individuals, which are beneficial for glycaemic control [66]. Finally, as sacubitril/valsartan can improve cardiac and renal function and thus affect the organs and tissues related to DM, these effects may explain why sacubitril/valsartan improves DM.

### Findings and thoughts

#### Sacubitril/valsartan

Our findings are similar to the post hoc analysis of the PARADIGM-HF study, which found that sacubitril/valsartan treatment significantly reduced the incidence of new-onset DM and increased hypoglycaemic events in patients with HF, suggesting a role for sacubitril/valsartan in controlling the development of diabetes and a possible role in lowering blood glucose levels. In addition, in the majority of cases, compared with ACEI/ARB or placebo, sacubitril/valsartan treatment reduced the incidence of new-onset DM, hyperglycaemia, inadequate DM control, diabetes complications, and diabetes treatment and increased the incidence of hypoglycaemia. These results also reflect the potential effectiveness of sacubitril/valsartan in treating diabetes in people with different co-morbidities, although statistical significance was not reached. Some additional findings were made in this study. Some differences in the effectiveness of treatment according to the study metrics, especially new-onset DM, hypoglycaemia, and hyperglycaemia, were seen in patients with different types of HF treated with sacubitril/valsartan. The biggest difference was in the risk of new-onset DM (HFpEF, RR 0.54 vs HFrEF, RR 0.24), hypoglycaemia (RR 3.59 vs RR 1.18), and hyperglycaemia (RR = 0.70 vs RR = 1.84). Sacubitril/valsartan treatment resulted in a higher proportion of hypoglycaemia in the HFpEF than in the HFrEF group (23/3699 vs 19/5040), and the control group data showed that ACEI/ARB treatment lowered the incidence of hypoglycaemia in the HFpEF group compared with the HFrEF group (6/3686 vs 16/5066), which suggested that the difference in the incidence of hypoglycaemia in different types of HF was not directly caused by the type of HF but by sacubitril/valsartan treatment. Furthermore, the proportion of inadequate DM control (0% vs 0.02%) and diabetes complications (0.02% vs 0.04%) was lower, and hypoglycaemia (0.06% vs 0.04%) was higher in the HFpEF than in HFrEF the group. Overall, sacubitril/valsartan treatment may be more effective in controlling glycaemia in patients with HFpEF than in patients with HFrEF. However, it should be noted that there was a lack of data comparing sacubitril/valsartan and placebo in patients with HFrEF.

#### ACEI/ARB

The effect of ACEI/ARB in preventing DM was similar to the results of previous large clinical studies and meta-analyses [5, 7, 36, 37, 46–48], i.e. ACEI/ARB treatment significantly reduced the incidence of new-onset diabetes. Furthermore, there were some additional findings in which ACEI treatment reduced the risk of new-onset DM among patients with not all-HF or with HFpEF, increased the risk of hypoglycaemia, and reduced diabetes complications among patients with not all-DM compared with placebo, with statistically significant differences. Also, ACEI/ARB treatment exerted positive effects on other research indicators. The above results suggested that ACEI/ARB also have a role in glycaemic control.

### Thoughts on therapeutic effects

The differences in the subgroup results may be due to the differential effects of sacubitril/valsartan, ACEI/ARB treatment in patients with various background diseases, such as with or without HF and different types of HF. Currently, no progress has been made in studies of effective ways to treat HF and thus improve DM [4]. Therefore, it is difficult to explain our findings by the indirect therapeutic effect of sacubitril/valsartan on HF and thus on DM. Considering that the underlying cardiovascular diseases in patients with HF can lead to other metabolic and energetic disturbances closely related to DM [4, 67], we hypothesise that sacubitril/valsartan may have a relevant beneficial effect on DM by directly ameliorating these adverse pathophysiological alterations and thus have a more pronounced effect in patients with HF. The pathophysiological heterogeneity within the broader clinical spectrum of HFpEF, which may represent different progression or disease, may be involved in the different effects of neurohormone antagonists in patients with HFrEF and HFpEF. In addition, diabetes has different cardiovascular effects in patients with different types of HF [68, 69], resulting in the additional effects of sacubitril/valsartan, ACEI/ARB on DM [70].

### Strengths and limitations

We conducted a reasonable search of the literature and carefully screened the results using strict standards, which resulted in a large study sample size. This was the first meta-analysis of the effect of sacubitril/valsartan and comprehensive, updated analysis of the role of ACEI/ARB in patients with diabetes, which included only RCTs. Most of the studies in this analysis were large multi-centre clinical trials and most of our analyses were derived from the analysis of moderate to high-quality evidence. Hence, the quality of our meta-analysis was high. Our study confirmed the effect of sacubitril/valsartan and comprehensively analysed the role of ACEI/ARB in DM. Sodium-glucose cotransporter 2 inhibitor has become the only anti-diabetes drug that can reduce HF events, and our study may set the stage for whether sacubitril/valsartan or angiotensin-receptor/enkephalinase inhibitors could be used as anti-HF agents for the treatment of diabetes. However, several possible deficiencies should also be noted. Firstly, the metrics we studied were not the main objective of most of the trials, and the lack of clarity in the definitions and measurement of the metrics in most cases may have resulted in the application of different criteria, as well as bias, in our results. Secondly, no standardised definitions were used for HF, which also may have led to some bias in the subgroup analysis. Thirdly, most trials did not match patients and select dosages based on diabetes status, while studies using sacubitril/valsartan were primarily in people with HF, and studies using placebo were primarily in people with not all-HF, and the observation period of the individual studies was short.

## Conclusions

The results of our study, especially in reducing glycaemia and new-onset DM, revealed that sacubitril/valsartan treatment had a positive effect on the control of glycaemia and the development of DM, and ACEI/ARB also had a beneficial effect but the effect was weaker than that of sacubitril/valsartan. The above effects varied across disease settings and the evidence may have been the strongest in patients with HF. Hence, sacubitril/valsartan has the potential to become an anti-HF drug for the treatment of diabetes. However, the combined use of sacubitril/valsartan, ACEI, or ARB and conventional doses of diabetes medication may increase the incidence of hypoglycaemia and requires further studies. Dose adjustments of insulin or other antihyperglycaemic agents may be needed, especially in patients with HF. In conclusion, the effect, exact mechanism, and population that may benefit from sacubitril/valsartan treatment in DM need to be clarified by further studies. However, these results will bring more information and inspiration to the prevention and treatment of DM.

## Supplementary Information

**Additional file 1.** The review method registered in PROSPERO.

**Additional file 2: Table S1-S4.** Search strategy in PubMed, Embase, Cochrane Central Register of Controlled Trials, and ClinicalTrials.gov. **Table S5.** Eligibility criteria of included studies. **Figure S1.** Flow chart of literature search and study selection. **Figure S2**-**S7.** Network charts in network meta-analyses. **Figure S8**-**S9.** Funnel charts in direct comparisons. **Figure S10**-**S20.** Sensitivity analysis charts in direct comparisons. **Figure S21-S25.** Funnel charts in indirect comparisons.

**Additional file 3.** PRISMA 2020 for Abstracts Checklist.

**Additional file 4: Figure S1.** Methodological quality graph. **Figure S2.** Methodological quality summary. **Figure S3-S33.** Forest maps for all meta-analyses.

## Data Availability

All the data used to generate this meta-analysis is publicly available, and the datasets used and analysed during the current study are available from the corresponding author on reasonable request.

## Abbreviations

ACEI: Angiotensin-converting enzyme inhibitors
ARB: Angiotensin-receptor blockers
RAAS: Renin-angiotensin-aldosterone system
CI: Confidence interval
DM: Diabetes mellitus
GRADE: Grading of Recommendations Assessment, Development, and Evaluation
HF: Heart failure
NP: Natriuretic peptide
PLA: Placebo
PRISMA: Preferred Reporting Items for Systematic Reviews and Meta-Analyses
RCTs: Randomised clinical trials
ROB 2: Revised Cochrane risk-of-bias tool for randomized trials
RR: Relative risk
SV: Sacubitril/valsartan
