# Supplementary material for: Effect of sacubitril/valsartan and ACEI/ARB on glycaemia and the development of diabetes: a systematic review and meta-analysis of randomised controlled trials
Source: BMC Med. 2022 Dec 17;20:487. doi: 10.1186/s12916-022-02682-w (PMC9758945; doi:10.1186/s12916-022-02682-w)
Supplement: Supplementary file 1 — Additional file 1. The review method registered in PROSPERO. [file 12916_2022_2682_MOESM1_ESM.pdf]

Effect of sacubitril/valsartan and ACEI/ARB on glycaemia and the development of diabetes: A systematic review and meta-analysis of randomised controlled trials

To enable PROSPERO to focus on COVID-19 submissions, this registration record has undergone basic automated checks for eligibility and is published exactly as submitted. PROSPERO has never provided peer review, and usual checking by the PROSPERO team does not endorse content. Therefore, automatically published records should be treated as any other PROSPERO registration. Further detail is provided here.

Review methods were amended after registration. Please see the revision notes and previous versions for detail.

Citation

Ruxin Wang, Haowen Ye, Yongting Zhao, Jinjing Wei, Ying Wang, Xiaofang Zhang, Lihong Wang. Effect of sacubitril/valsartan and ACEI/ARB on glycaemia and the development of diabetes: A systematic review and meta-analysis of randomised controlled trials. PROSPERO 2022 CRD42022336311 Available from: https://www.crd.york.ac.uk/prospero/display_record.php?ID=CRD42022336311

Review question [1 change]

Does sacubitril/valsartan or ACEI/ARB has effect on glycaemia and the development of diabetes?

Searches [1 change]

The Cochrane Library, Embase, PubMed and ClinicalTrials.gov; as of May 25, 2022; The publication date and language restrictions are not applied.

Types of study to be included [1 change]

Randomised controlled trials only.

Condition or domain being studied [1 change]

Diabetes mellitus (DM) is one of major public health problems in the world today. The latest global estimates from the International Diabetes Federation indicate that 537 million adults had DM in 2021, and that number is expected to increase to 643 million by 2030. DM often coexists with multiple diseases, especially in patients with heart failure (HF), in which the prevalence of diabetes is as high as 35 – 40%, and viceversa, leading to an adverse interactive effect on prognosis. The dual prevalence of DM and HF urgently requires effective treatments to address the increased burden in patients. Diabetes mellitus (DM) is one of major public health problems in the world today. The latest global estimates from the International Diabetes Federation indicate that 537 million adults had DM in 2021, and that number is expected to increase to 643 million by 2030. DM often coexists with multiple diseases, especially in patients with heart failure (HF), in which the prevalence of diabetes is as high as 35 – 40%, and viceversa, leading to an adverse interactive effect on prognosis. The dual prevalence of DM and HF urgently requires effective treatments to address the increased burden in patients.

Participants/population [1 change]

Inclusion: Adults.

Exclusion: Patients with contraindications to the study drugs and pregnant women.

Intervention(s), exposure(s) [1 change]

(1) In the comparison between sacubitril/valsartan and ACEI/ARB, the experimental group was intervened with sacubitril/valsartan; (2) In the comparison between ACEI/ARB and placebo, the experimental group was intervened with ACEI/ARB.

Comparator(s)/control [1 change]

(1) In the comparison between sacubitril/valsartan and ACEI/ARB, the control group was intervened with ACEI/ARB; (2) In the comparison between ACEI/ARB and placebo, the experimental group was intervened with placebo.

Main outcome(s) [1 change]

Number of adverse reactions related to DM listed in the results of trials, include new-onset DM, hypoglycaemia, elevated glycaemia, inadequate control DM, diabetes complications, and diabetes treatment, from baseline to the end of the trials.

Measures of effect

The 95% confidence interval (CI) and relative risk (RR) were used in the synthesis or presentation of the results. The Mantel-Haenszel method and the Z test were used to determine the overall results and the significance of the RR.

Additional outcome(s) [1 change]

None.

Measures of effect

None

Data extraction (selection and coding) [1 change]

Two reviewers independently extracted data from the RCTs that met the criteria and the guidelines in Cochrane Reviewer's Handbook, and all authors discussed the results in the event of discrepancies. The research data were retrieved from the original published manuscript or the results in ClinicalTrials.gov. The following data were extracted from each trial: 1. name of the trial, author, and registration number; 2. year of publication; 3. number of participants enrolled; 4. characteristics of the participants at baseline, including DM status, age, and gender; 5. the drug used in the control group; 6. study duration; 7. main outcomes; and 8. the number of participants with new-onset DM, hypoglycaemia, hyperglycaemia, inadequate DM control, diabetes treatment, and diabetes complications from baseline to the end of the study.

Risk of bias (quality) assessment [1 change]

Two researchers separately assessed the risk of bias for each qualified trial using the Cochrane Risk of Bias Tool for Randomised Trials (RoB 2) and compiled a risk of bias table as described in the Cochrane Handbook. We used the

GRADE principles to assess the quality of the evidence in this meta-analysis. The quality of the evidence was graded as very low, low, moderate, or high by measuring the risk of bias, inconsistency, indirectness, imprecision, and publication bias.

Strategy for data synthesis [1 change]

The data were analysed using Review Manager 5.4 and Stata 17.0. Direct comparisons of sacubitril/valsartan and ACEI/ARB groups and between ACEI/ARB and placebo groups were performed using Review Manager. A network meta-analysis of sacubitril/valsartan and placebo groups was performed using the ACEI or ARB group as an intermediate group and the "Network" program of Stata. Sensitivity analysis and publication bias detection were performed using Stata, and I^2 was used to assess heterogeneity. An I^2 value of ≥ 50% or a corresponding P-value of < 0.05 was considered to indicate heterogeneity among the studies. In that case, we used a random model and performed meta-regression and subgroup analysis. An I^2 of < 50% and a corresponding P-value of > 0.05 were considered to indicate no obvious heterogeneity in the results, and a fixed model was used. Due to the lack of direct comparison, there was no need to test for inconsistency in the network meta-analysis. The data were extracted from each trial and expressed as binary risk.

Analysis of subgroups or subsets [1 change]

Subgroup analysis was performed according to whether the patients had DM or HF (the included studies may have used different criteria for HF, and we did not use a standardised definition for HF) at baseline.

Contact details for further information

Ruxin Wang

wrxinxin@foxmail.com

Organisational affiliation of the review [1 change]

The First Affiliated Hospital of Jinan University

Review team members and their organisational affiliations [1 change]

Mr Ruxin Wang. The First Affiliated Hospital of Jinan University

Haowen Ye. The First Affiliated Hospital of Jinan University

Yongting Zhao. The Second Affiliated Hospital of Harbin Medical University

Jinjing Wei. The First Affiliated Hospital of Jinan University

Ying Wang. The First Affiliated Hospital of Jinan University

Xiaofang Zhang. The First Affiliated Hospital of Jinan University

Lihong Wang. The First Affiliated Hospital of Jinan University

Type and method of review [1 change]

Intervention, Meta-analysis, Network meta-analysis, Prevention, Systematic review

Anticipated or actual start date [2 changes]

25 May 2022

Anticipated completion date [2 changes]

10 December 2022

Funding sources/sponsors [1 change]

This study was supported by the Funding by Science and Technology Projects in Guangzhou (Grant Number: 202201020081), National Natural Science Foundation of China (Grant Number: 82200417), and Talent Introduction Funding Project of The First Affiliated Hospital of Jinan University (Grant Number: 808026).

Conflicts of interest

Language

English

Country

China

Stage of review [2 changes]

Review Ongoing

Subject index terms status

Subject indexing assigned by CRD

Subject index terms

Angiotensin Receptor Antagonists; Angiotensin-Converting Enzyme Inhibitors; Diabetes Mellitus; Humans; Randomized Controlled Trials as Topic; Valsartan; sacubitril

Date of registration in PROSPERO

10 June 2022

Date of first submission

30 May 2022

Stage of review at time of this submission [2 changes]

Preliminary searches	Yes	No
Piloting of the study selection process	Yes	No
Formal screening of search results against eligibility criteria	Yes	No
Data extraction	No	No
Risk of bias (quality) assessment	No	No
Data analysis	No	No

Revision note

We modified the title and language, changed the indirect comparison method from "indirect" program of STATA to network-meta analysis, modified the start and expected completion time, and added one author and two fundings.

The record owner confirms that the information they have supplied for this submission is accurate and complete and they understand that deliberate provision of inaccurate information or omission of data may be construed as scientific misconduct.

The record owner confirms that they will update the status of the review when it is completed and will add publication details in due course.

Versions

10 June 2022

10 June 2022

24 November 2022
