# Supplementary material for: Effect of sacubitril/valsartan and ACEI/ARB on glycaemia and the development of diabetes: a systematic review and meta-analysis of randomised controlled trials
Source: BMC Med. 2022 Dec 17;20:487. doi: 10.1186/s12916-022-02682-w (PMC9758945; doi:10.1186/s12916-022-02682-w)
Supplement: Supplementary file 2 — Additional file 2: Table S1-S4. Search strategy in PubMed, Embase, Cochrane Central Register of Controlled Trials, and ClinicalTrials.gov. Table S5. Eligibility criteria of included studies. Figure S1. Flow chart of literature search and study selection. Figure S2-S7. Network charts in network meta-analyses. Figure S8-S9. Funnel charts in direct comparisons. Figure S10-S20. Sensitivity analysis charts in direct comparisons. Figure S21-S25. Funnel charts in indirect comparisons. [file 12916_2022_2682_MOESM2_ESM.docx]

***Additional file 2***

1. **Supplemental tables**

**Table S1.** **Search strategy in PubMed.**

| **P**atient | N/A |
| --- | --- |
| **I**ntervention and  **C**ontrol | (("sacubitril and valsartan sodium hydrate drug combination" [Supplementary Concept]) OR (sacubitril valsartan[Title/Abstract])) OR ((((((("Angiotensin-Converting Enzyme Inhibitors"[Mesh]) OR (Angiotensin-Converting Enzyme Inhibitors[Title/Abstract])) OR (("Renin-Angiotensin System"[Mesh]) AND (inhibitor[Title/Abstract]))) OR (Angiotensin-Converting Enzyme Inhibitors[Title/Abstract])) OR (angiotensin-receptor blockers[Title/Abstract])) OR (Renin-Angiotensin System inhibitor[Title/Abstract])) AND (placebo[Title/Abstract])) |
| **O**utcome | N/A |
| Filter | Randomized Controlled Trial, Humans |

**Note:** N/A, not applicable.

**Table S2. Search strategy in** **Embase.**

| **P**atient | N/A |
| --- | --- |
| **I**ntervention and  **C**ontrol | #1. 'sacubitril plus valsartan'/exp OR 'sacubitril and valsartan':ti,ab  #2. 'angiotensin receptor antagonist'/exp OR 'dipeptidyl carboxypeptidase inhibitor'/exp  #3. 'renin angiotensin aldosterone system'/exp AND inhibitor:ti,ab  #4. 'angiotensin converting' AND enzyme AND inhibitors:ti,ab  #5. 'angiotensin receptor antagonist':ti,ab  #6. 'renin angiotensin' AND system AND inhibitor:ti,ab  #7. 'placebo':ti,ab  #8. #2 OR #3 OR #4 OR #5 OR #6  #9. #7 AND #8  #10. #1 OR #9 |
| **O**utcome | N/A |
| Filter | Randomized Controlled Trial, Humans, Embase, Preprints |

**Note:** N/A, not applicable.

**Table S3. Search strategy in** **Cochrane Central Register of Controlled Trials.**

| **P**atient | N/A |
| --- | --- |
| **I**ntervention and  **C**ontrol | #1 (sacubitril valsartan):ti,ab,kw OR (sacubitril valsartan sodium hydrate):ti,ab,kw OR (sacubitril valsartan drug combination):ti,ab,kw OR (LCZ 696):ti,ab,kw  #2 MeSH descriptor: [Angiotensin-Converting Enzyme Inhibitors] explode all trees  #3 MeSH descriptor: [Angiotensin Receptor Antagonists] explode all trees  #4 MeSH descriptor: [Renin-Angiotensin System] explode all trees  #5 (Angiotensin-Converting Enzyme Inhibitors):ti,ab,kw  #6 (angiotensin-receptor blockers):ti,ab,kw  #7 (inhibitor):ti,ab,kw  #8 (Renin-Angiotensin System inhibitor):ti,ab,kw  #9 (placebo):ti,ab,kw  #10 #4 and #7  #11 #2 or #3 or #5 or #6 or #8 or #10  #12 #9 and #11  #13 #1 or #12  #14 (randomised):ti,ab,kw OR (randomized):ti,ab,kw  #15 #13 and #14 |
| **O**utcome | N/A |
| Filter | ICTRP, CINAHL |

**Note:** N/A, not applicable.

**Table S4. Search strategy in** **ClinicalTrials.gov.**

| **P**atient | N/A |
| --- | --- |
| **I**ntervention and  **C**ontrol | (sacubitril/valsartan OR sacubitril valsartan sodium hydrate OR sacubitril valsartan drug combination OR LCZ696 OR LCZ 696 OR LCZ-696) OR ((ACEI OR ARB OR Renin-Angiotensin System inhibitor) AND (placebo)) |
| **O**utcome | N/A |
| Filter | Terminated, Completed, Unknown status, Adult (18–64), Older Adult (65+), Interventional (Clinical Trial), With Results |

**Note:** N/A, not applicable.

**Table S5:** Eligibility criteria of included studies.

|  | Inclusion criteria | Exclusion criteria |
| --- | --- | --- |
| Study design | Randomized controlled trials | Reviews or non-RCTs; cross-over study designs |
| Participants | Adults | Patients with contraindications to the study drugs; Pregnant women |
| Intervention and Comparators | Sacubitril/valsartan vs. ACEI/ARB/placebo; ACEI/ARB vs. placebo | Non-pharmaceutical treatment (e.g., exercise and apparatus) |
| Main outcomes | N/A | N/A |
| Included outcomes | New-onset DM, hypoglycaemia, elevated glycaemia, DM inadequate control, diabetic complications, DM treatment | Outcomes that cannot be specified |

**Note:** N/A, not applicable; DM, diabetes mellitus; ACEI, angiotensin-converting enzyme inhibitors; ARB, angiotensin-receptor blocker.

# Supplemental Figures


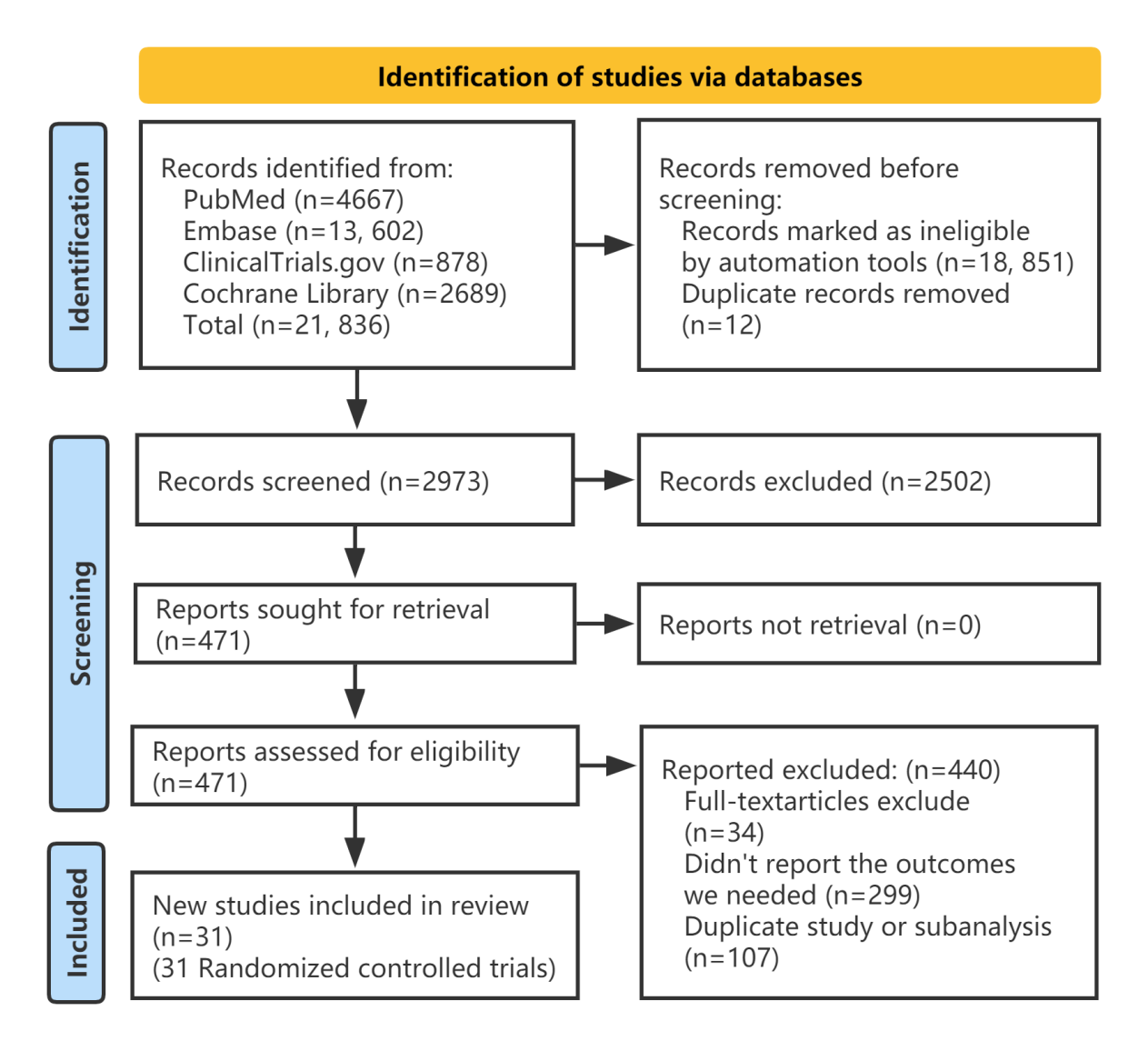
**Figure S1.** Flow chart of literature search and study selection.


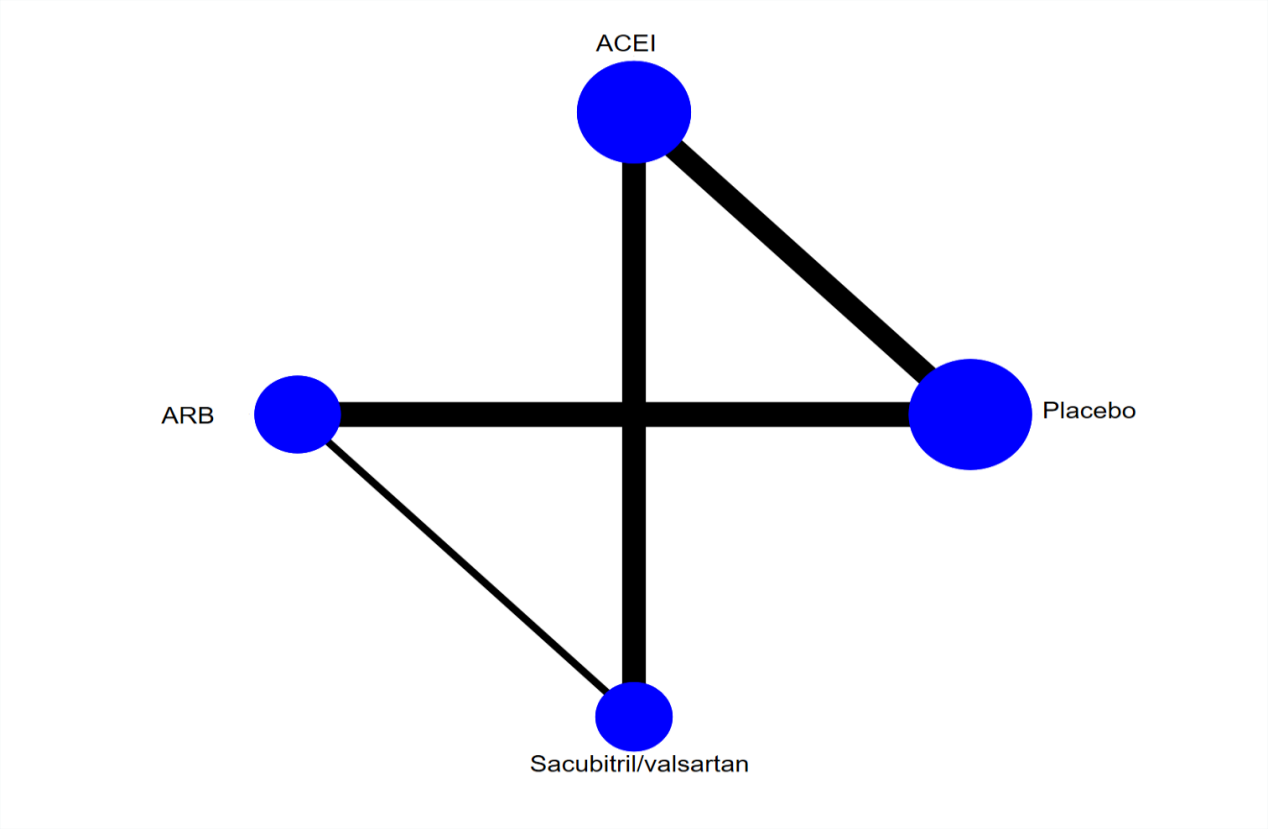


**Figure S2.** The effect of sacubitril/valsartan compared with placebo on new-onset DM among all patients (non-DM).


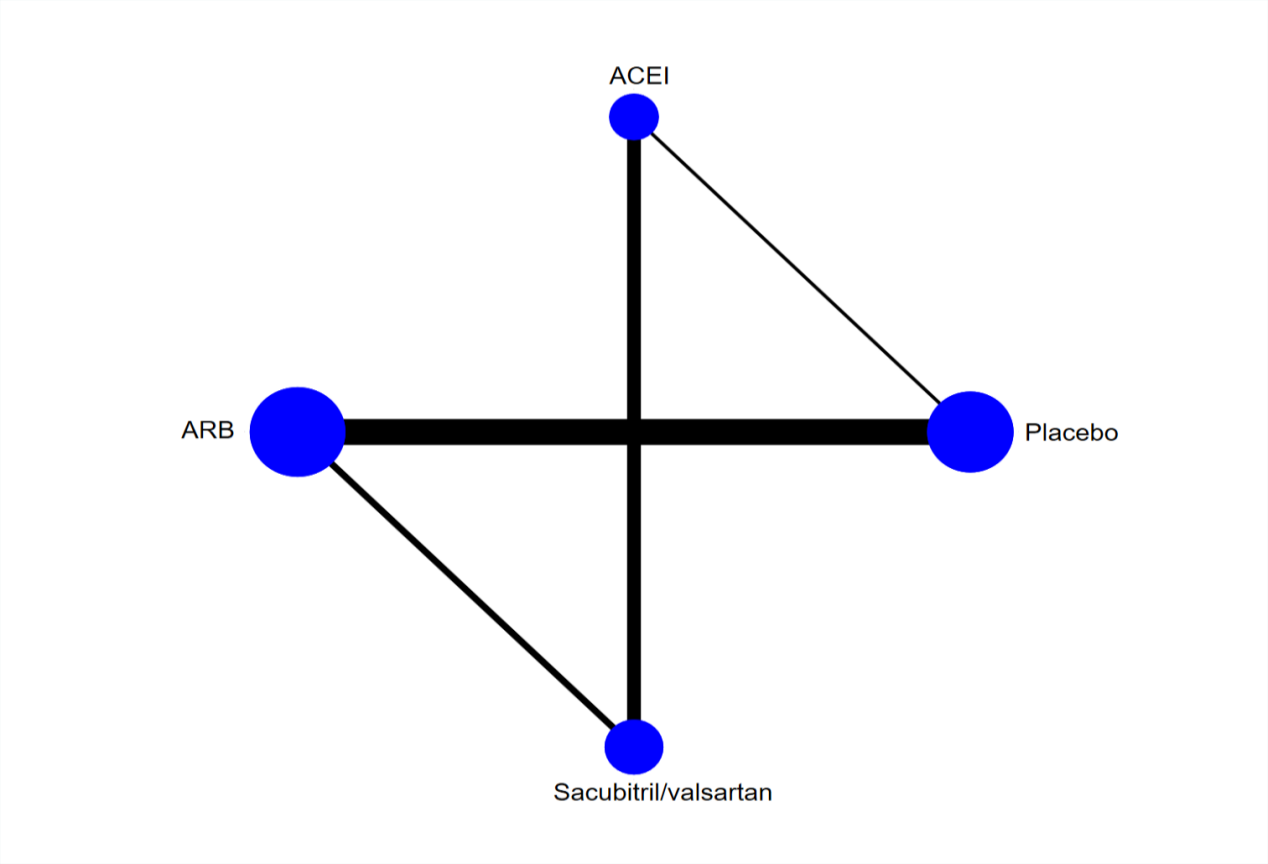
**Figure S3.** The effect of sacubitril/valsartan compared with placebo on hypoglycaemia among all patients.


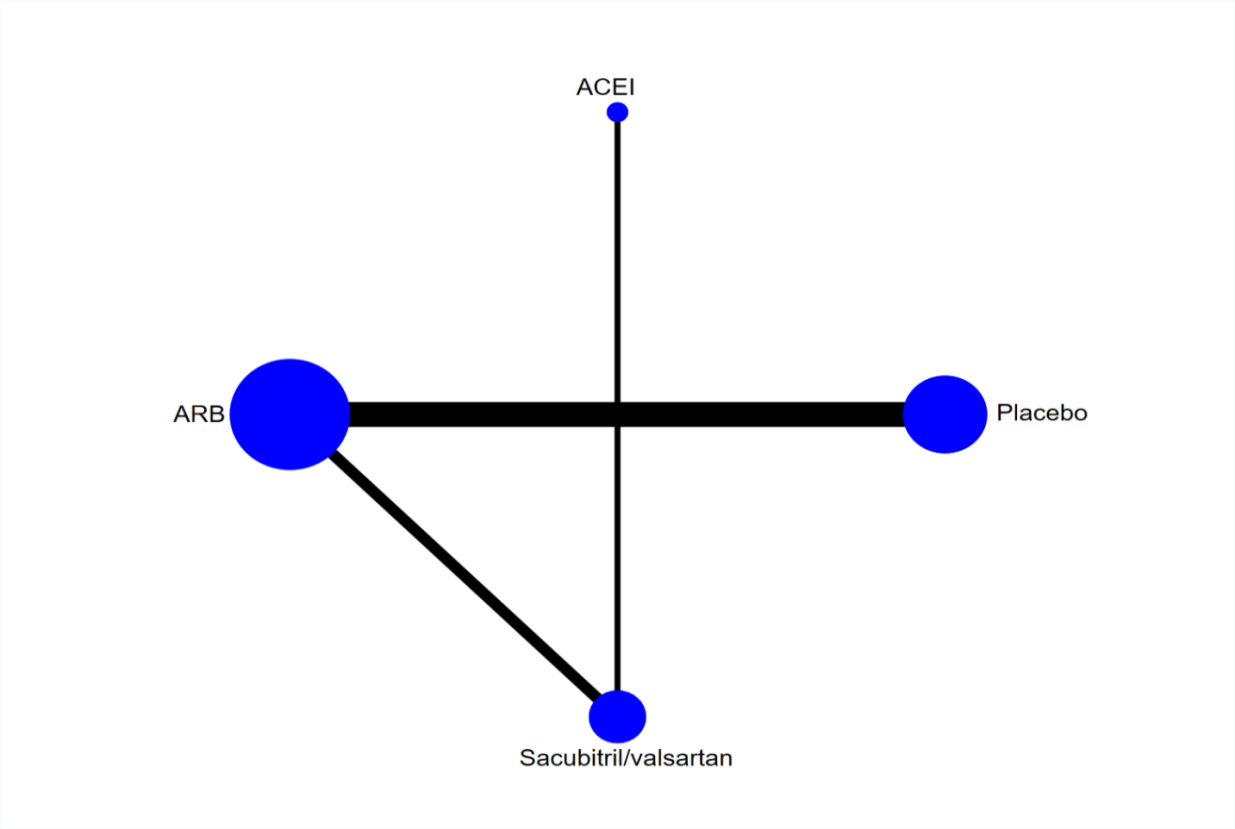
**Figure S4.** The effect of sacubitril/valsartan compared with placebo on elevated glycaemia among all patients.


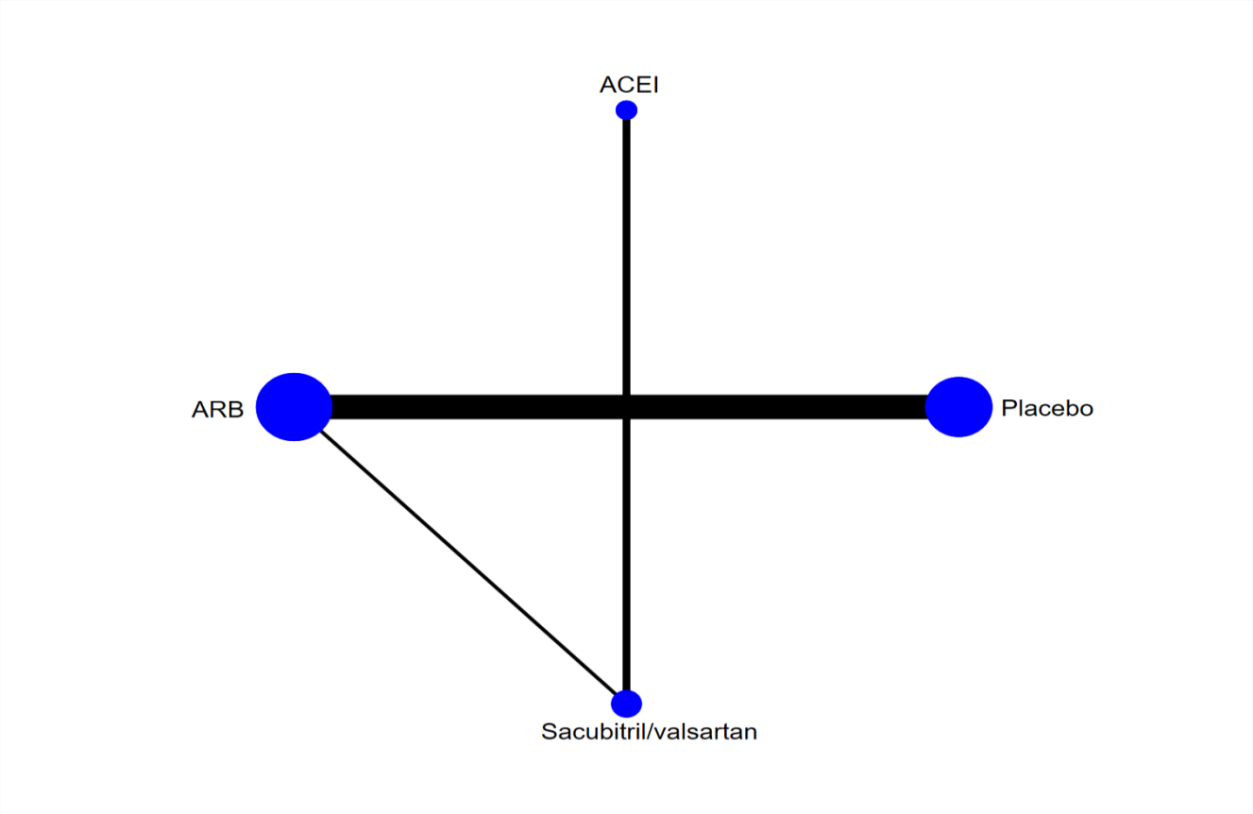
**Figure S5.** The effect of sacubitril/valsartan compared with placebo on DM inadequate control among all patients.


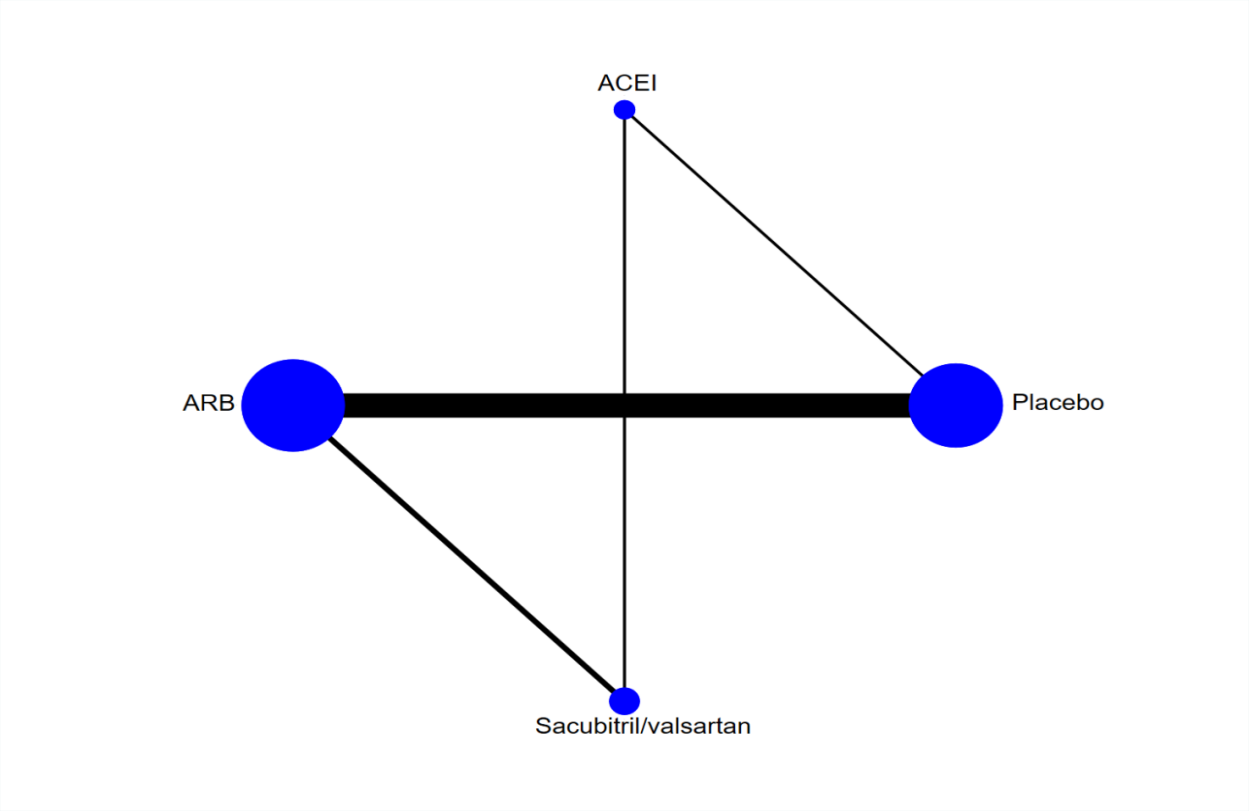
**Figure S6.** The effect of sacubitril/valsartan compared with placebo on diabetes complication among all patients.


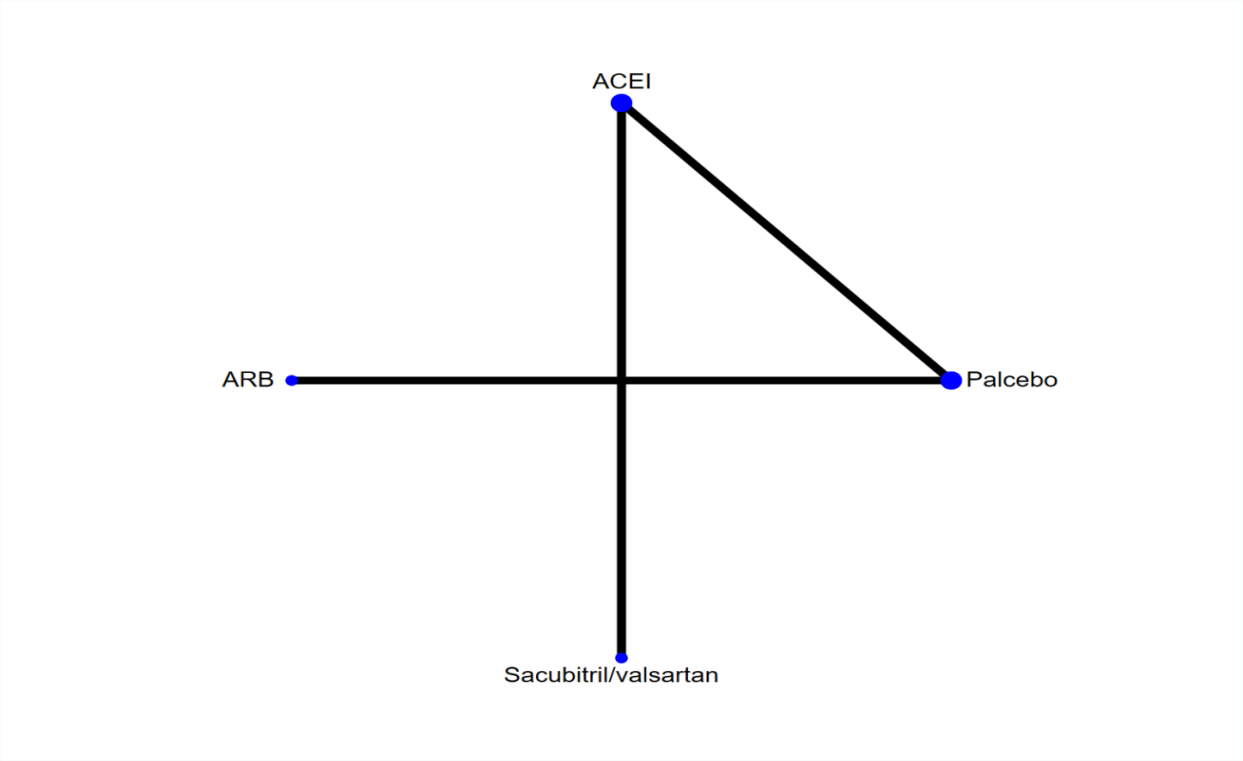
**Figure S7.** The effect of sacubitril/valsartan compared with placebo on diabetes treatment among all patients.

#

#
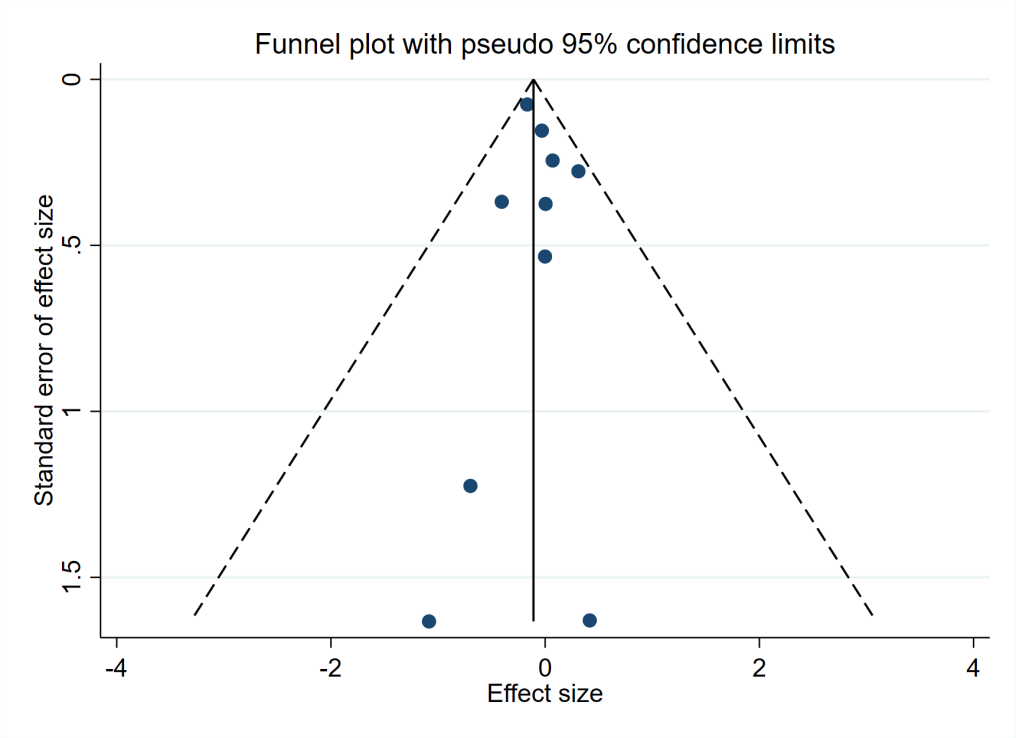
Figure S8. Funnel plot of the effect of ACEI/ARB compared with placebo on new-onset DM.


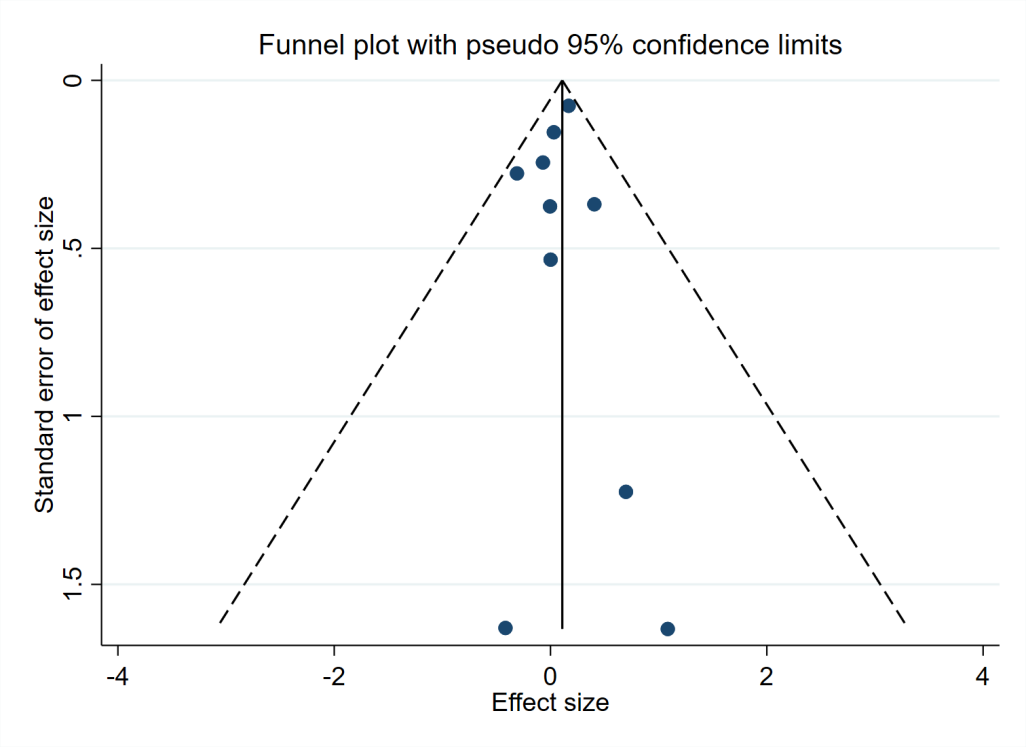
**Figure S9.** Funnel plot of the effect of ACEI/ARB compared with placebo on diabetes complications.

**
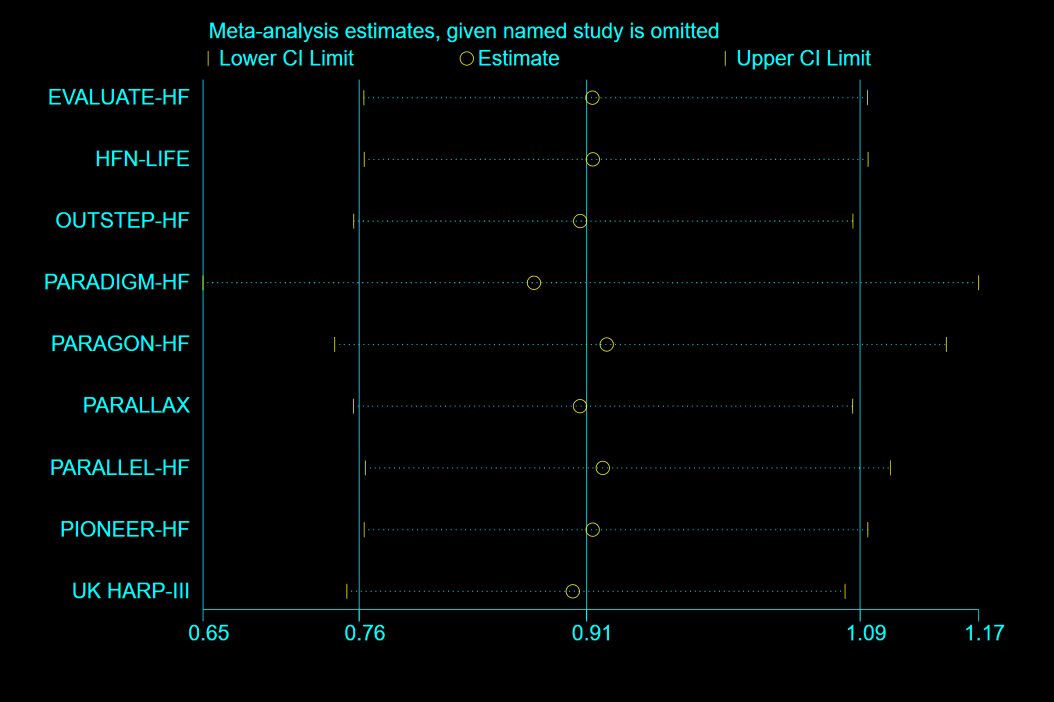
Figure S10.** Sensitivity analysis for the effect of sacubitril/valsartan compared with ACEI/ARB on new-onset DM.


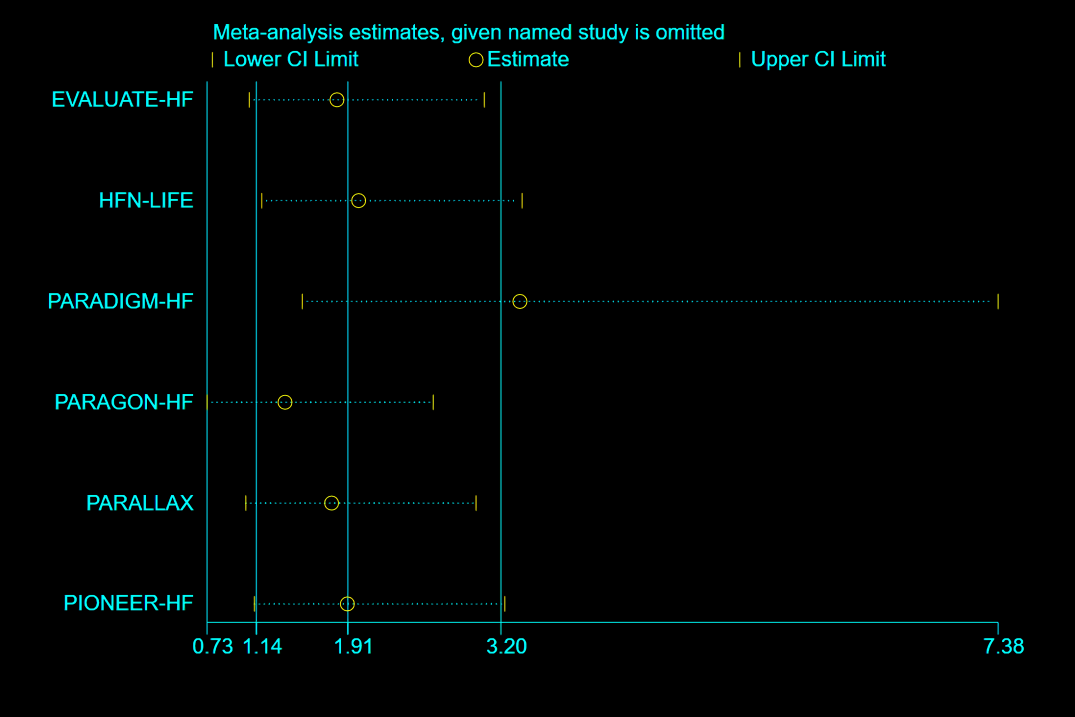
**Figure S11.** Sensitivity analysis for the effect of sacubitril/valsartan compared with ACEI/ARB on hypoglycaemia.


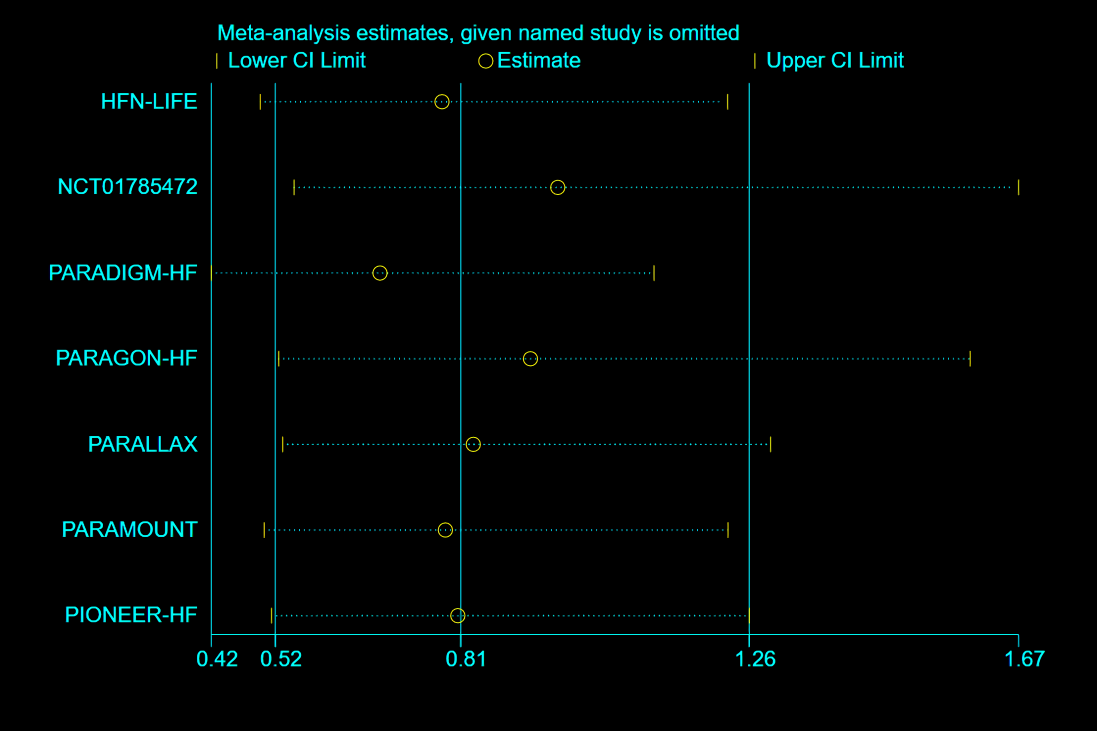
**Figure S12.** Sensitivity analysis for the effect of sacubitril/valsartan compared with ACEI/ARB on elevated glycaemia.

#
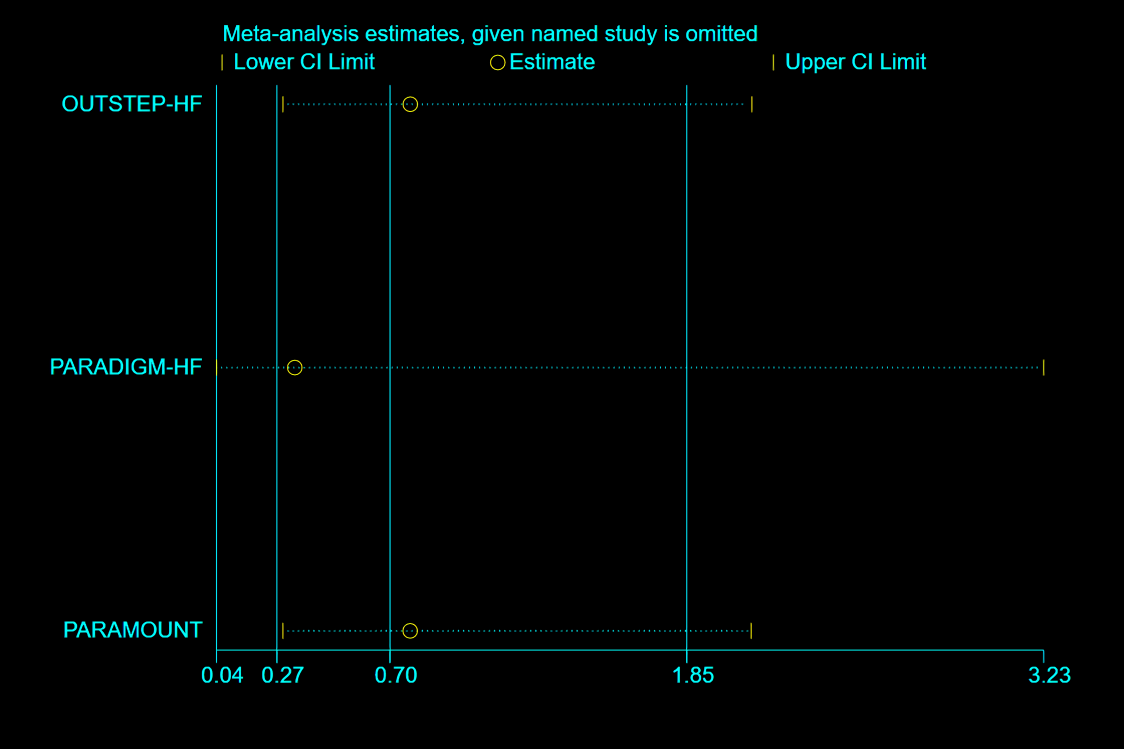
**Figure S13.** Sensitivity analysis for the effect of sacubitril/valsartan compared with ACEI/ARB on DM inadequate control.

#
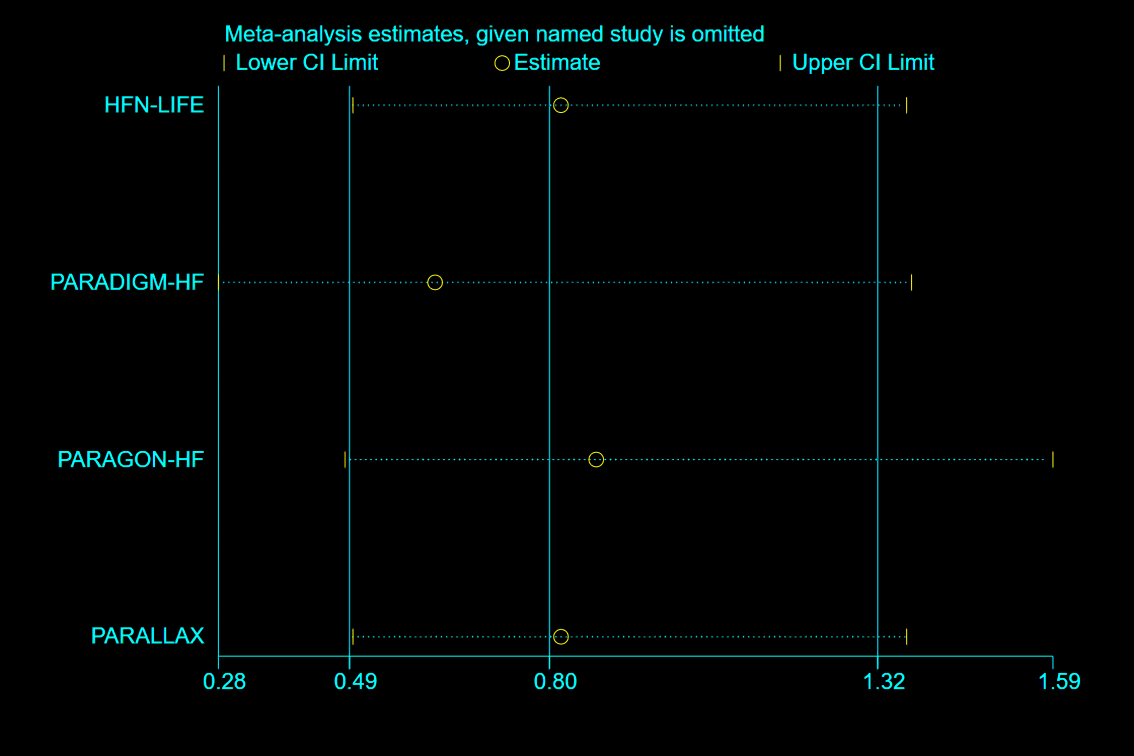
**Figure S14.** Sensitivity analysis for the effect of sacubitril/valsartan compared with ACEI/ARB on diabetic complications.


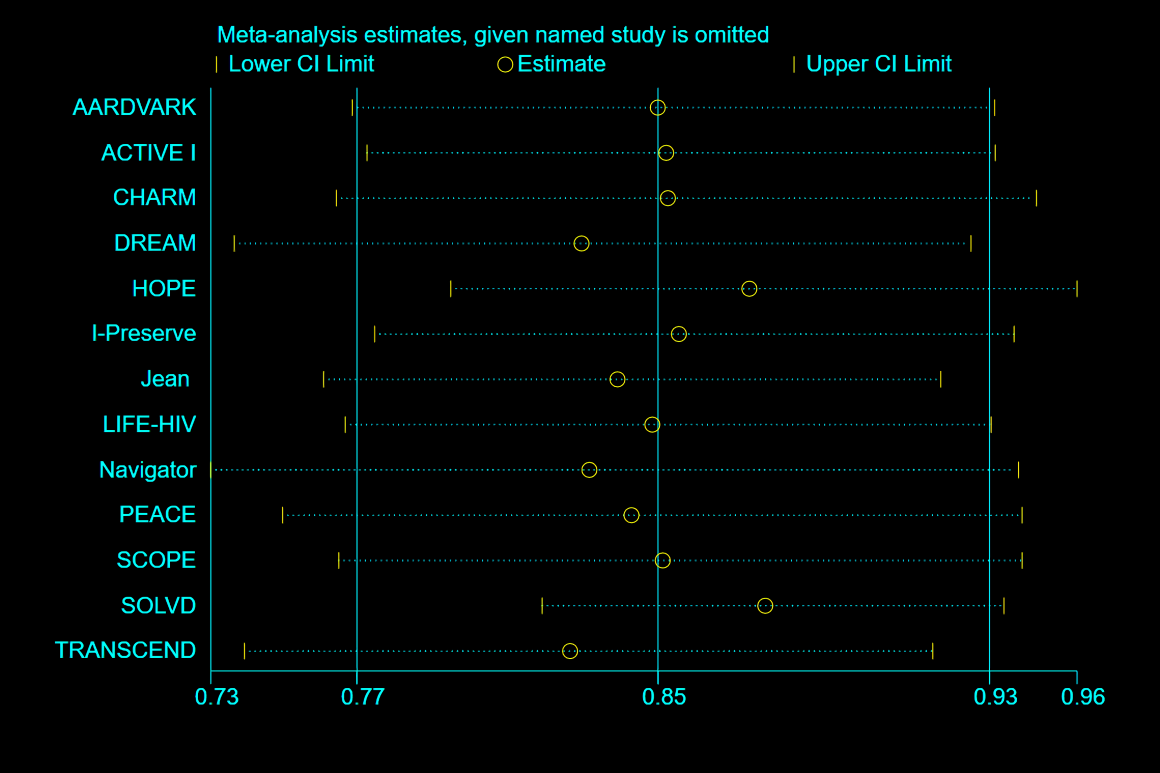
**Figure S15.** Sensitivity analysis for the effect of ACEI/ARB compared with placebo on new-onset DM.

**Figure S16.** Sensitivity analysis for the effect of ACEI/ARB compared with placebo on hypoglycaemia.

# **
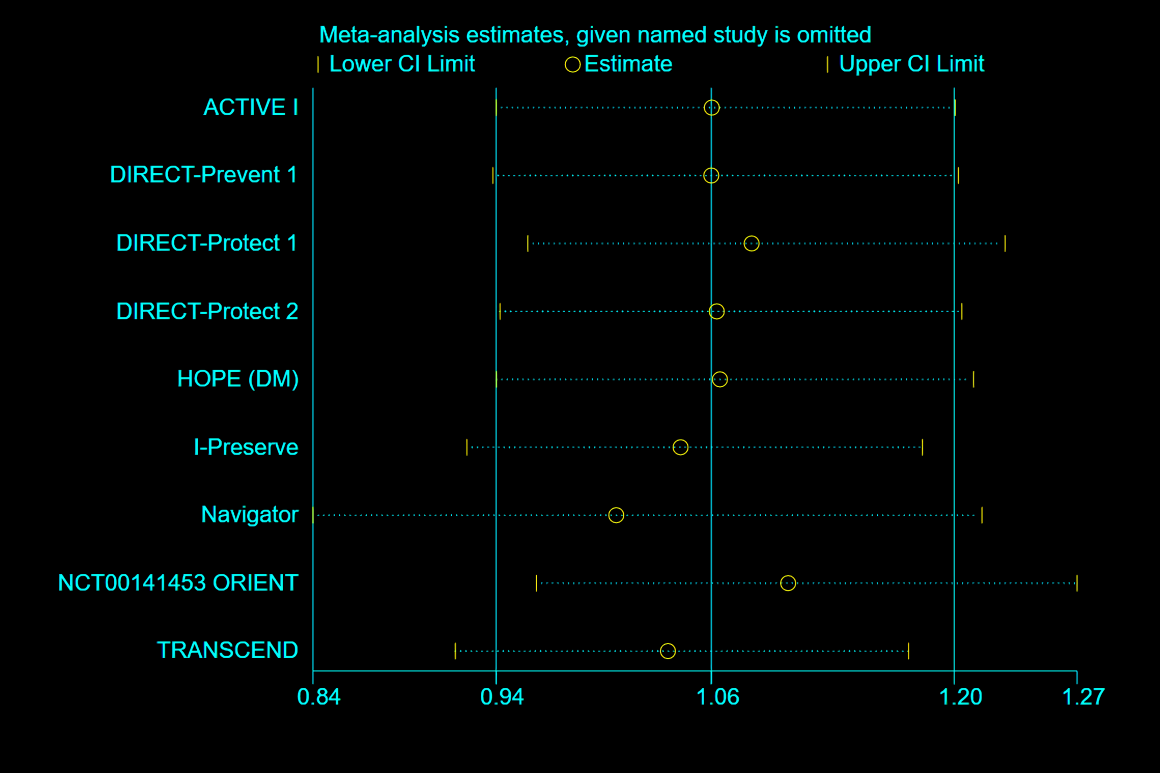

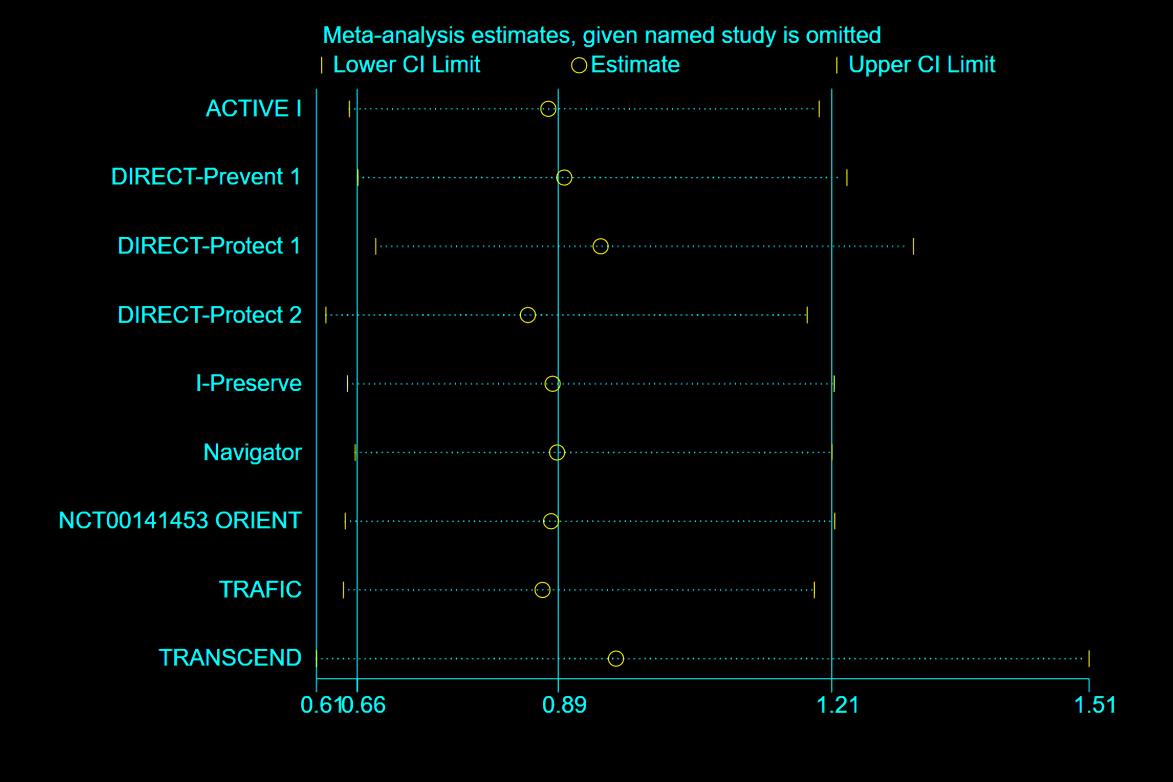
Figure S17.** Sensitivity analysis for the effect of ACEI/ARB compared with placebo on elevated glycaemia.

# **
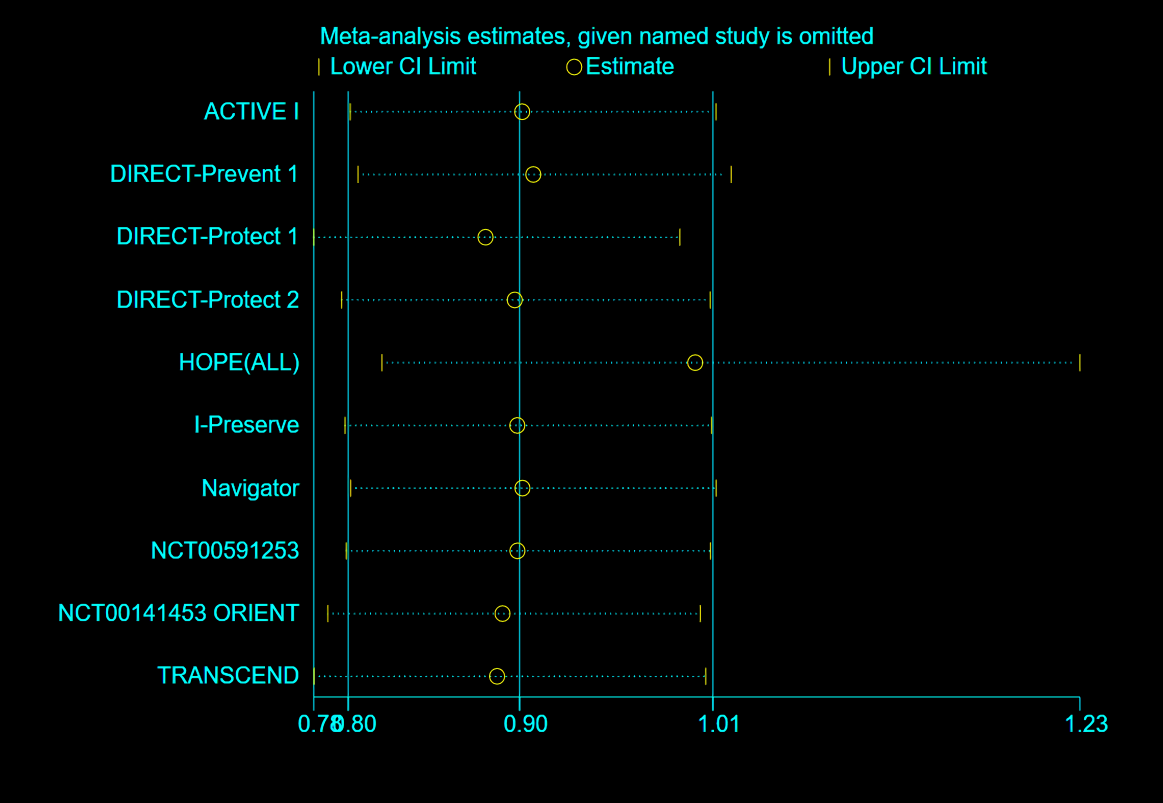
Figure S18.** Sensitivity analysis for the effect of ACEI/ARB compared with placebo on DM inadequate control.


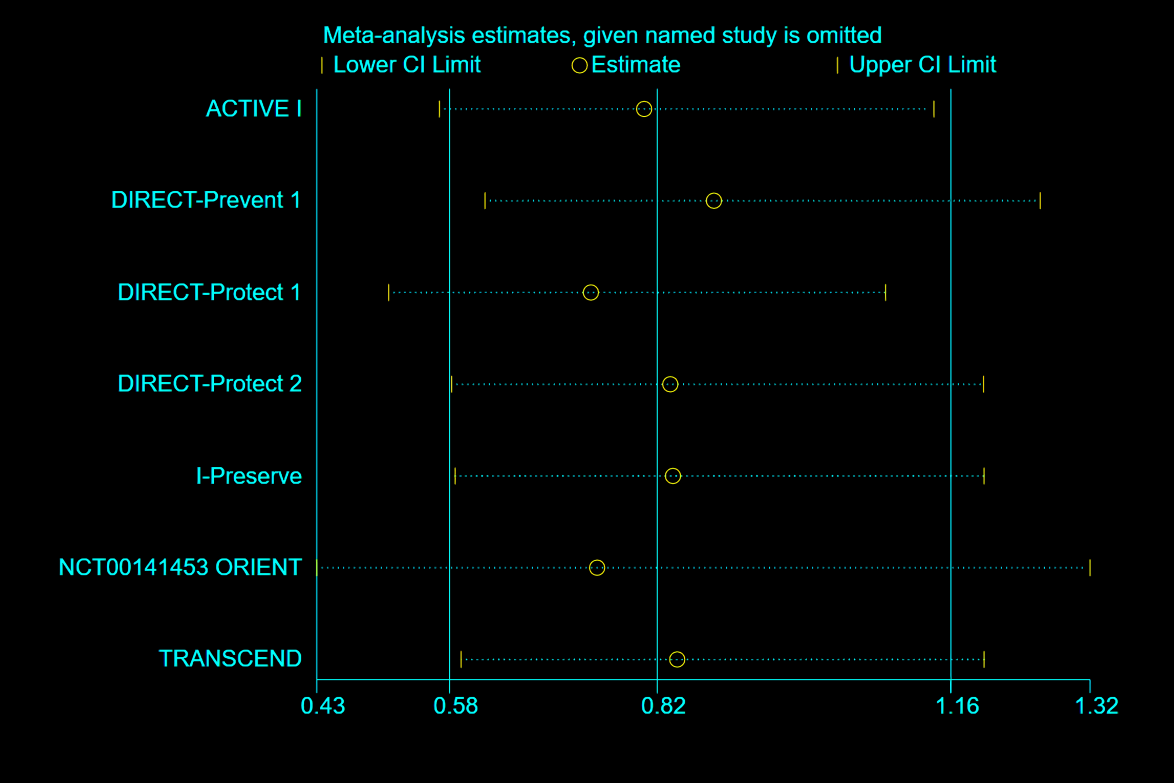
**Figure S19.** Sensitivity analysis for the effect of ACEI/ARB compared with placebo on diabetes complications

# **
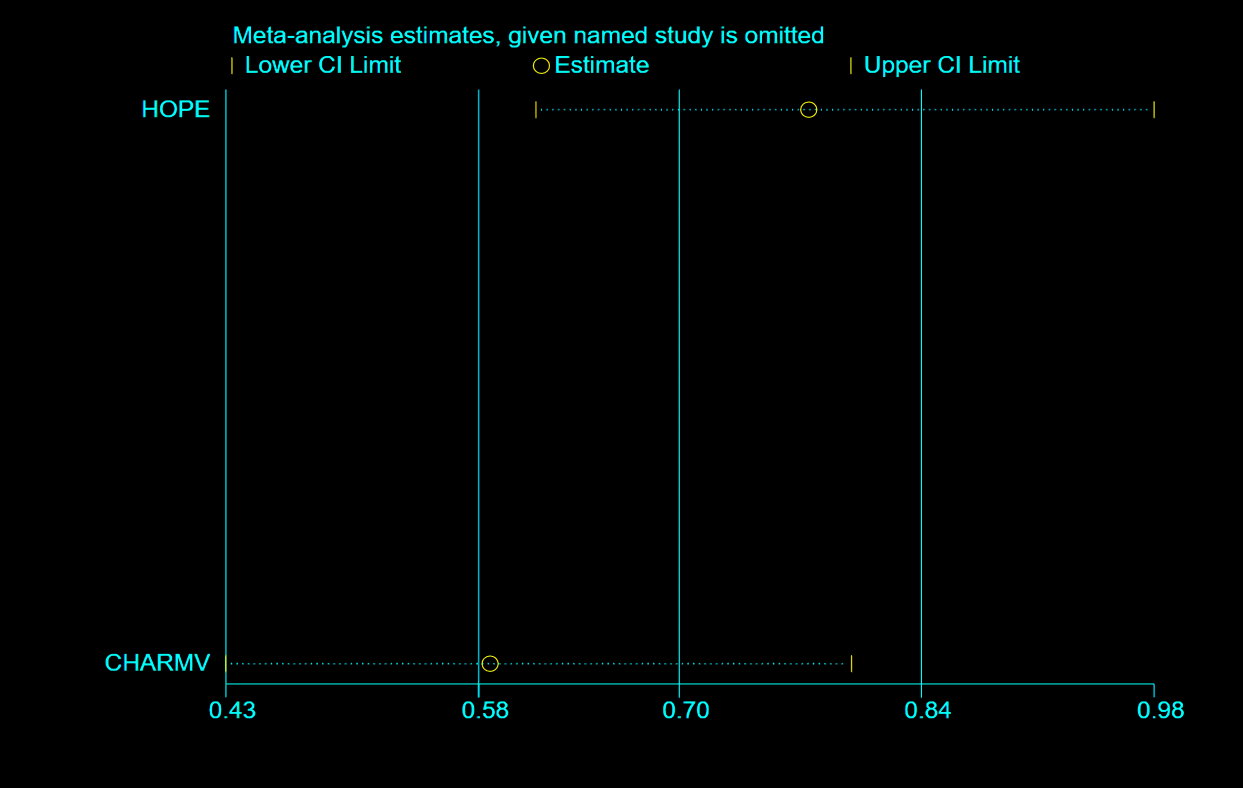
Figure S20.** Sensitivity analysis for the effect of ACEI/ARB compared with placebo on diabetes treatment.


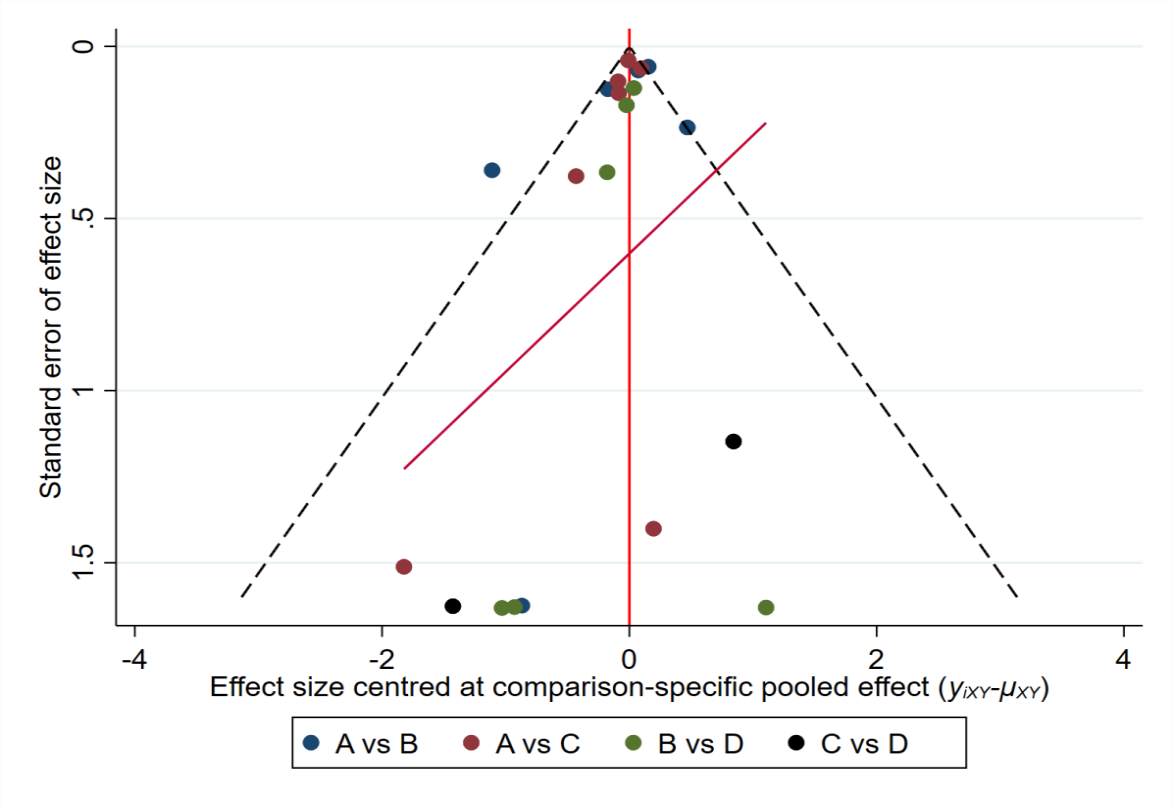
**Figure S21.** Funnel plot of the effect of sacubitril/valsartan compared with placebo on new-onset DM among all patients (non-DM).


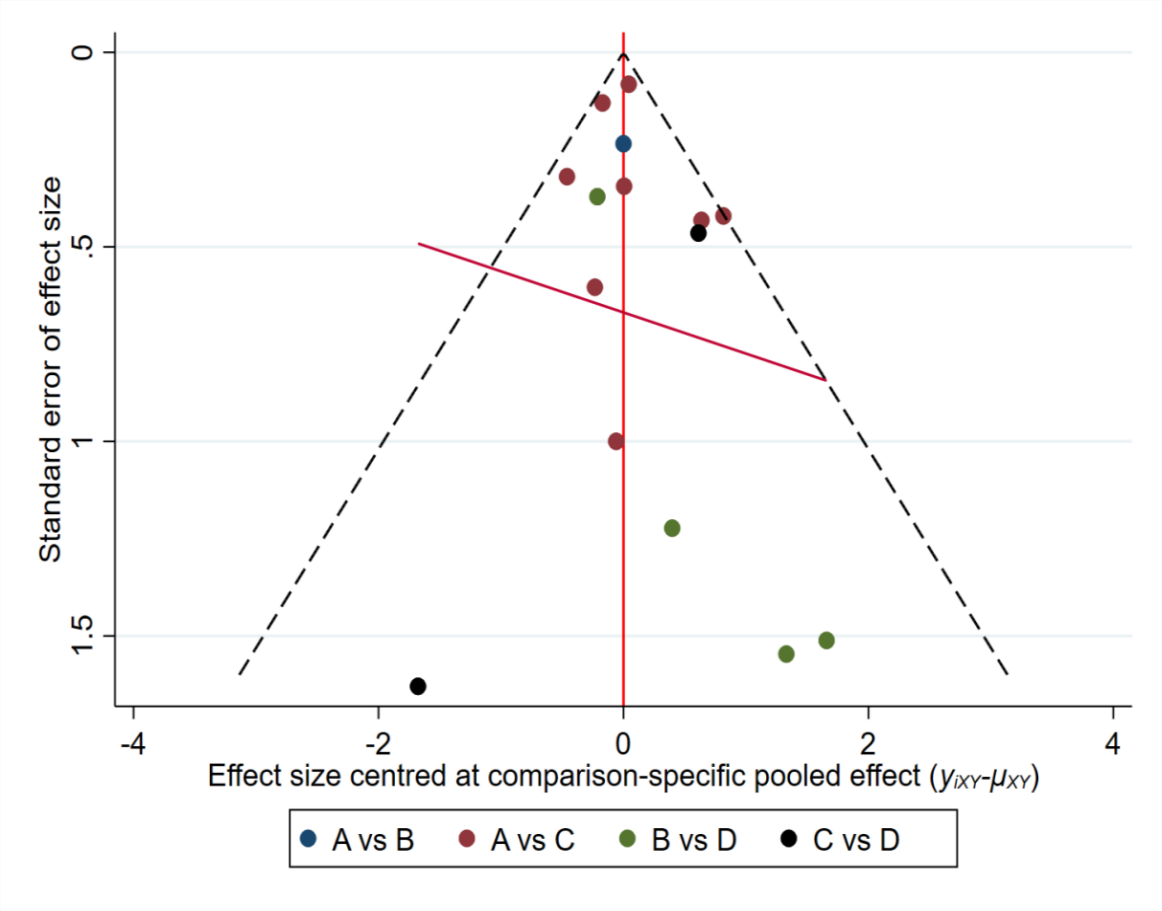


**Figure S22.** Funnel plot of the effect of sacubitril/valsartan compared with placebo on hypoglycaemia among all patients.


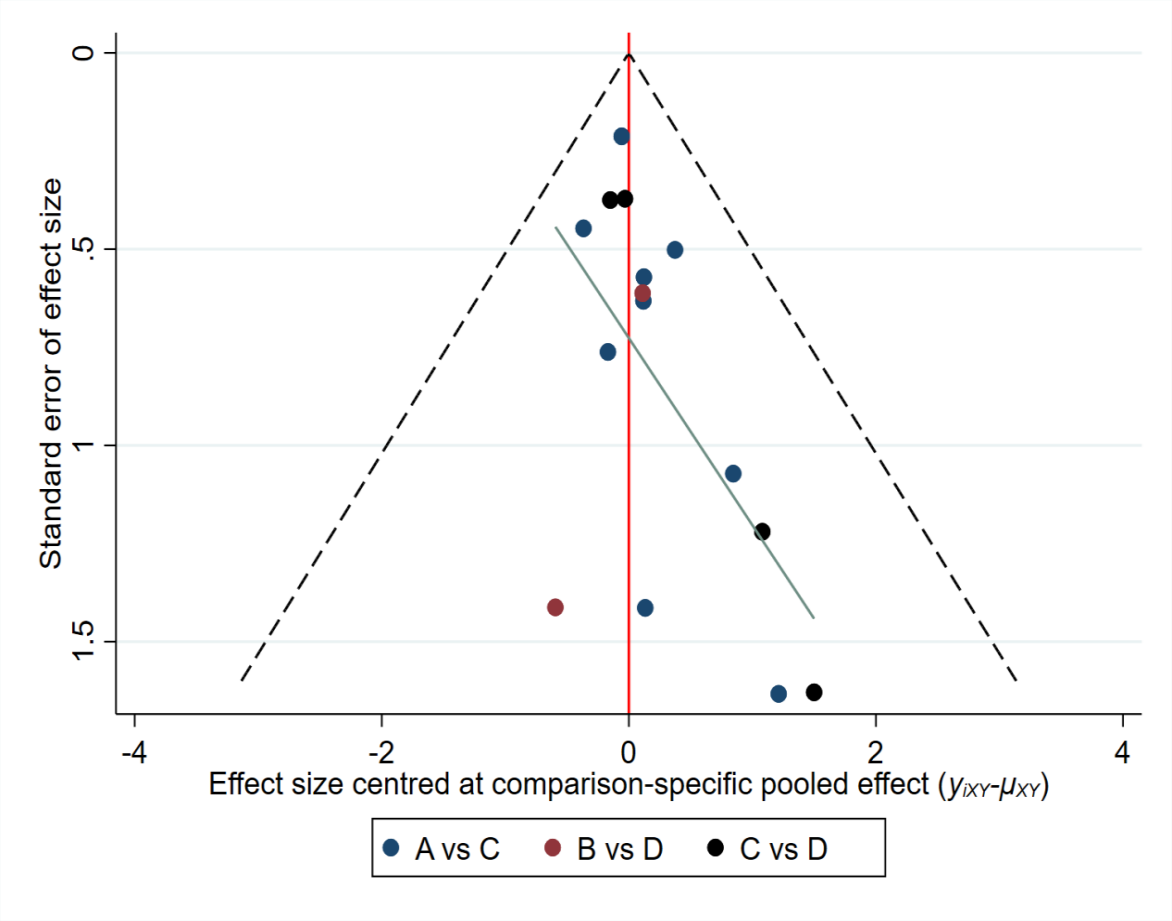
**Figure S23.** Funnel plot of the effect of sacubitril/valsartan compared with placebo on elevated glycaemia among all patients.


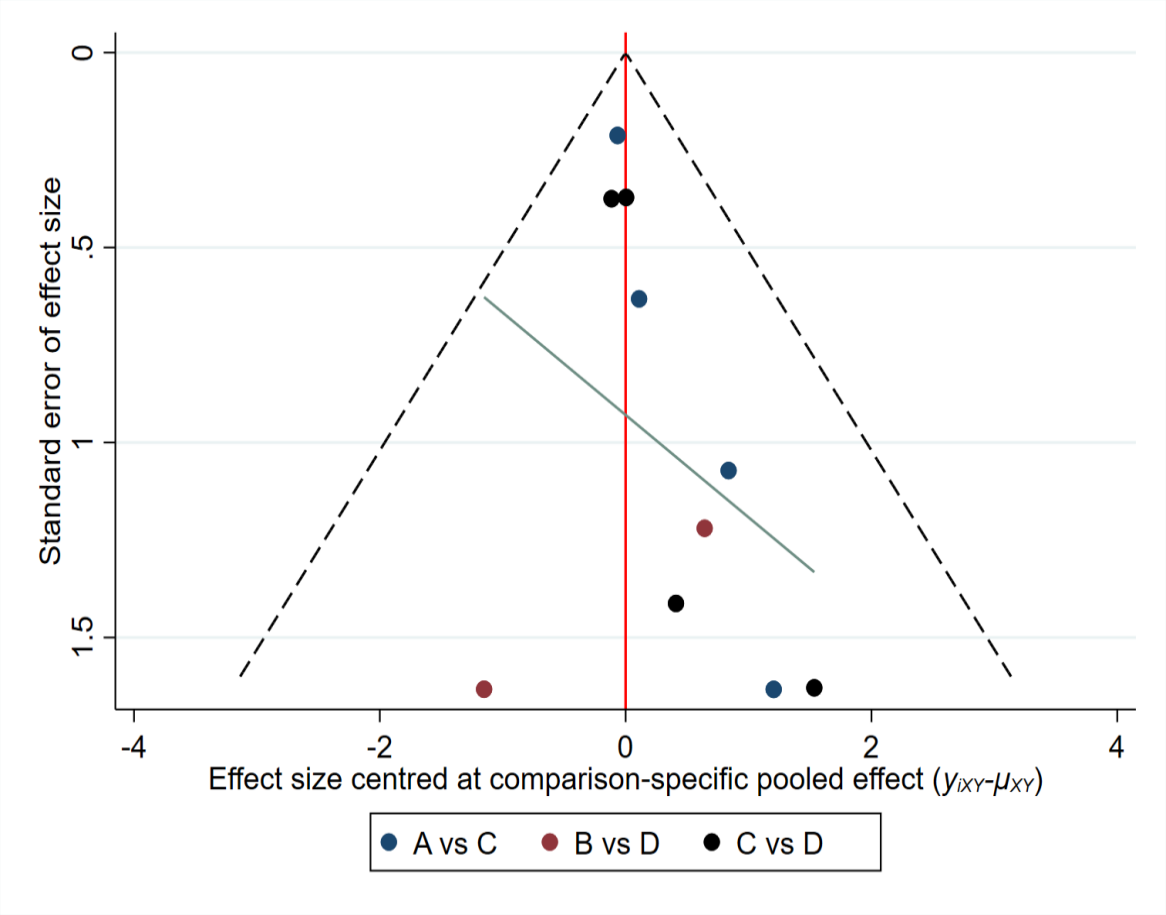


**Figure S24.** Funnel plot of the effect of sacubitril/valsartan compared with placebo on elevated glycaemia among patients with not all-DM.


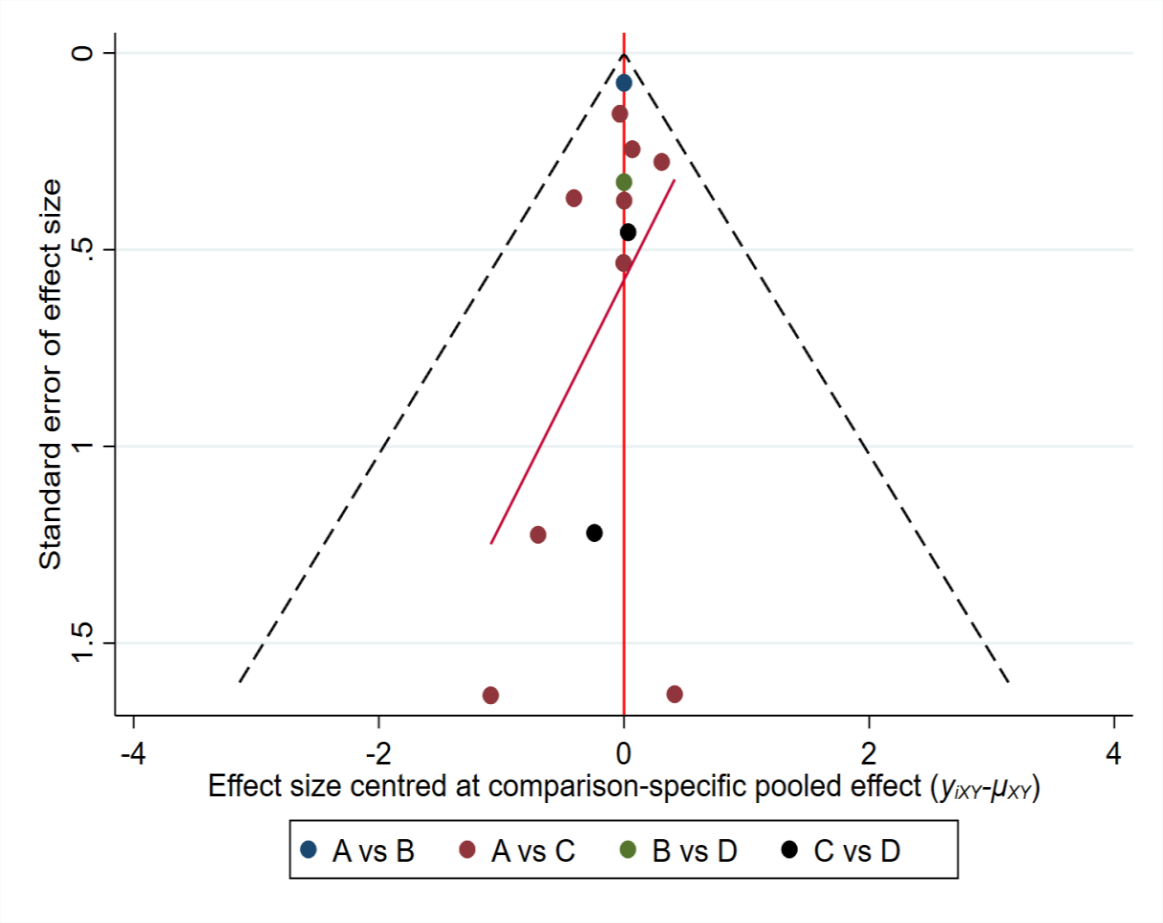
**Figure S25.** Funnel plot of the effect of sacubitril/valsartan compared with placebo on diabetes complication among all patients.
