# Supplementary material for: Effect of sacubitril/valsartan and ACEI/ARB on glycaemia and the development of diabetes: a systematic review and meta-analysis of randomised controlled trials
Source: BMC Med. 2022 Dec 17;20:487. doi: 10.1186/s12916-022-02682-w (PMC9758945; doi:10.1186/s12916-022-02682-w)
Supplement: Supplementary file 4 — Additional file 4: Figure S1. Methodological quality graph. Figure S2. Methodological quality summary. Figure S3-S33. Forest maps for all meta-analyses. [file 12916_2022_2682_MOESM4_ESM.docx]

***Additional file 4***

# Supplemental Figures

**
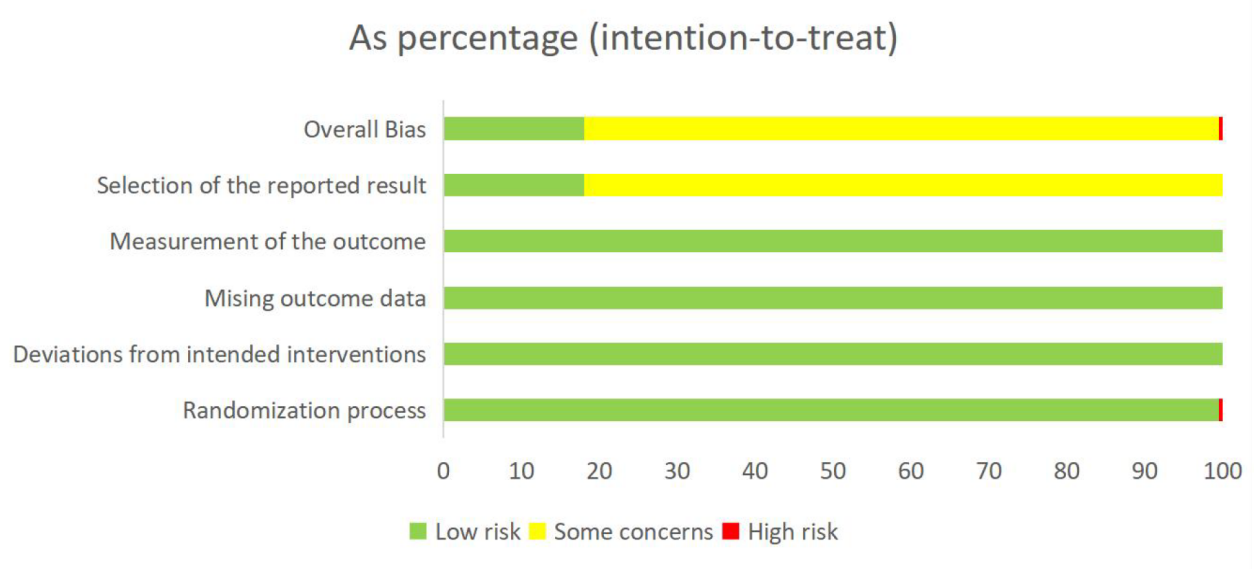
Figure S1.** Methodological quality graph: authors' judgements about each risk of bias item presented as percentages across all included studies.

**
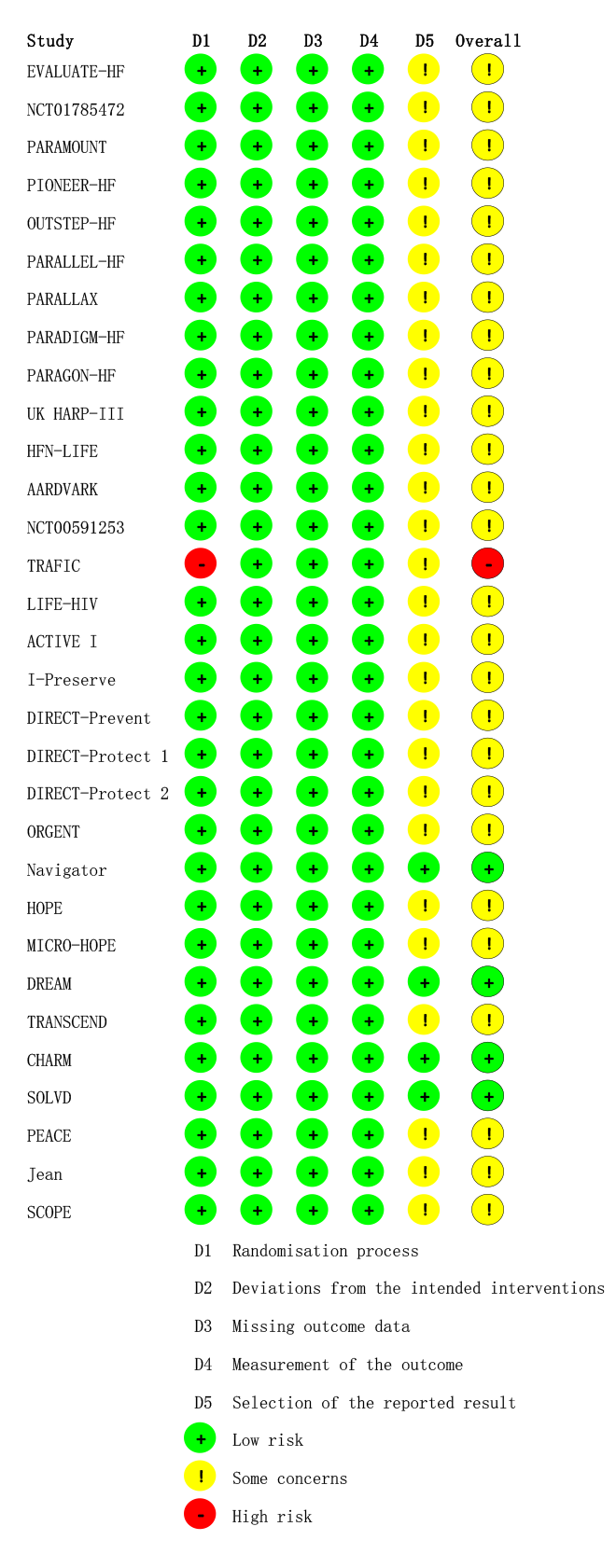
Figure S2.** Methodological quality summary: authors' judgements about each risk of bias item for each included study.

**
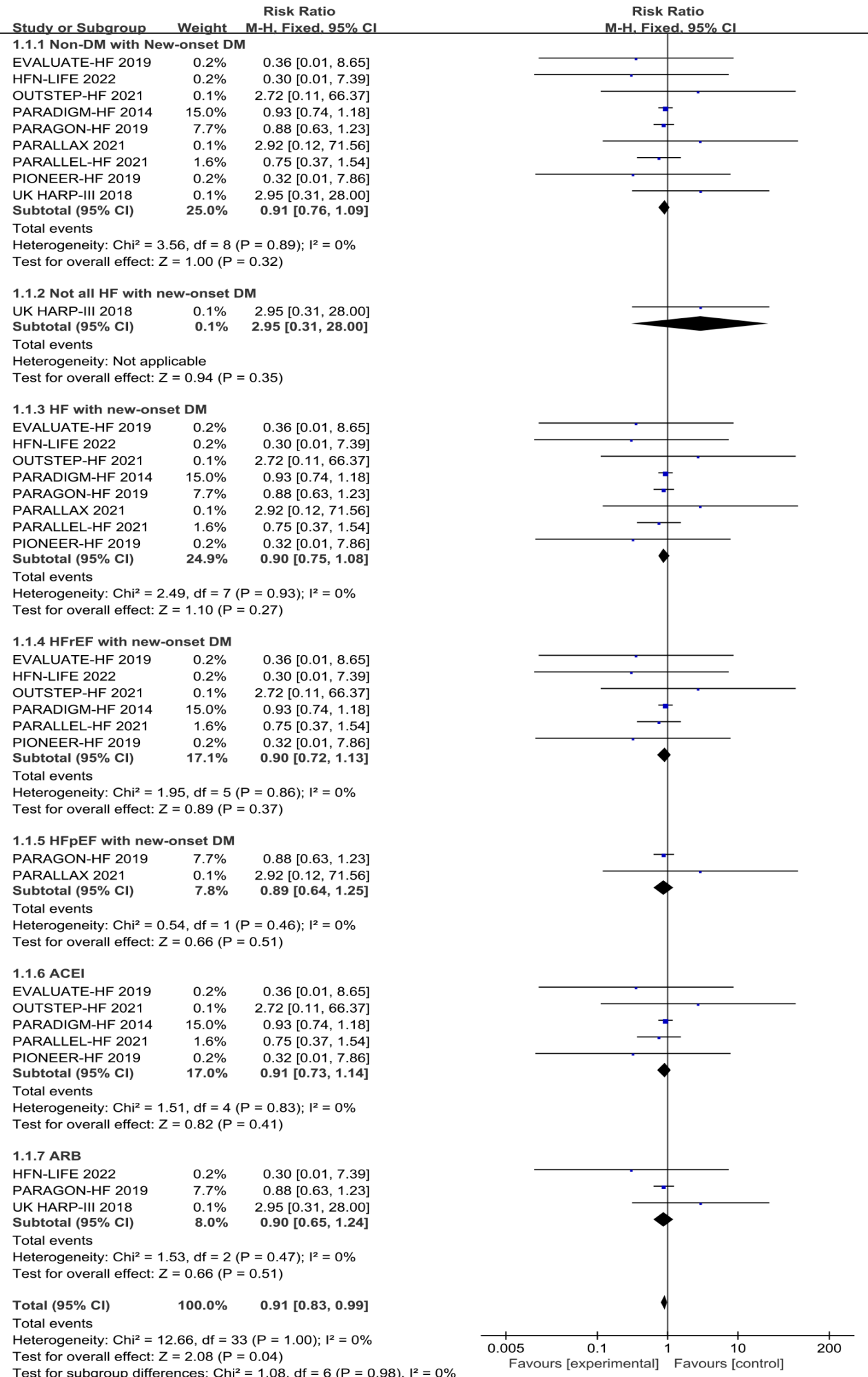
****Figure S3.** The effect of sacubitril/valsartan compared to ACEI/ARB on new-onset DM.

**
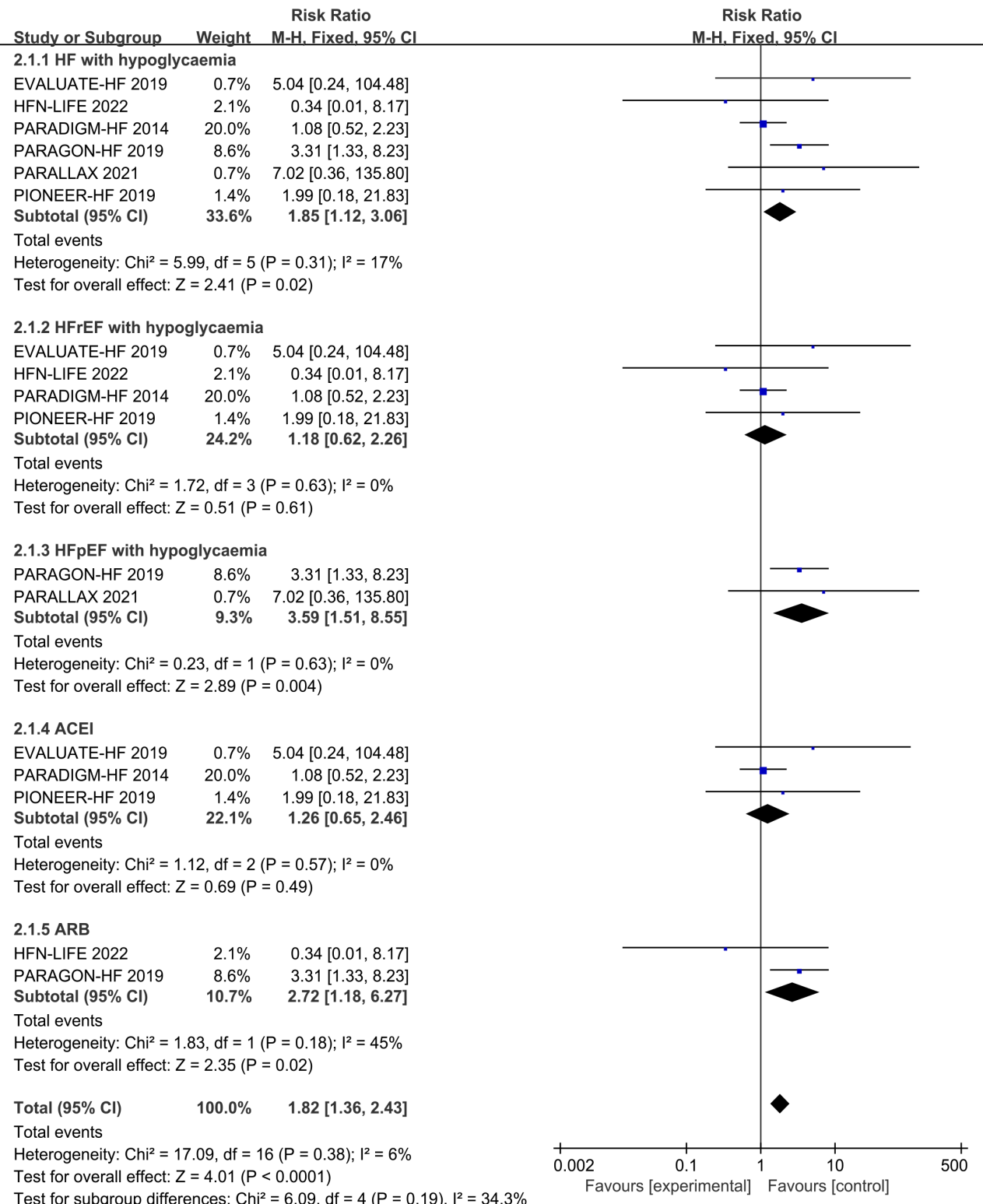
Figure S4.** The effect of sacubitril/valsartan compared to ACEI/ARB on hypoglycaemia.

**
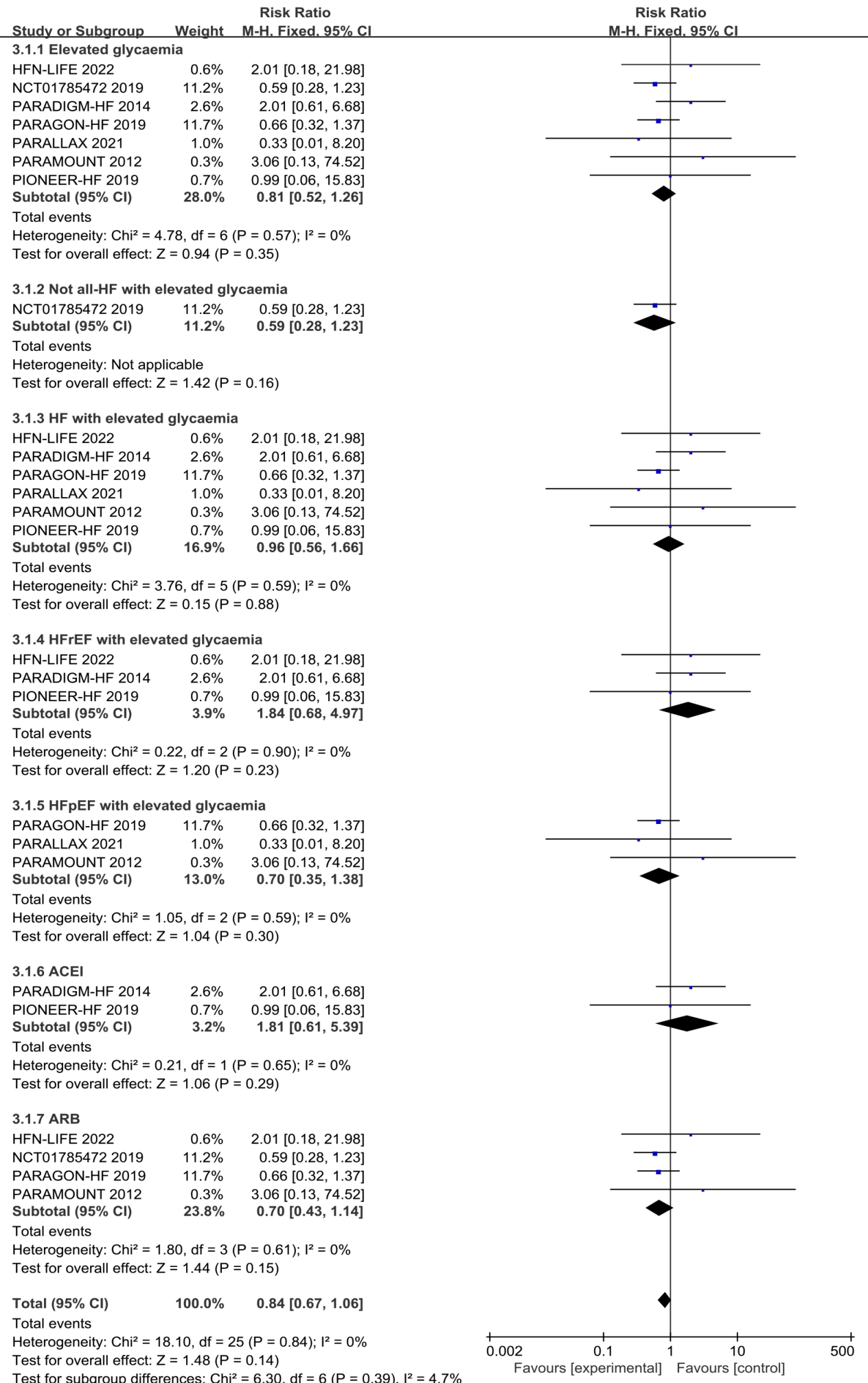
Figure S5.** The effect of sacubitril/valsartan compared to ACEI/ARB on elevated glycaemia.

**
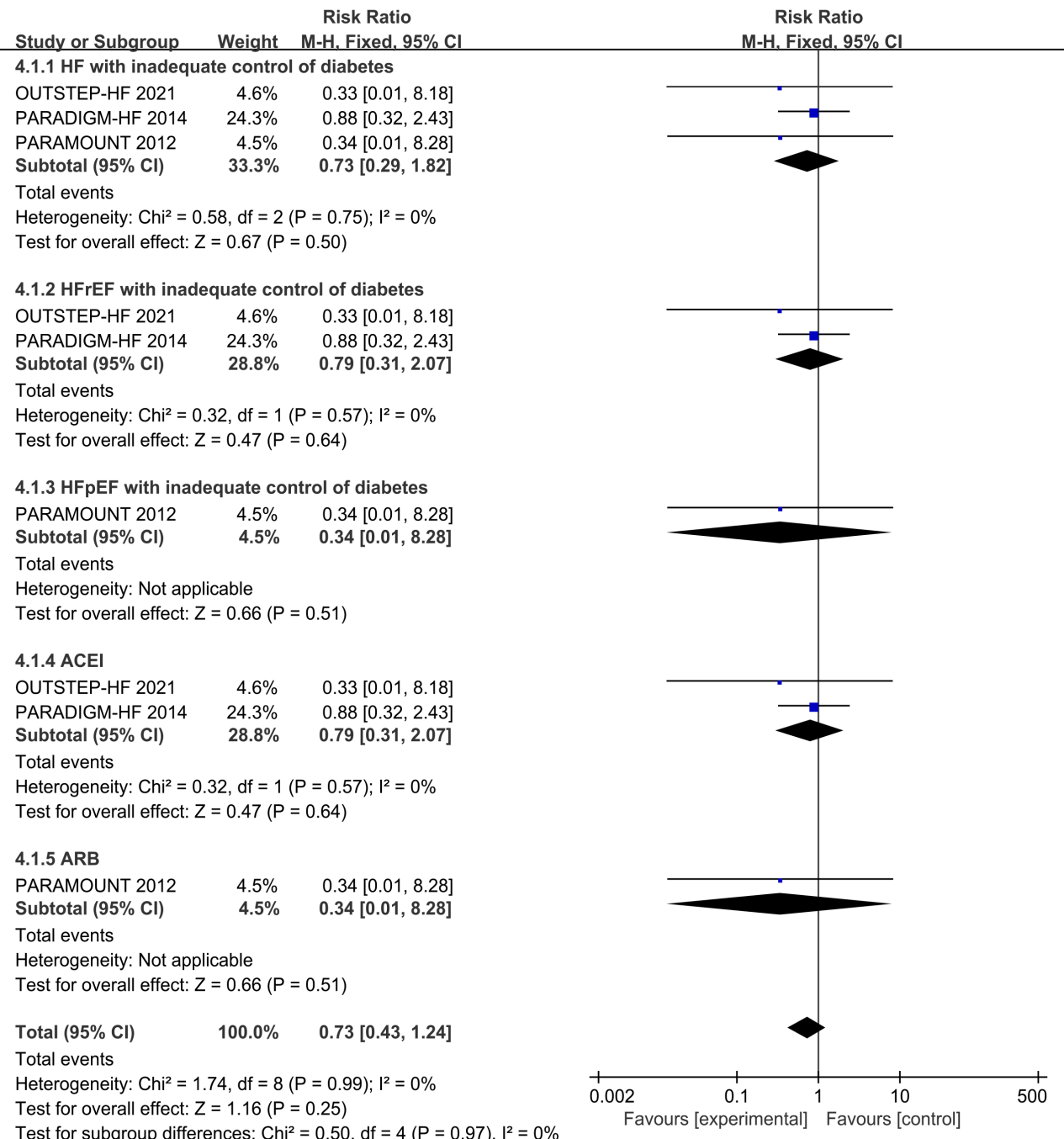
Figure S6.** The effect of sacubitril/valsartan compared to ACEI/ARB on DM inadequate control.

**
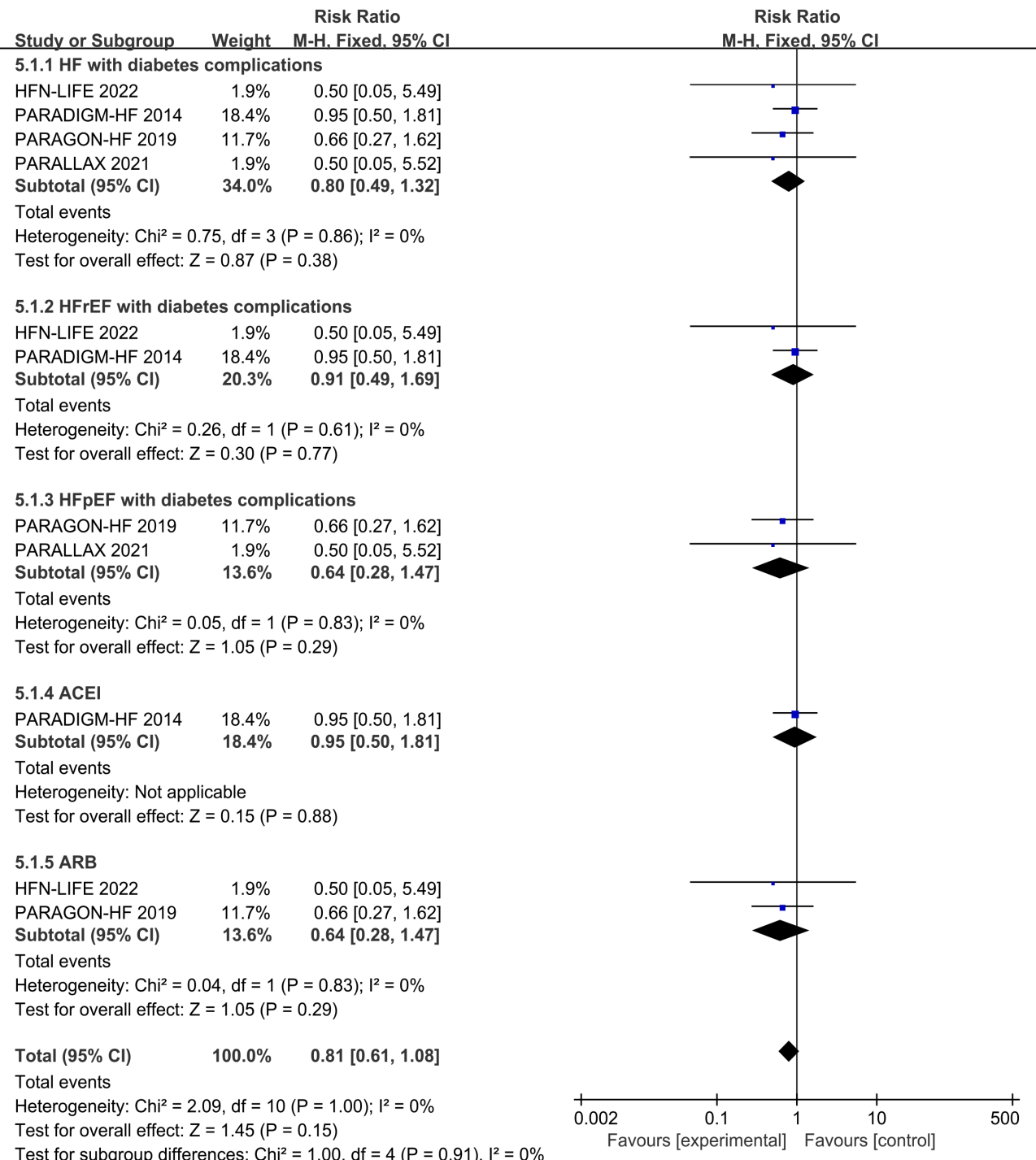
Figure S7.** The effect of sacubitril/valsartan compared to ACEI/ARB on diabetes complications.

**
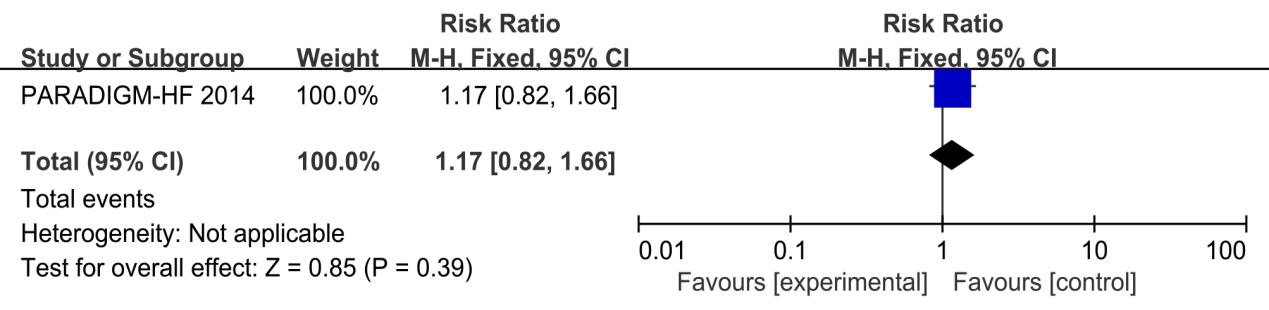
Figure S8.** The effect of sacubitril/valsartan compared to ACEI/ARB on diabetes treatment.

**
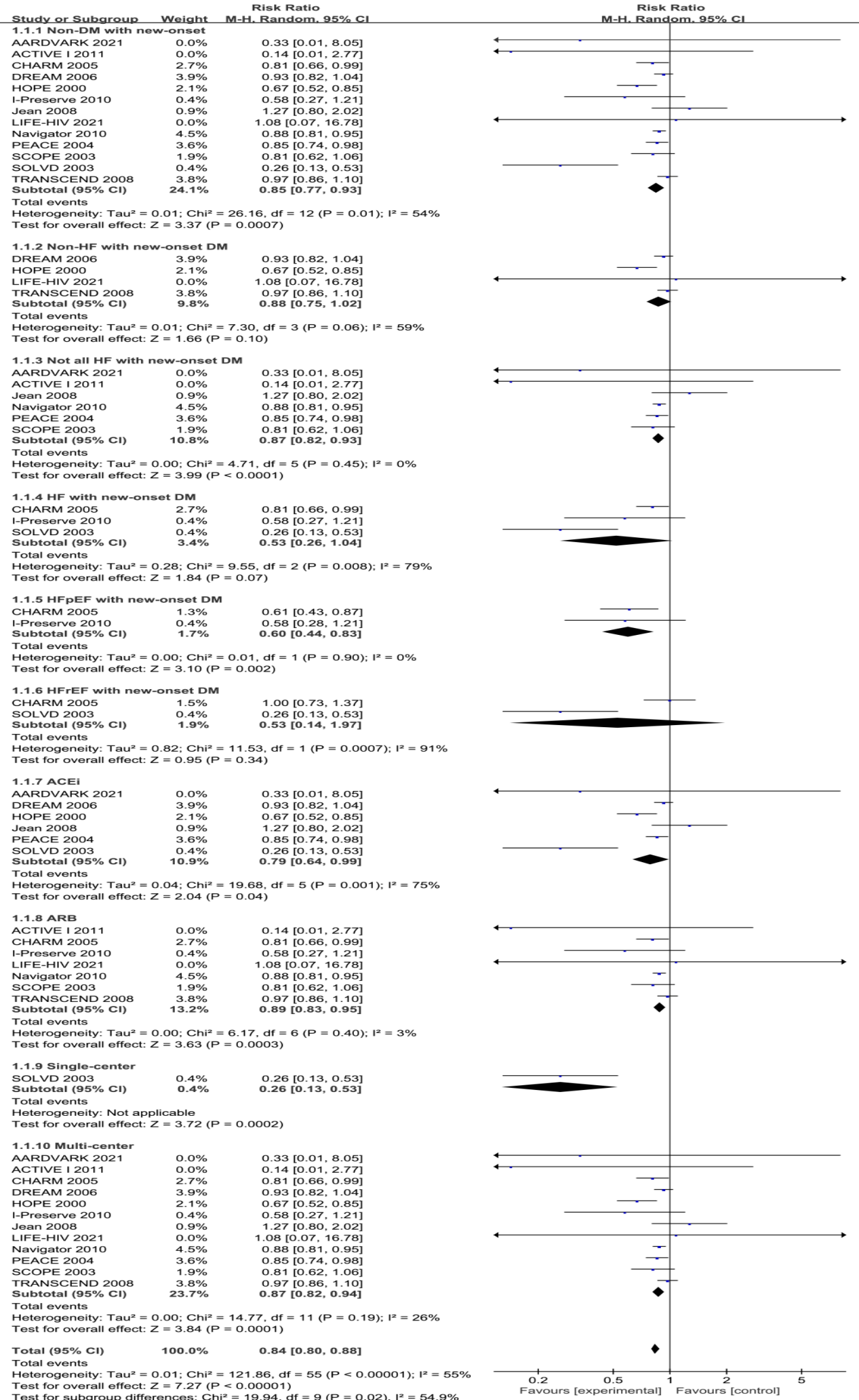
Figure S9.** The effect of ACEI/ARB compared to placebo on new-onset DM.

**
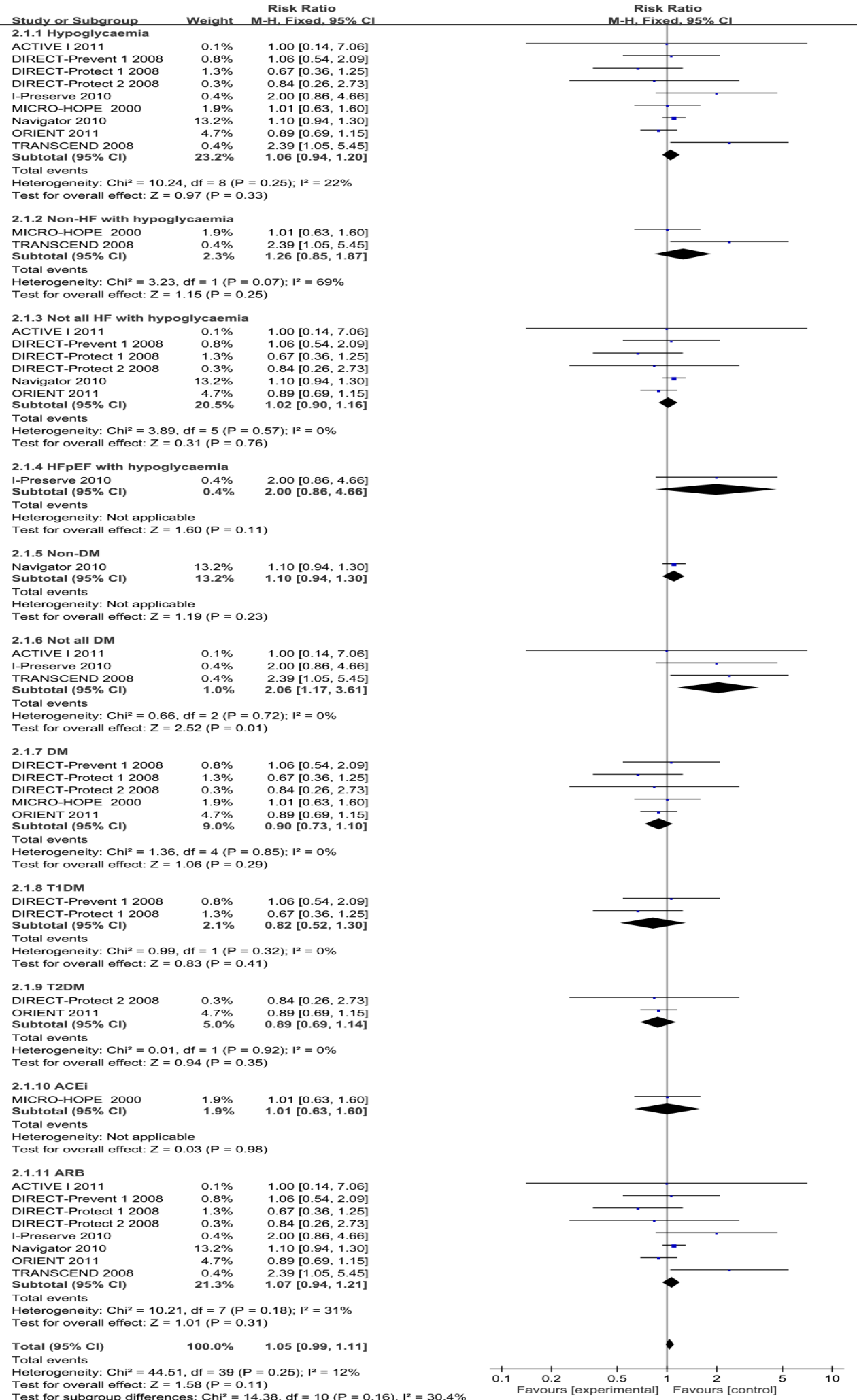
Figure S10.** The effect of ACEI/ARB compared to placebo on hypoglycaemia.


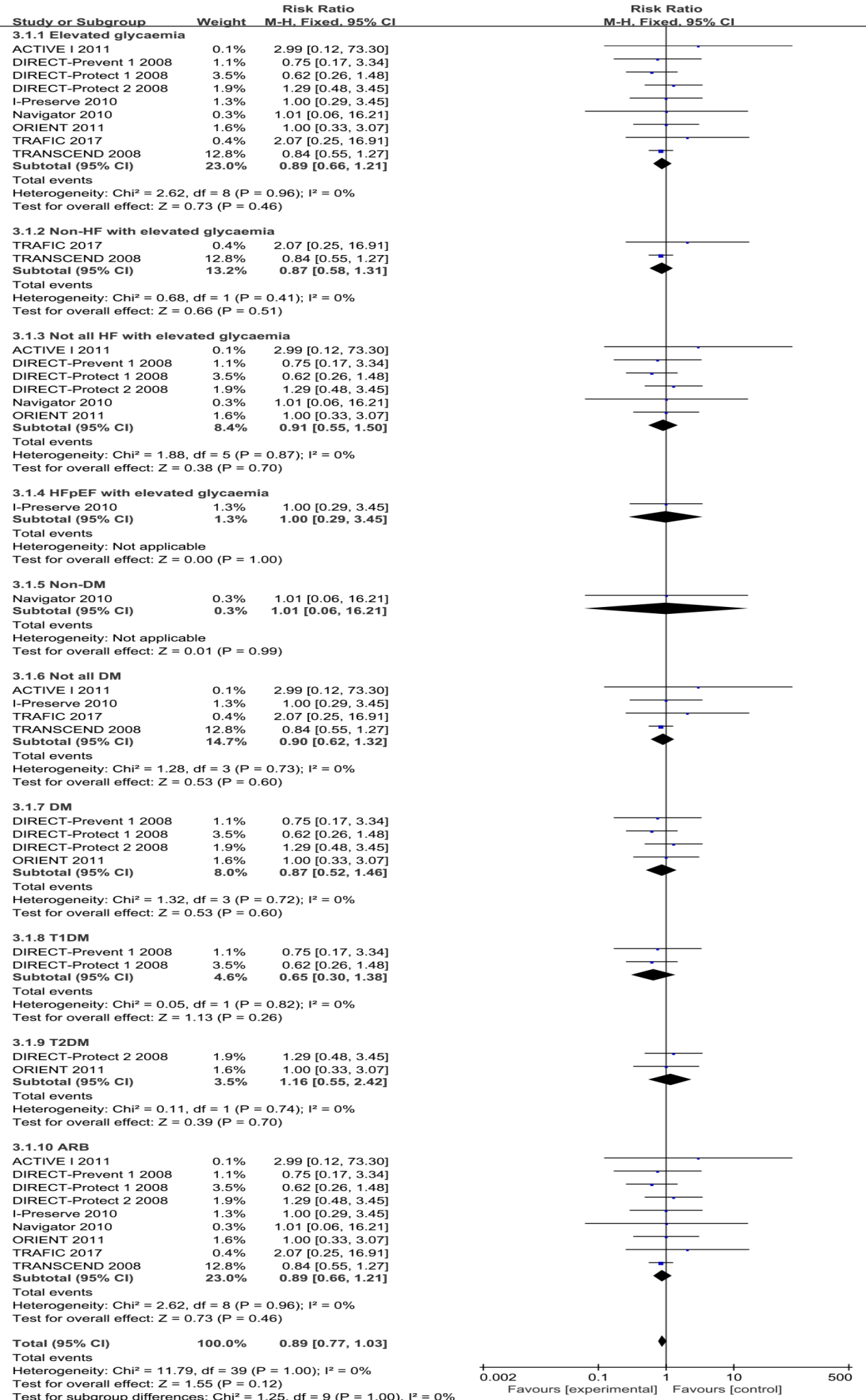
**Figure S11.** The effect of ACEI/ARB compared to placebo on elevated glycaemia.

**
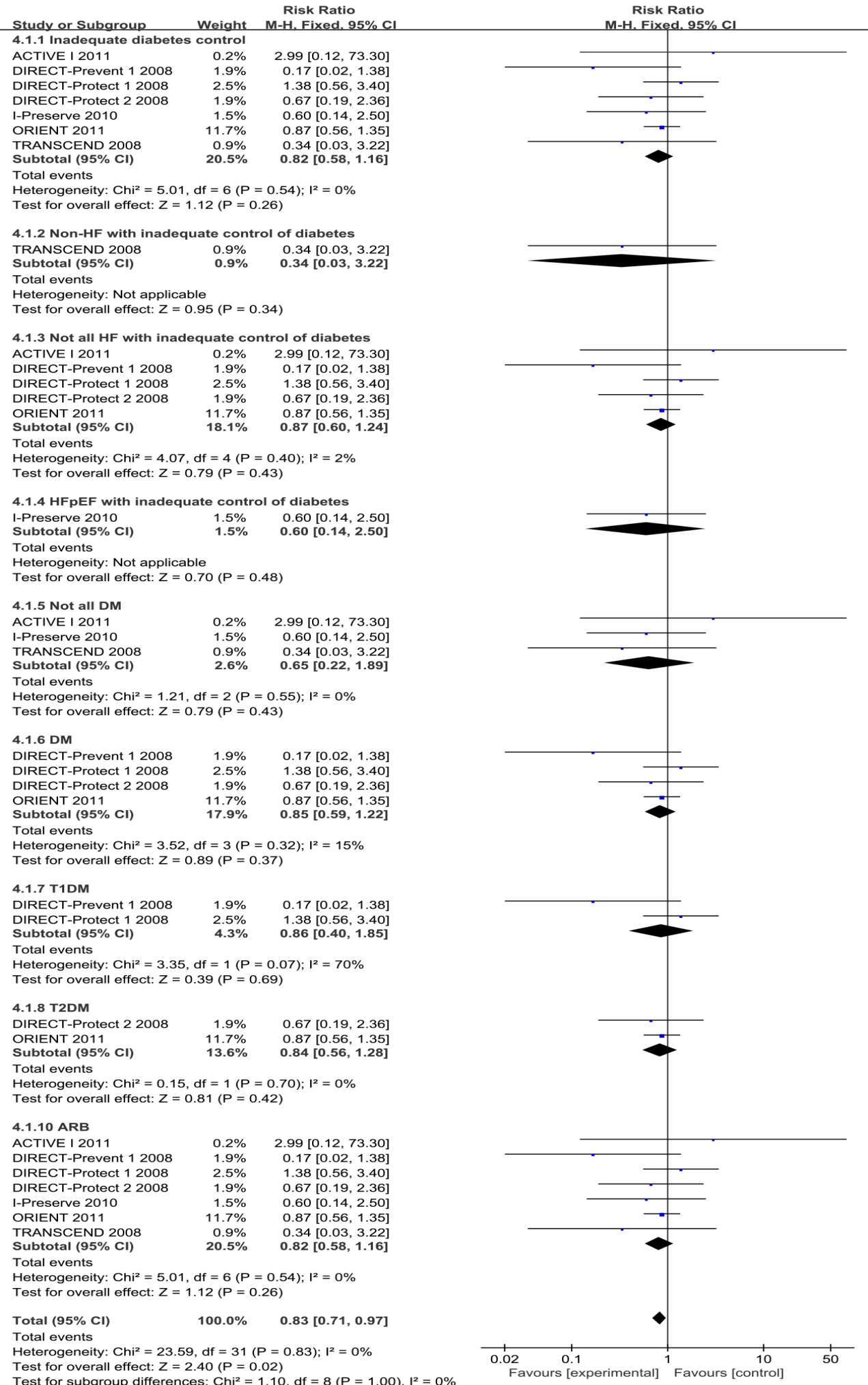
Figure S12.** The effect of ACEI/ARB compared to placebo on DM inadequate control.

**
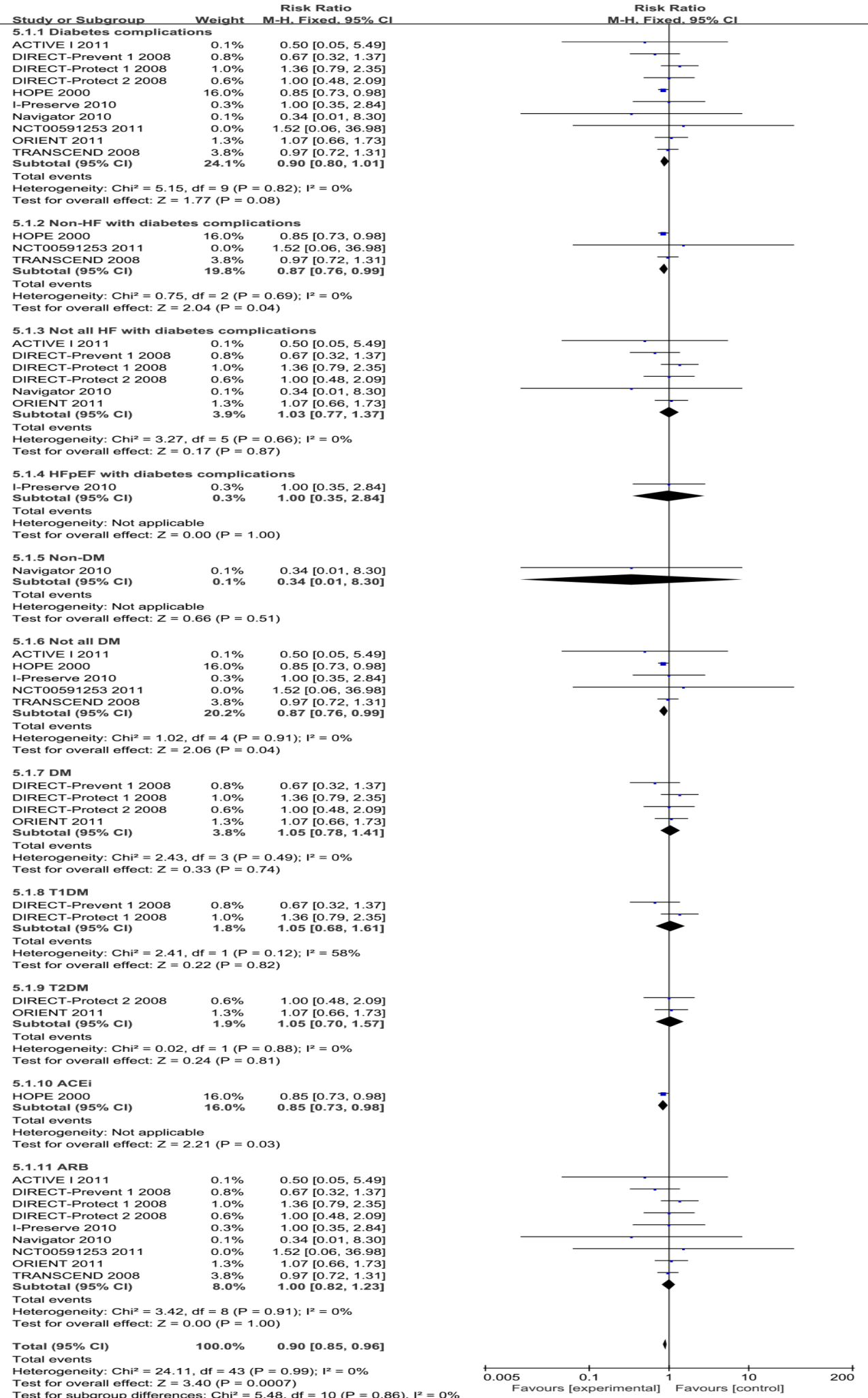
Figure S13.** The effect of ACEI/ARB compared to placebo on diabetes complications.

**
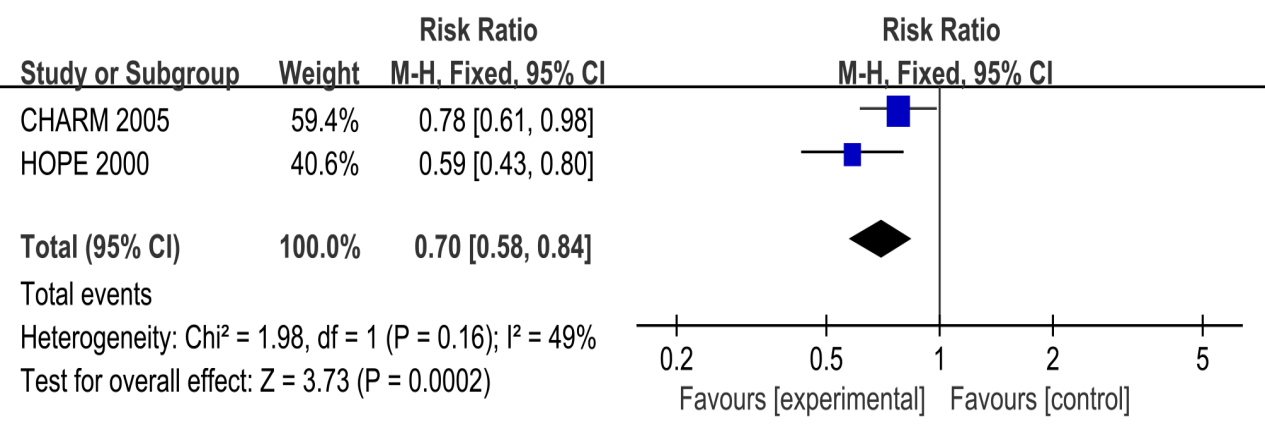
Figure S14.** The effect of ACEI/ARB compared to placebo on diabetes treatment.

**
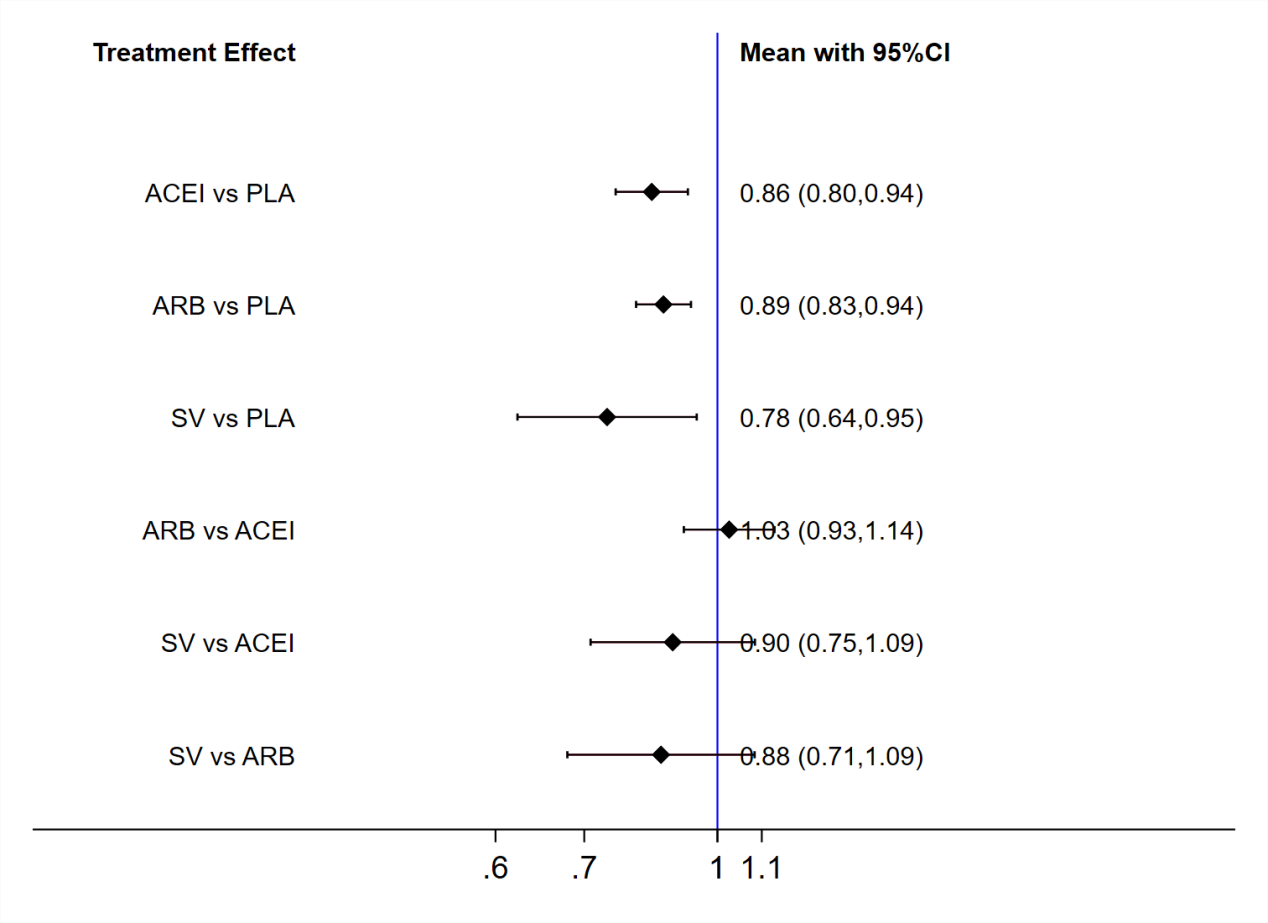
****Figure S15.** The effect of sacubitril/valsartan (SV) compared to placebo (PLA) on new-onset DM among all patients (/non-DM).

**
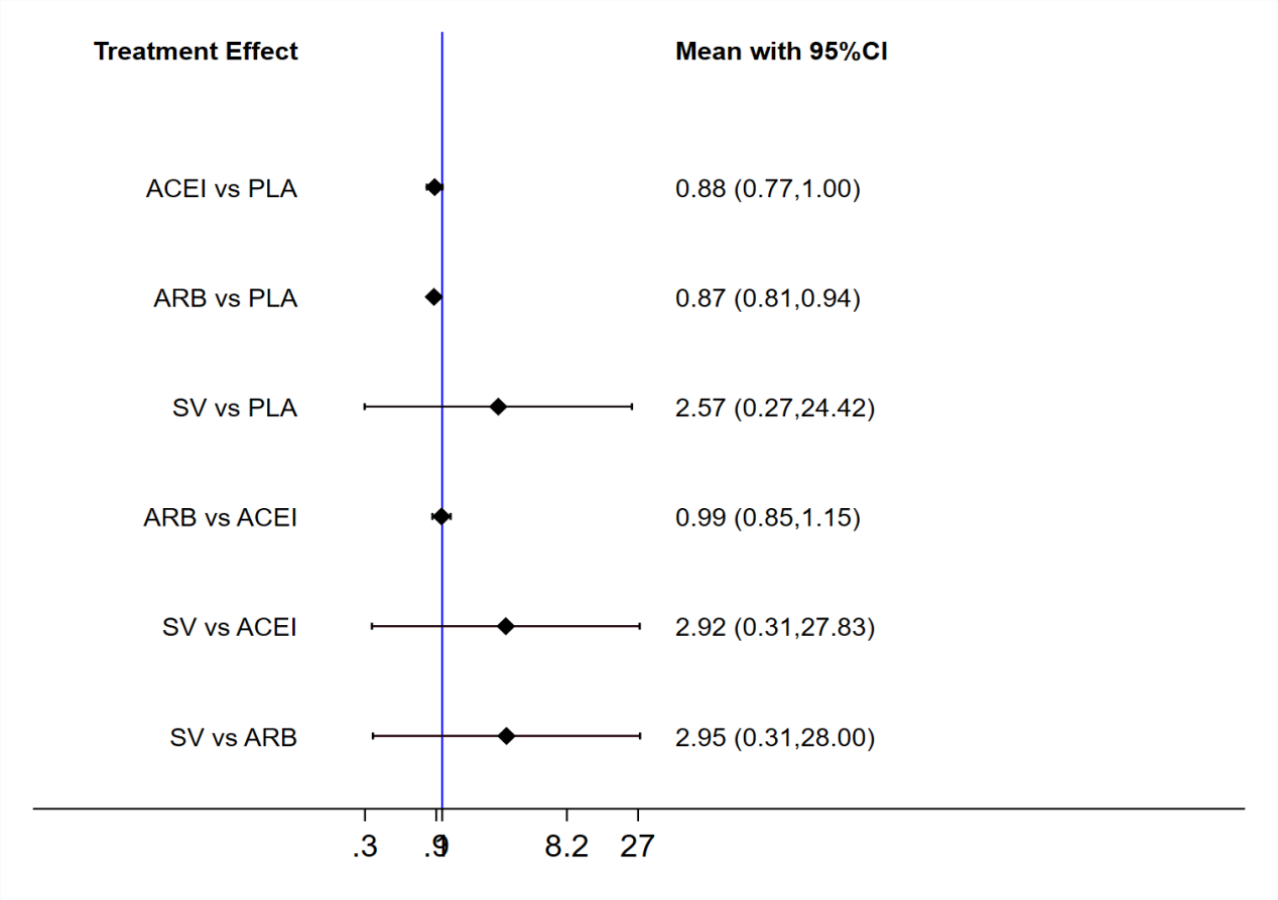
Figure S16.** The effect of sacubitril/valsartan (SV) compared to placebo (PLA) on new-onset DM among patients with not all-HF.

**
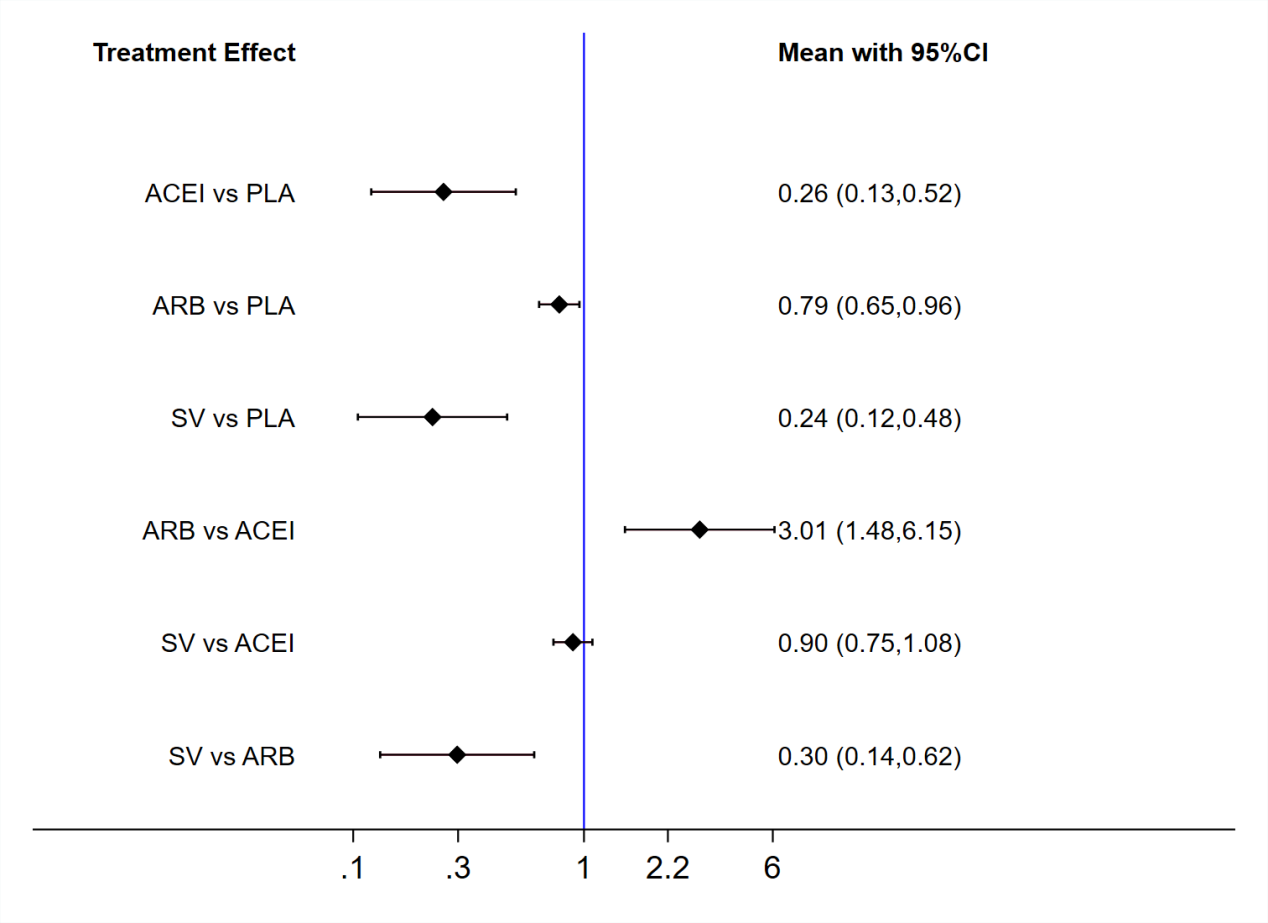
Figure S17.** The effect of sacubitril/valsartan (SV) compared to placebo (PLA) on new-onset DM among patients with HF.

**
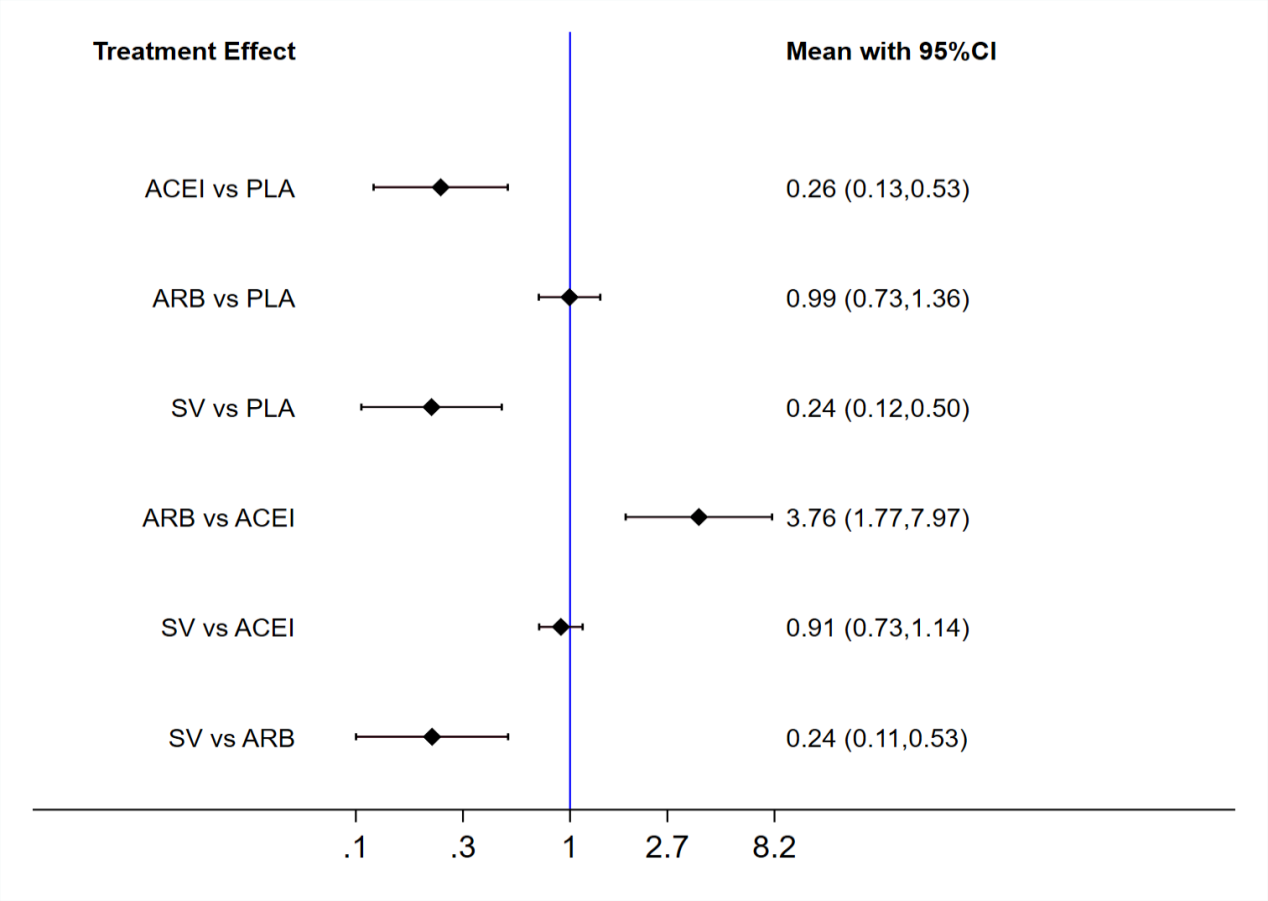
Figure S18.** The effect of sacubitril/valsartan (SV) compared to placebo (PLA) on new-onset DM among patients with HFrEF.
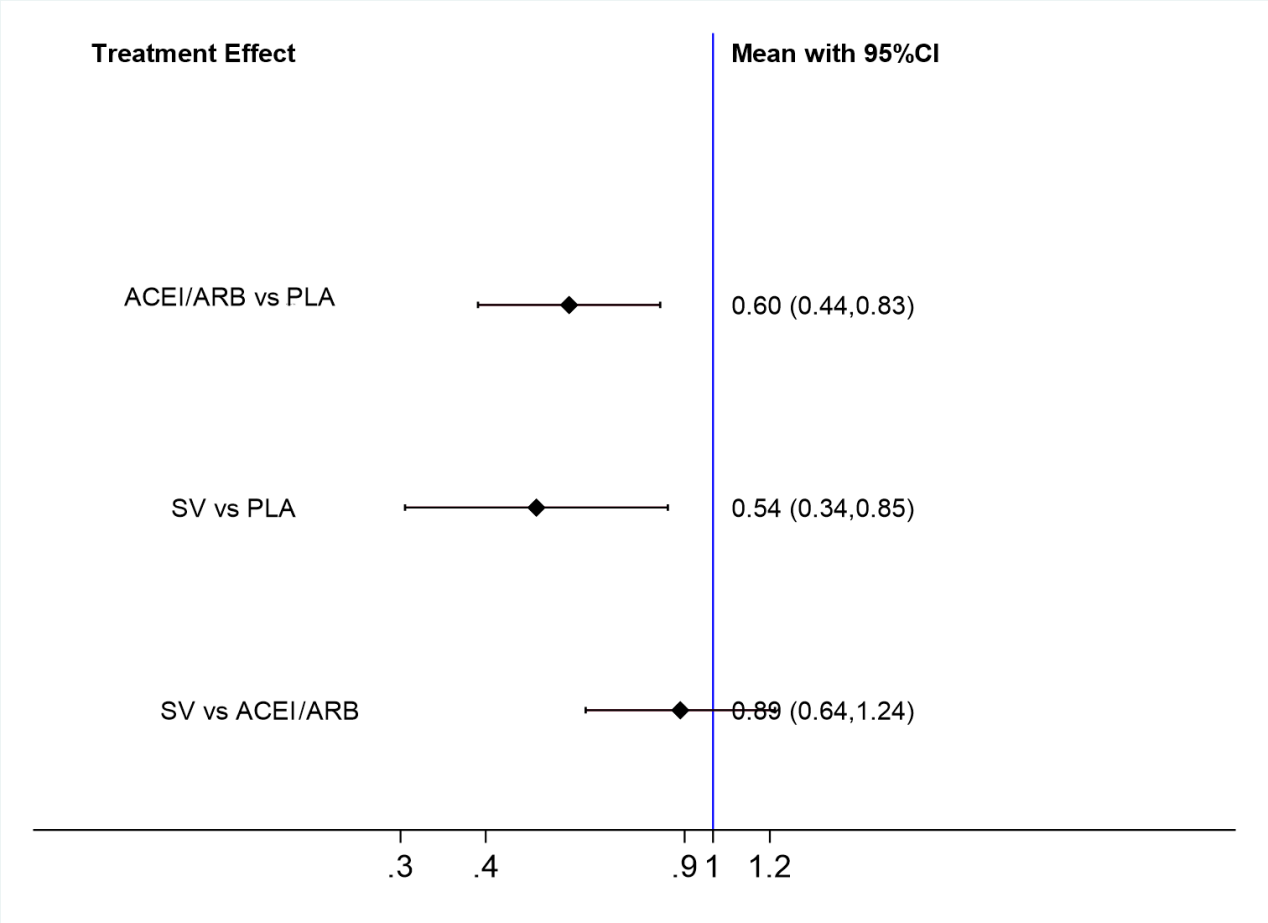


**Figure S19.** The effect of sacubitril/valsartan (SV) compared to placebo (PLA) on new-onset DM among patients with HFpEF.


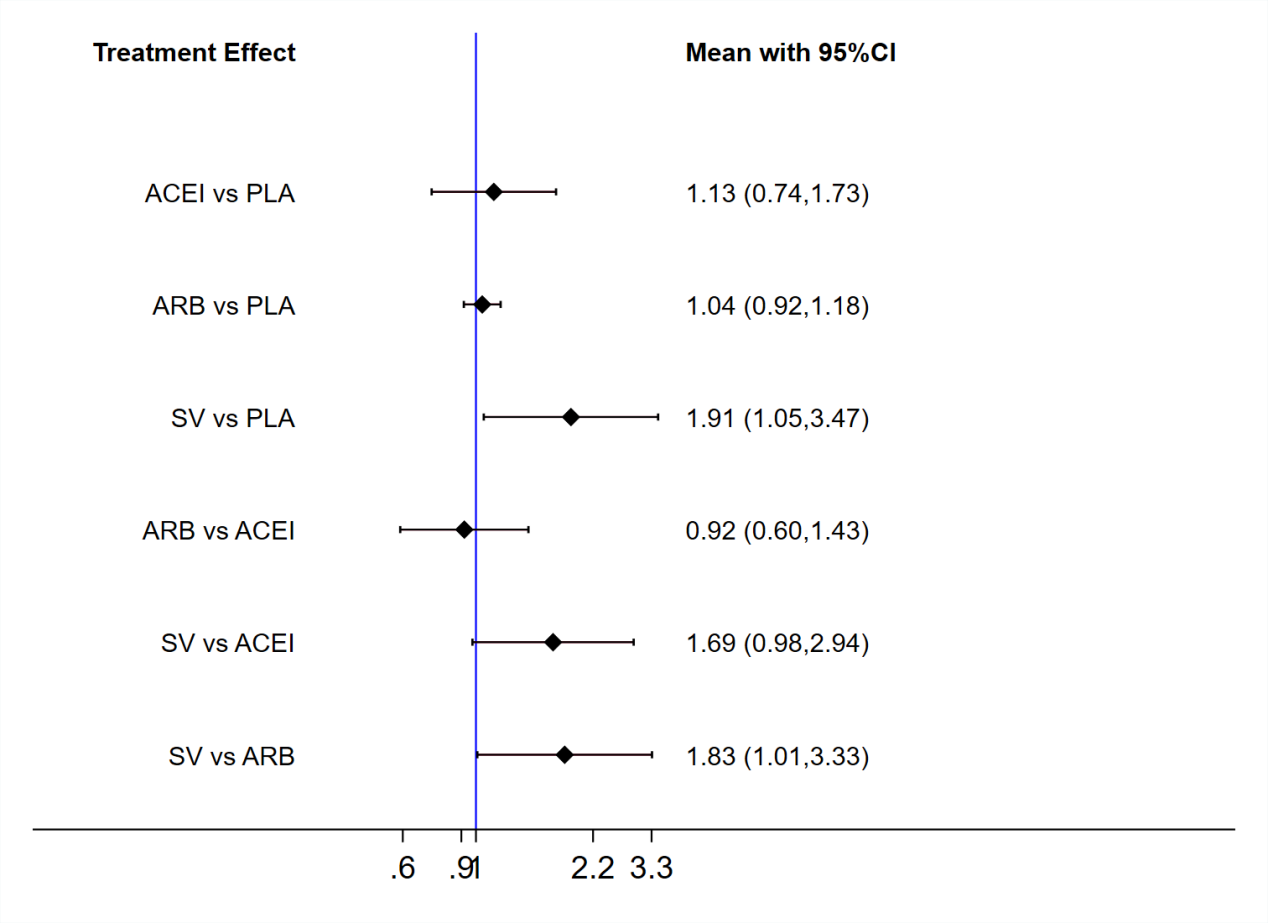


**Figure S20.** The effect of sacubitril/valsartan (SV) compared to placebo (PLA) on hypoglycaemia among all patients.

**
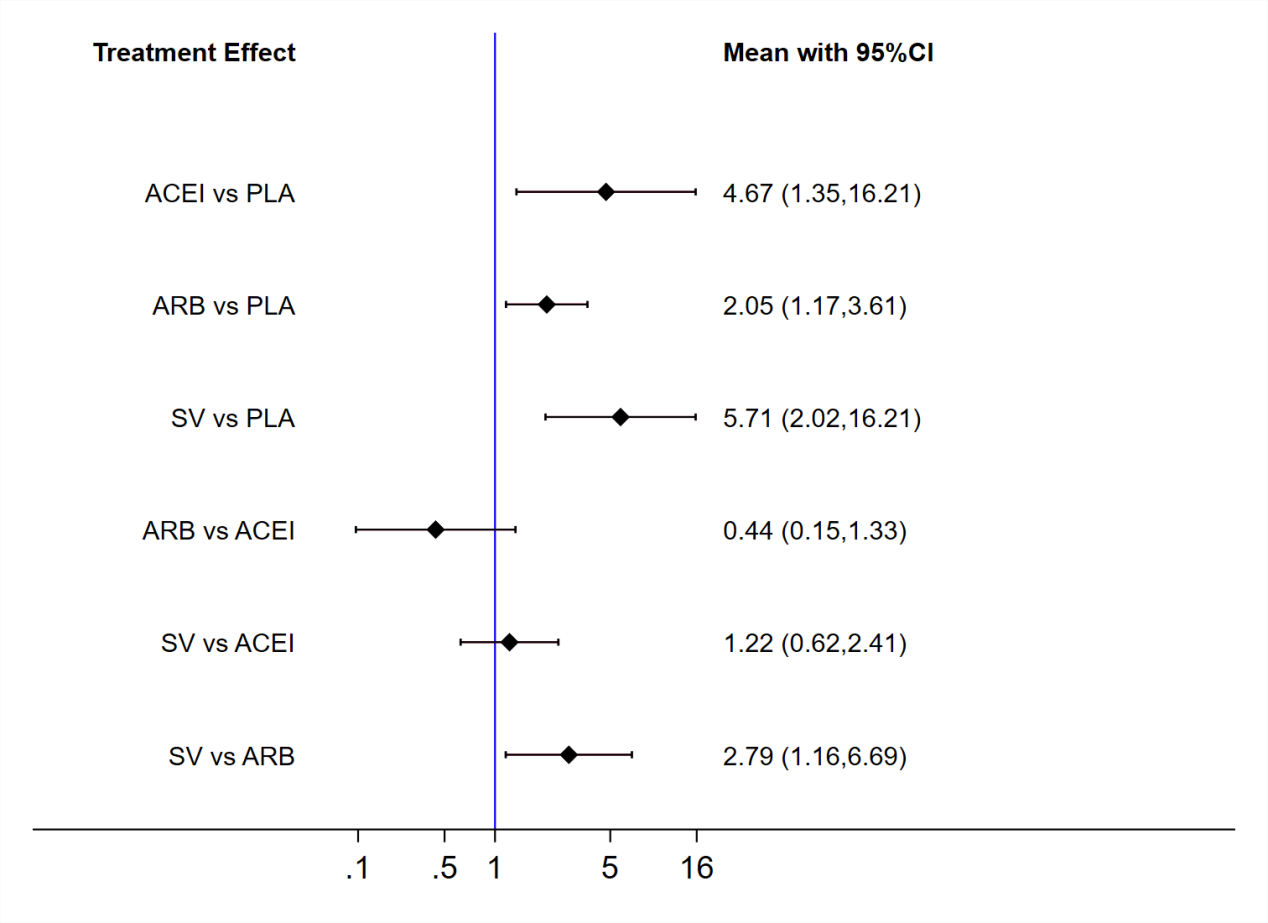
Figure S21.** The effect of sacubitril/valsartan (SV) compared to placebo (PLA) on hypoglycaemia among patients with not all-DM.
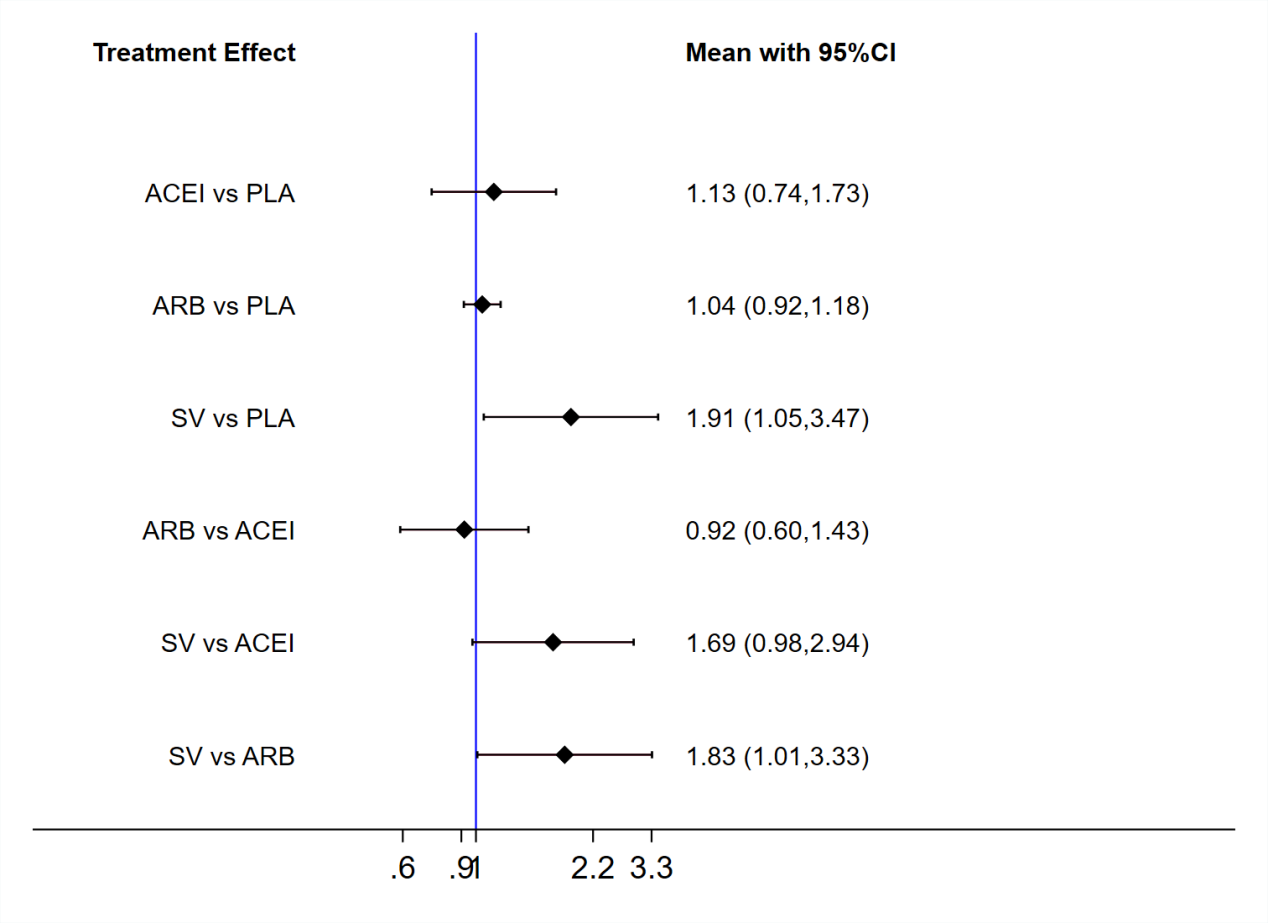


**
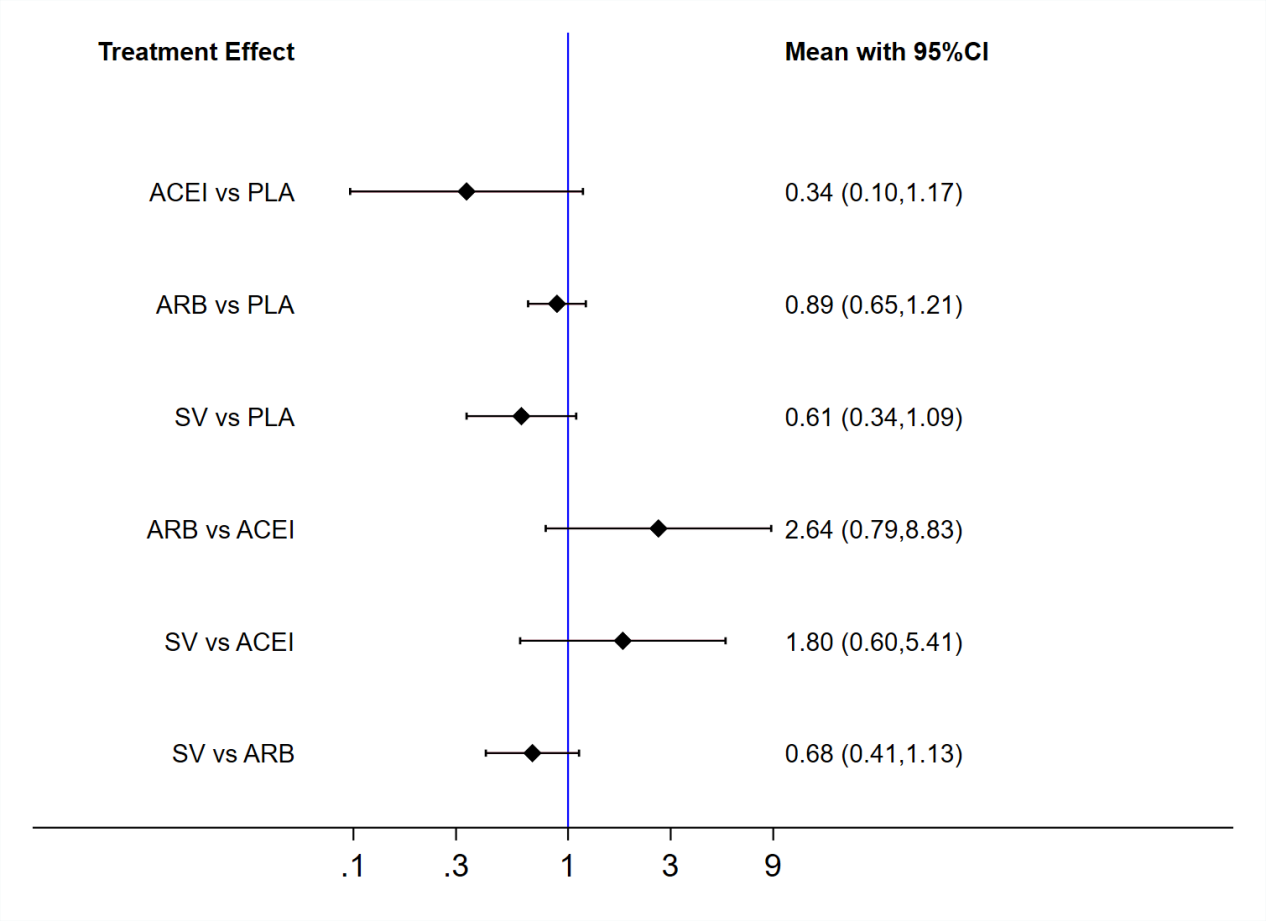

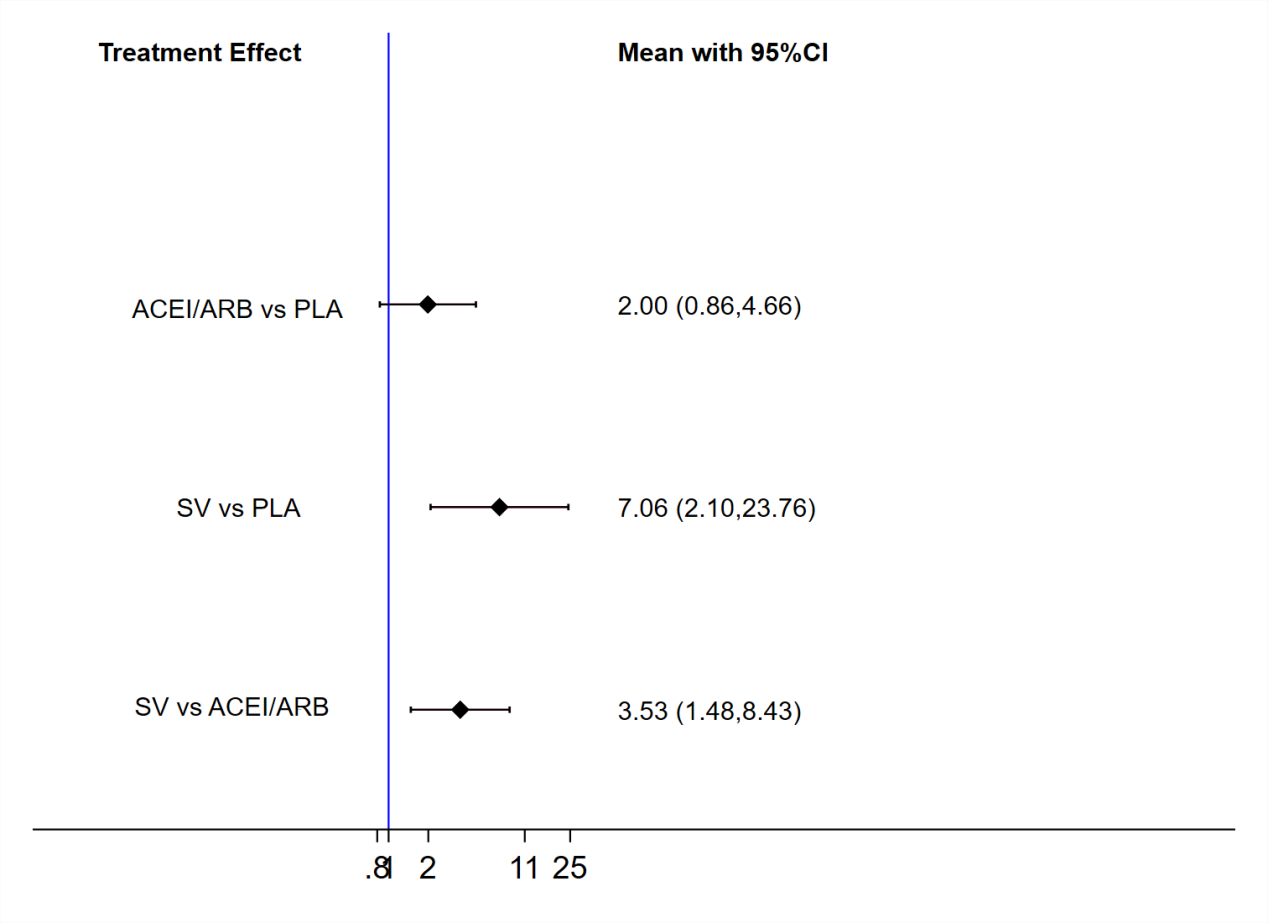
Figure S22.** The effect of sacubitril/valsartan (SV) compared to placebo (PLA) on hypoglycaemia among patients with HFpEF.

**Figure S23.** The effect of sacubitril/valsartan (SV) compared to placebo (PLA) on elevated glycaemia among all patients.

**
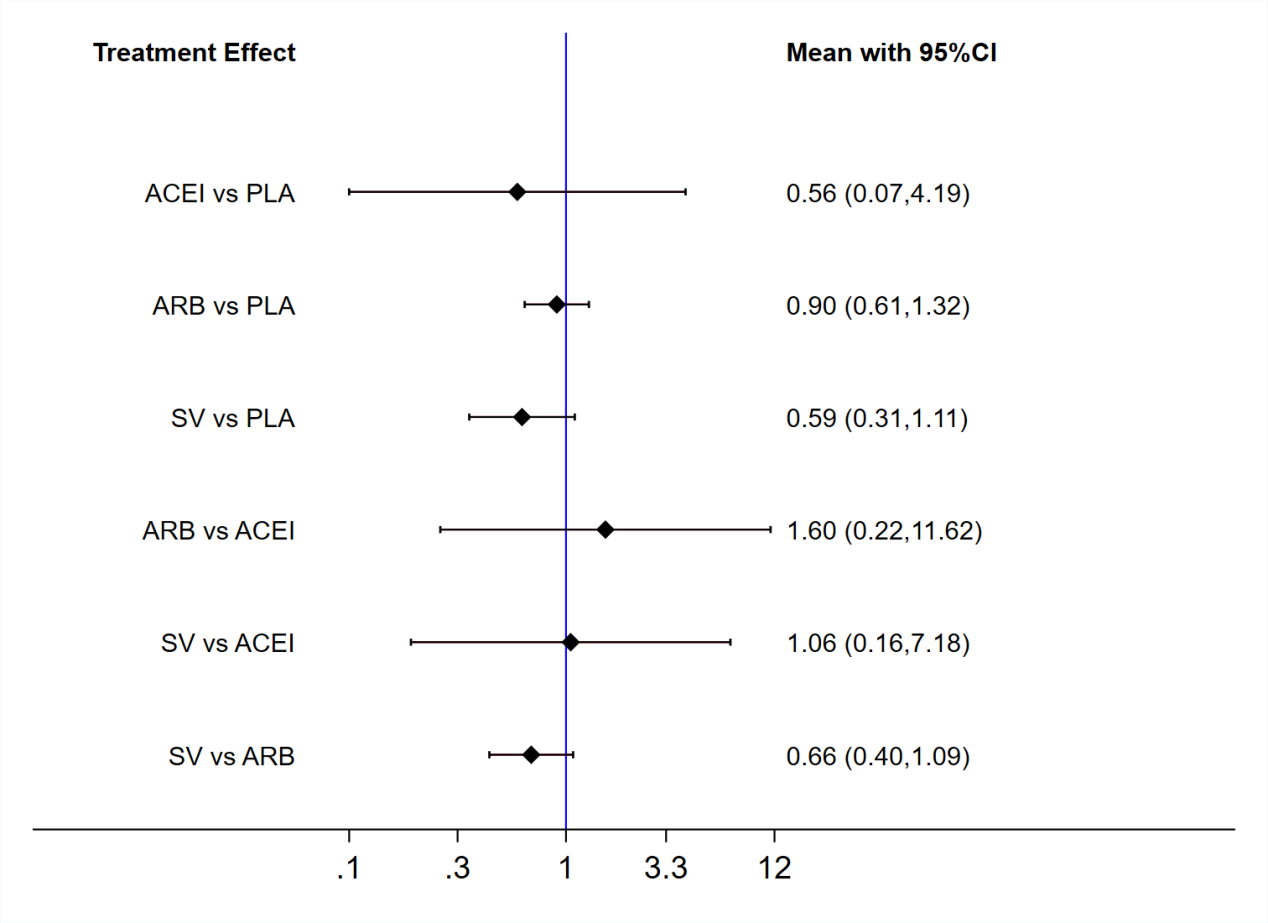
Figure S24.** The effect of sacubitril/valsartan (SV) compared to placebo (PLA) on elevated glycaemia among patients with not all-DM.

**
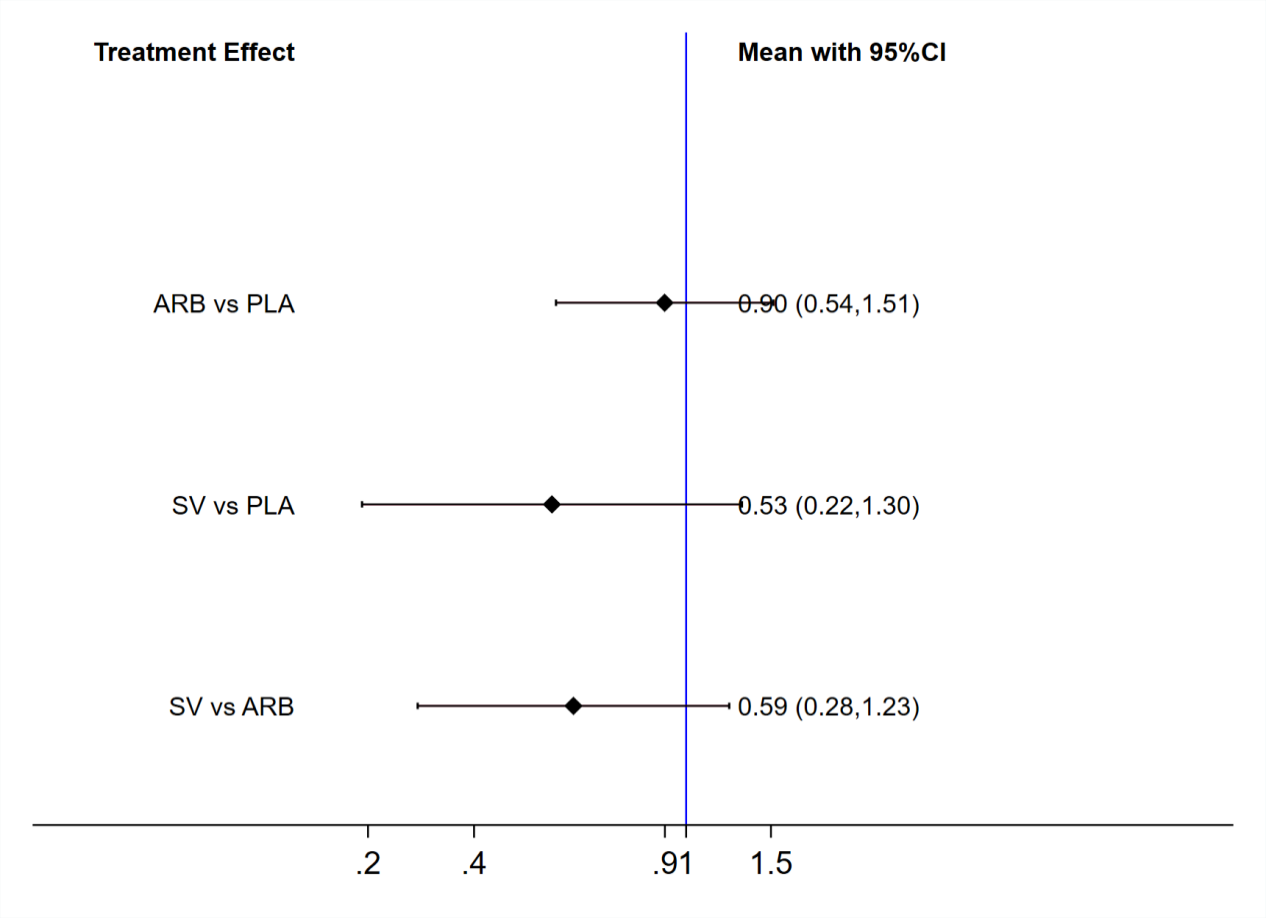
Figure S25.** The effect of sacubitril/valsartan (SV) compared to placebo (PLA) on elevated glycaemia among patients with not all-HF.


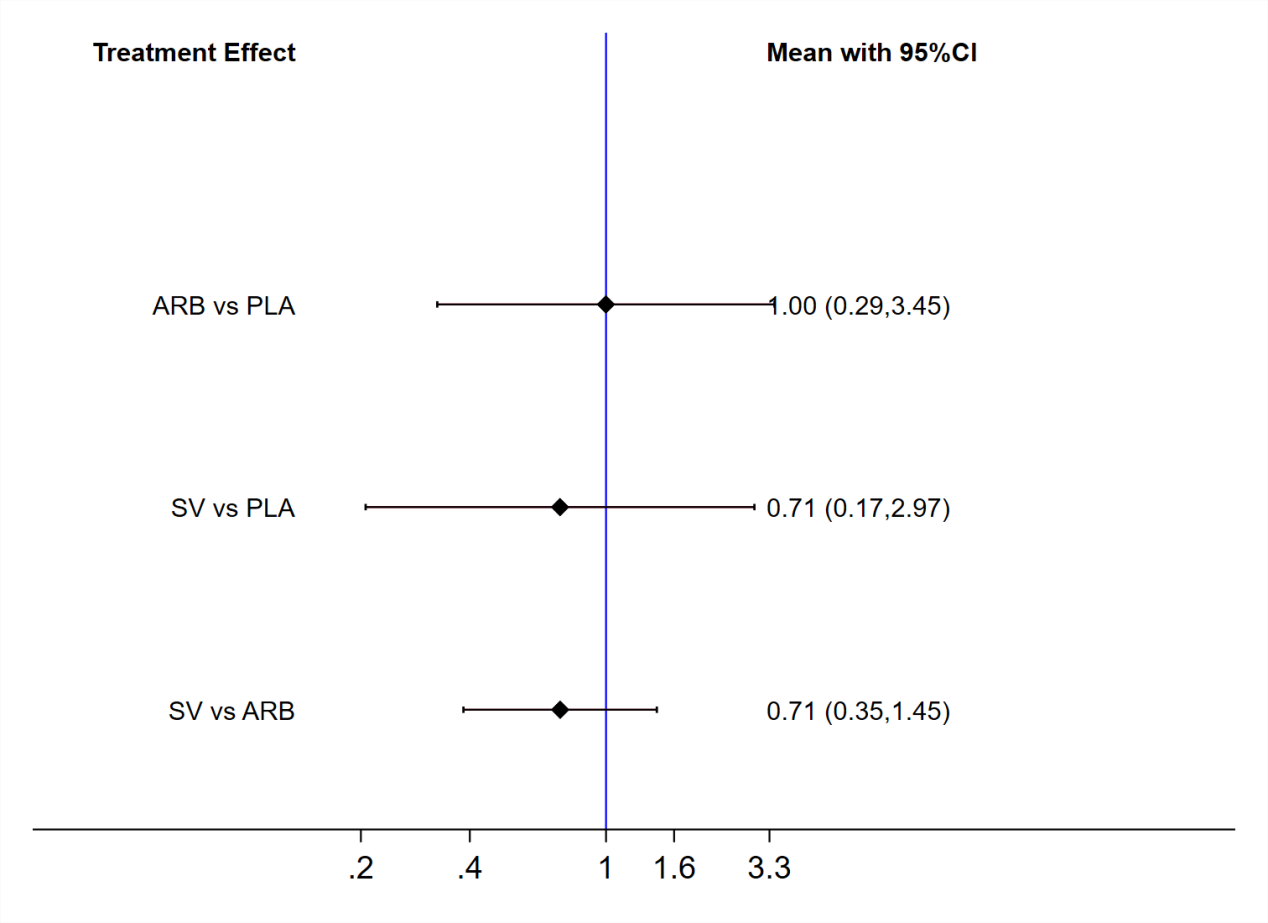


**Figure S26.** The effect of sacubitril/valsartan (SV) compared to placebo (PLA) on elevated glycaemia among patients with HFpEF.

**
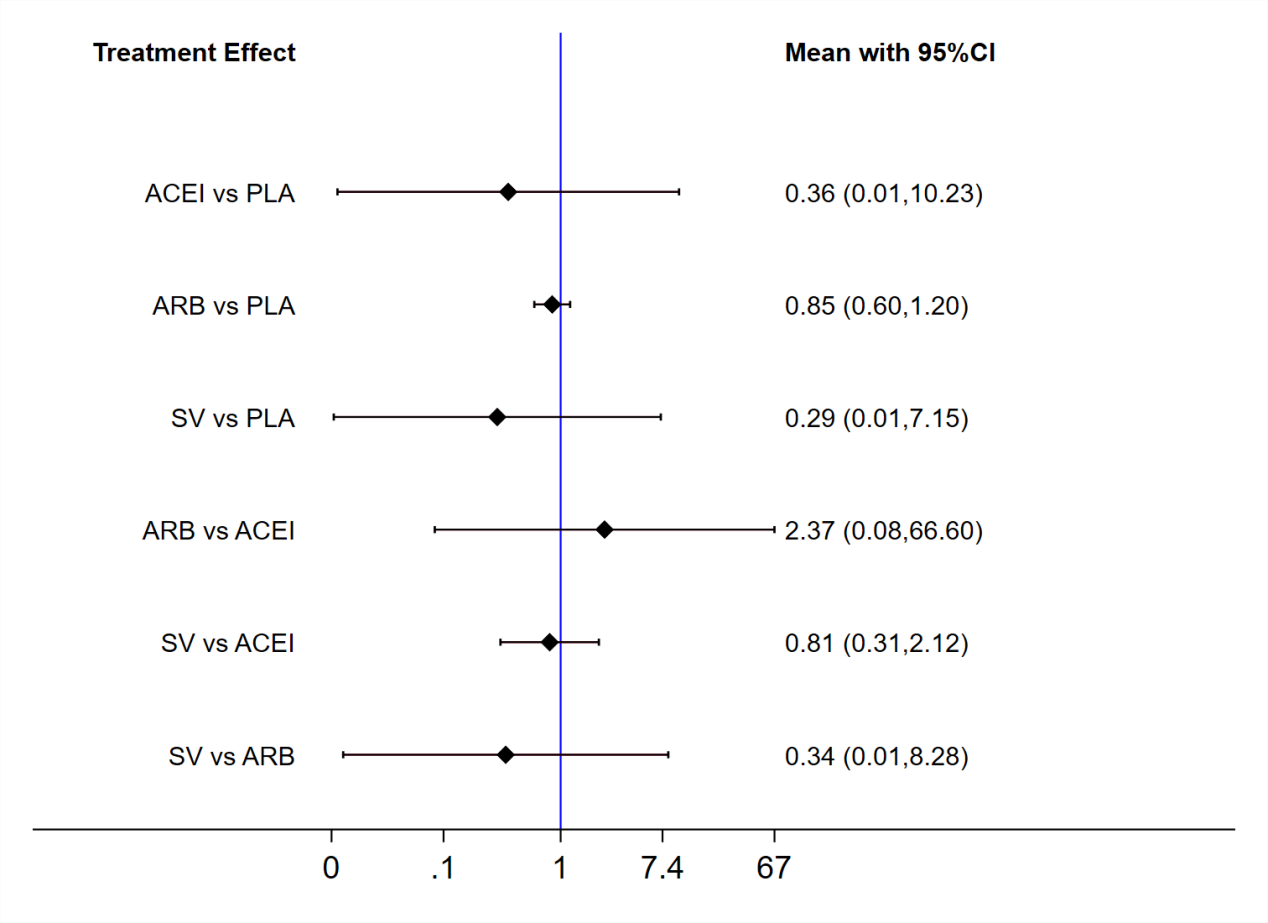
Figure S27.** The effect of sacubitril/valsartan (SV) compared to placebo (PLA) on DM inadequate control among all patients.


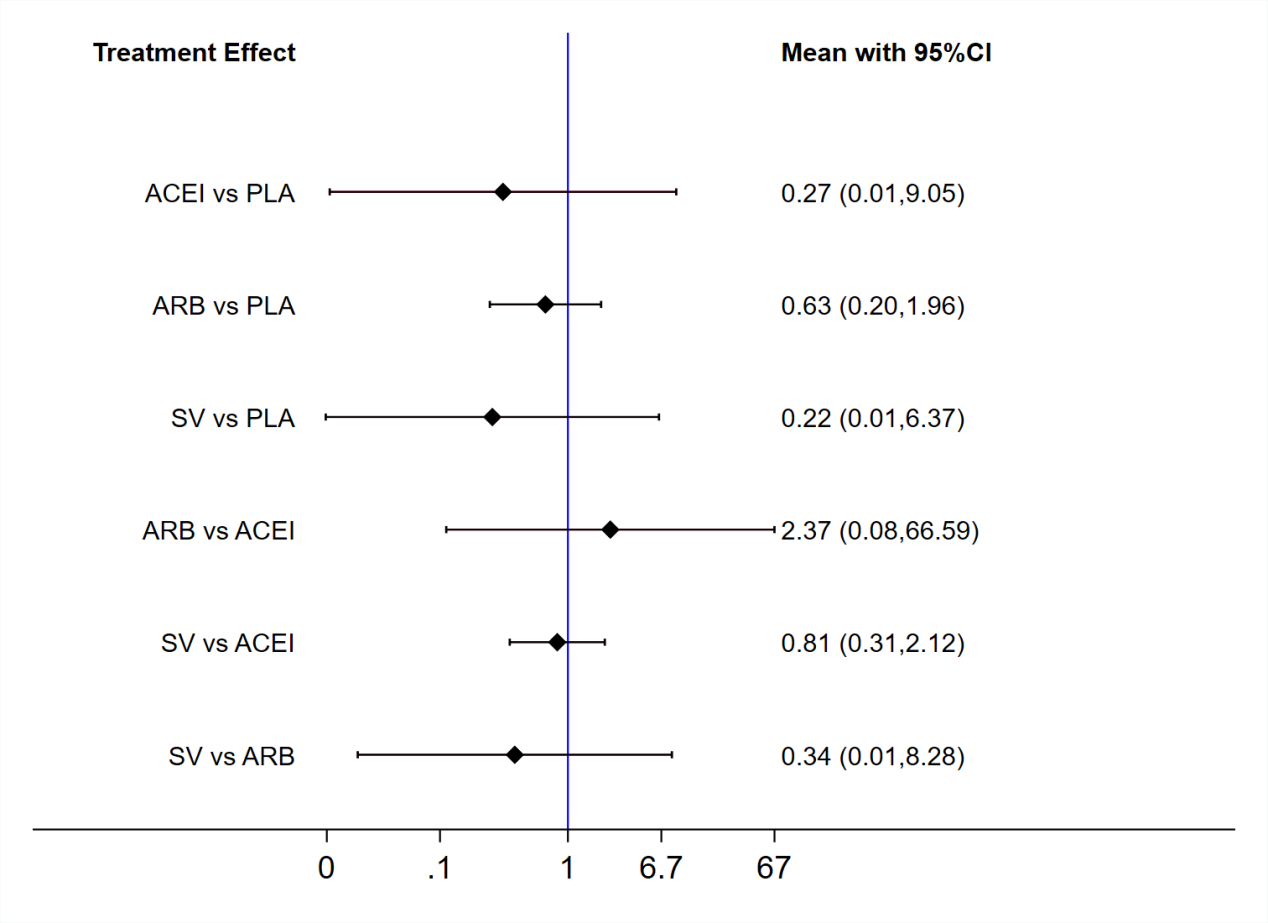


**Figure S28.** The effect of sacubitril/valsartan (SV) compared to placebo (PLA) on DM inadequate control among patients with not all-DM.

**
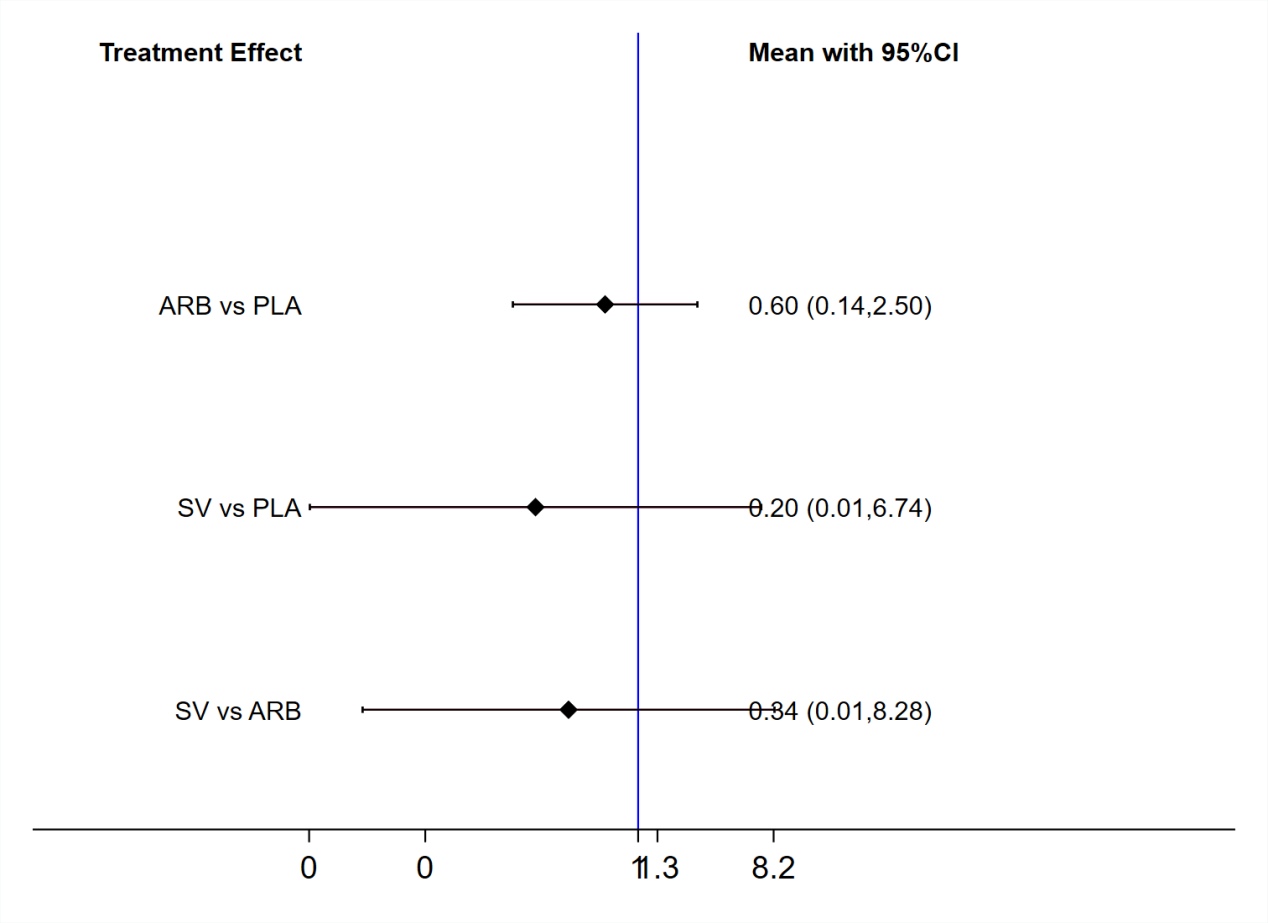
Figure S29.** The effect of sacubitril/valsartan (SV) compared to placebo (PLA) on DM inadequate control among patients with HFpEF.

**
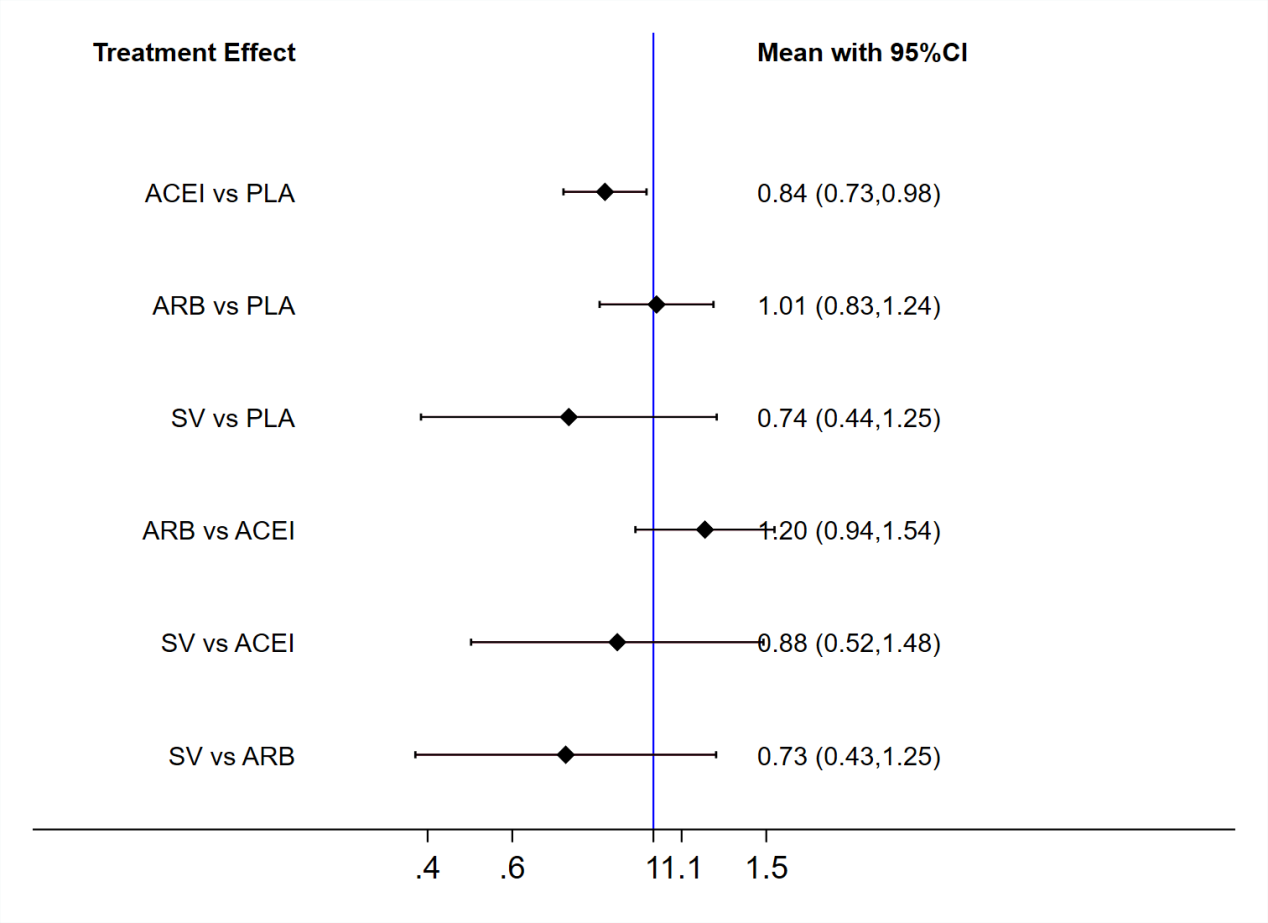
Figure S30.** The effect of sacubitril/valsartan (SV) compared to placebo (PLA) on diabetes complication among all patients.

**
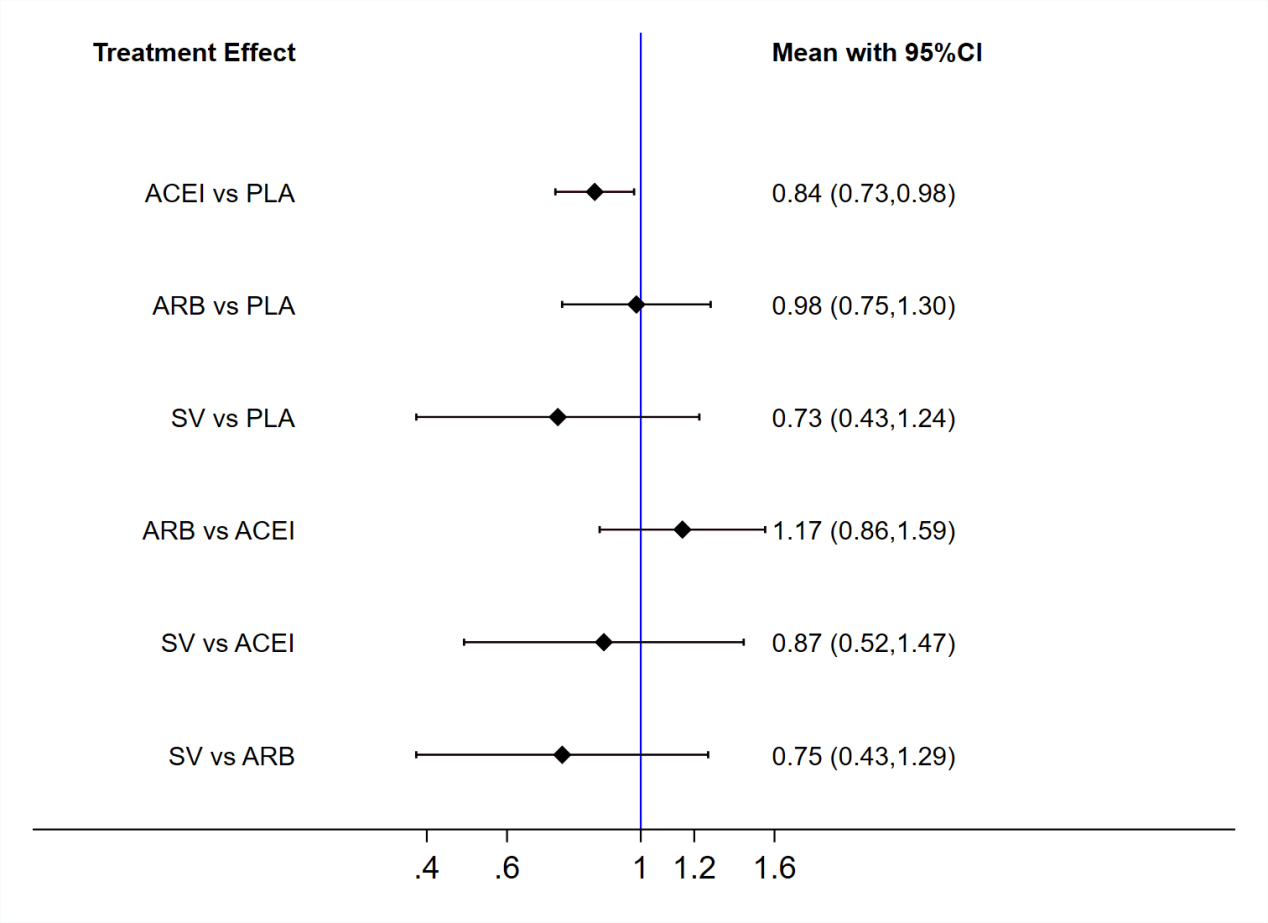
Figure S31.** The effect of sacubitril/valsartan (SV) compared to placebo (PLA) on diabetes complication among patients with not all-DM.

**
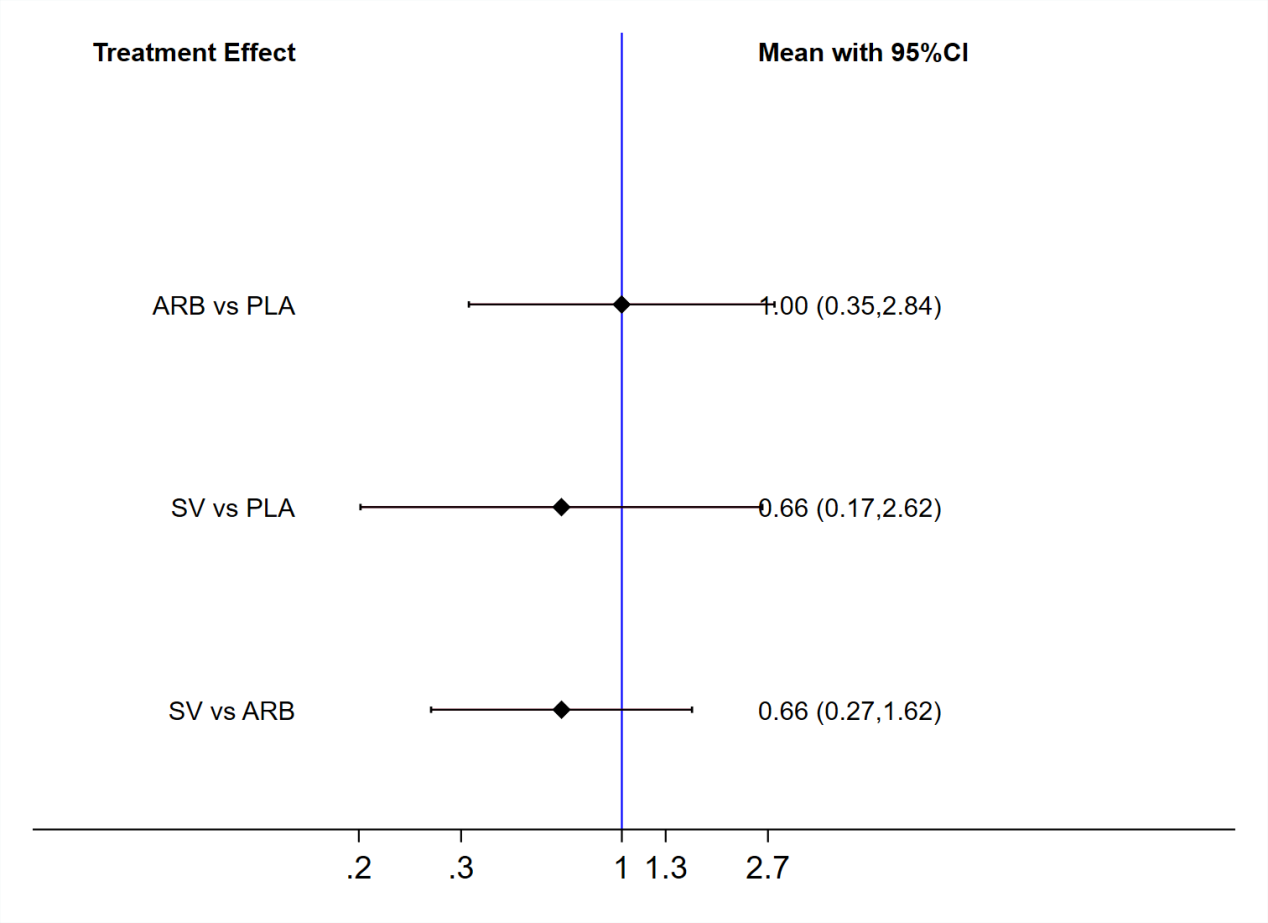
Figure S32.** The effect of sacubitril/valsartan (SV) compared to placebo (PLA) on diabetes complication among patients with HFpEF.

**
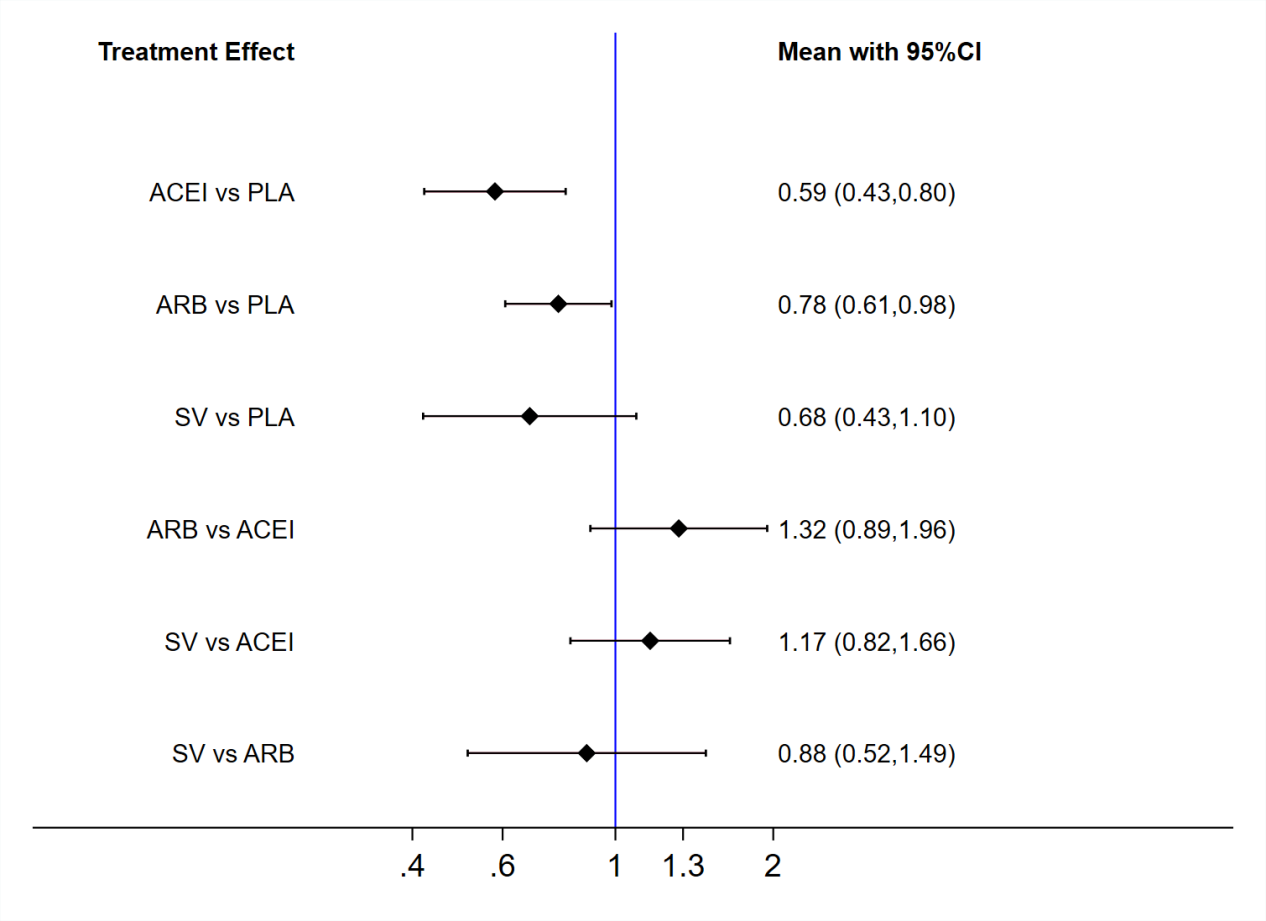
Figure S33.** The effect of sacubitril/valsartan (SV) compared to placebo (PLA) on diabetes treatment among patients with non-DM.
